# Addressing the Challenges of Identifying *Vibrio* Species; Genomic and Phenotypic Analyses of Misidentified *Vibrio vulnificus* Strains Reveal *Vibrio campbellii* and a Potentially Novel *V. rotiferianus*-like Species in New Zealand Oysters

**DOI:** 10.3390/microorganisms14081658

**Published:** 2026-07-29

**Authors:** Yuwei Zhang, Christopher Winefield, Graham C. Fletcher, Joseph Paul Robinson, Valery Patsekin, Travis R. Glare, Stephen L. W. On

**Affiliations:** 1 Department Wine, Food and Molecular Biosciences, Lincoln University, Lincoln 7647, New Zealand; yuwei.zhang@lincolnuni.ac.nz (Y.Z.); christopher.winefield@lincoln.ac.nz (C.W.); 2 New Zealand Institute for Bioeconomy Science Limited, Auckland 1142, New Zealand; graham.fletcher@plantandfood.co.nz; 3 New Zealand Food Safety and Science Research Center, Massey University, Palmerston North 4442, New Zealand; 4 Weldon School of Biomedical Engineering, Purdue University, West Lafayette, IN 47907-2032, USA; jpr@cyto.purdue.edu; 5 Department Basic Medical Sciences, Purdue University, West Lafayette, IN 47907-2032, USA; 6 Lincoln Agritech, Engineering Drive, Lincoln 7608, New Zealand; travis.glare@lincoln.ac.nz

**Keywords:** *Vibrio*, *Vibrio vulnificus*, *Vibrio campbellii*, *Vibrio rotiferianus*, identification, API20E, 16S rRNA, Elastic Light Scatter analysis, Whole Genome Sequencing, in silico DNA-DNA hybridization

## Abstract

The genus *Vibrio* contains over 150 species of aquatic origin, some of which are important foodborne pathogens, while others cause diseases in finfish, shellfish and/or coral. Accurate identification of these bacteria is important, but highly challenging given their taxonomic diversity. Here we used a range of methods to identify strains from New Zealand oysters initially identified as *Vibrio vulnificus.* The API20E kit either misidentified, or failed to identify the strains depending on the database used; comparisons of the 16S rRNA gene sequences could not distinguish them from over 50 exemplars of other *Vibrio* spp. or *Allocatenococcus thiocycli*. Elastic light scatter analysis showed clear differences between each of the novel isolates and reference strains of *Vibrio parahaemolyticus* and *V. vulnificus*, but limits of the in-house database precluded identification. Our whole-genome sequencing (WGS) pipeline allowed genomes and plasmids to be effectively sequenced to completion, enabling in silico DNA-DNA hybridization analysis with 162 *Vibrio* spp., which identified the strains as either *V. campbellii,* or one that may represent a novel species closely related to *V. rotiferianus.* Genome analysis also revealed a variety of genes found in aquacultural and human pathogens, including *V. cholerae.* Furthermore, resistance to multiple antibiotic classes was indicated both genetically and phenotypically. The implications for New Zealand aquaculture and public health are discussed. WGS is an effective identification strategy for taxonomically complex bacteria such as *Vibrio* spp.

## 1. Introduction

The genus *Vibrio* is a taxonomically complex group, containing 159 validly published species as of 5 June 2026 (https://lpsn.dsmz.de/genus/vibrio). Species are aquatic in nature and can be associated with freshwater or marine environments. A number of species (including *Vibrio cholerae*, *V. parahaemolyticus* and *V. vulnificus*) are established human pathogens [1,2], which can be transmitted by consumption of contaminated water, seafood (notably shellfish, where the filter-feeding mechanisms serve to concentrate the bacteria in the gills and digestive tract), and/or by introduction into skin lacerations, where fatal sepsis may occur [3]. Furthermore, a number of other species (including *Vibrio campbellii*, *Vibrio rotiferianus*, *Vibrio anguillarum* and *Vibrio alginolyticus*) pose threats to important fish species widely consumed as food [4,5,6]. Therefore, the accurate identification of *Vibrio* species is critical to human health, food safety and to aquacultural production; however, their complex taxonomy presents a major challenge in this regard.

The main focus of improvements in identification methods for *Vibrio* has been on species known to be important human or aquacultural pathogens, including *V. cholerae*, *V. parahaemolyticus*, *V. vulnificus*, *V. anguillarum*, *V. alginolyticus*, and species closely related to *V. harveyi* [7,8,9,10,11]. Despite technical advances, drawbacks including precision of speciation and ease of interpretation have been noted [7,8,11]. These deficiencies include an inability to discriminate closely related species, notably those in “the Harveyi clade” (i.e., *V. harveyi*, *V. alginolyticus*, *V. parahaemolyticus*, *Vibrio diabolicus*, *Vibrio rotiferianus*, *Vibrio owensii*, *Vibrio campbellii*, *Vibrio jasicida*, *Vibrio azureus*, *Vibrio sagamiensis*, *Vibrio natriegens*, and *Vibrio mytili*) [7]; failure to generate amplicons of specific markers (*pyrH*) in certain strains [7] and, with various Multi-Locus Sequence- and Matrix-Assisted Laser Desorption—Time-Of-Flight Mass Spectrometry and *fur* gene analyses, incorrect identifications [8]. The application of multiple species-specific PCR assays is considered unworkable [11].

In seeking to extensively characterize the pathogenomic properties of *V. vulnificus* isolates from New Zealand shellfish, it became apparent that several strains submitted to us as *V. vulnificus* were other *Vibrio* species. This paper describes the phenotypic and genotypic methods we used to characterize these anomalous isolates, to discuss practices for bacterial identification generally, especially for large, taxonomically complex groups. Our results identify *Vibrio* species that have not to our knowledge been described before in New Zealand, that may have implications for the nation’s aquaculture.

## 2. Materials and Methods

### 2.1. Strains

Isolates 30D07 and 31D03 were supplied as part of a collection of 38 strains that had been previously identified as *V. vulnificus* (Cruz et al., 2016) [12]. Their aberrant nature in that collection was first identified in the Elastic Light Scatter analysis, then subsequently using whole genome sequencing, described here.

### 2.2. Biochemical Test Profiling

The API 20E test kit (Biomerieux, Salt Lake City, UT, USA) was used to examine strains for their ability to produce 11 enzymes and metabolize nine substrates, as described previously [13], preparing bacterial suspensions in 2% saline solution, as recommended for *Vibrio* species identification by the kit manufacturer. Results were interpreted according to manufacturer’s guidelines and compared with pre-existing databases dedicated to analysis of these data: APIWEB, the manufacturer’s own database (V5.0, https://apiweb.biomerieux.com/strip/1; accessed on 25 May 2025); and BACDIVE, an independent database hosted by the German Collection of Microorganisms and Cell Cultures (DSMZ, Braunschweig-Süd, Germany) (https://bacdive.dsmz.de/api-test-finder; accessed on 25 May 2025).

### 2.3. Whole Genome Sequencing

#### 2.3.1. Genomic DNA Extraction

*Vibrio* strains used for genomic DNA (gDNA) extraction were subcultured twice on Brain Heart Infusion (BHI, Oxoid [Basingstoke, UK], supplemented to attain 2.0% (*w*/*v*) total NaCl concentration) agar at 37 °C for 24 h. A single colony of each strain was inoculated into 25 mL Brain Heart Infusion (Oxoid, supplemented to attain 2.0% (*w*/*v*) total NaCl concentration) and incubated at 37 °C overnight with shaking at 100 rpm. To prepare the broth cultures for subsequent DNA extraction, bacterial cells were pelleted by centrifugation and washed three times in sterile phosphate-buffered saline (PBS) prior to DNA extraction. gDNA of each strain was extracted using GenElute™ Bacterial Genomic DNA Kit (Sigma, St. Louis, MI, USA) following the manufacturer’s protocol. A magnetic bead cleaning and concentration approach was then applied by treating 150–250 µL (8–10 µg) of gDNA prep of each strain with equal volumes of well-mixed Mag-Bind^®^ TotalPure NGS magnetic beads (Omega Bio-tek, Norcross, GA, USA), to initiate the elimination of extremely small DNA fragments (<400 bp) and non-DNA impurities, shorten long DNA fragments (>50,000 bp) to 35,000–45,000 bp, as well as pre-concentrate preparations for subsequent size selection. Any shearing or small DNA fragments < 10 kb in length, which can interfere with the sequencing efficiency of MinION, were subsequently eliminated by using a Short Read Eliminator Kit (Illumina, San Diego, CA, USA) with a 50–70% or even higher DNA recovery efficiency.

The gDNA sample quality was determined using a DeNovix DS-11 Series Spectrophotometer/Fluorometer (Denovix, Wilmington, DE, USA) and a Qubit fluorometer 3.0 (Thermo Fisher Scientific, Waltham, MA, USA), respectively. The acceptable 260:280 ratio of gDNA in this study is expected in a range between 1.76 and 1.95, while the corresponding 260:230 ratio should be above 2.00. Quality of DNA fragments and the average length of long fragments was determined by using an Agilent Genomic DNA 50 kb Kit (Agilent, Santa Clara, CA, USA) under the analysis of an Advanced Analytical Fragment Analyzer (Agilent, USA) through evaluating the gDNA sizing and integrity. All operations followed appropriate manufacturers’ instructions for the kits or equipment. All prepared gDNA samples were kept on ice until the preparation of sequencing libraries.

#### 2.3.2. Sequencing, Genome Assembly, Annotation, 16S rRNA Gene Sequence Comparisons, In Silico Virulence- and Antimicrobial Resistance Gene Prediction and In Silico DNA-DNA Hybridisation

The MinION platform (Oxford Nanopore, Oxford, UK) was used as per the manufacturer’s recommendations for the Native barcoding genomic DNA scheme, with EXP-NBD 104, EXP-NBD114 & SQK-LSK109 kits. The MinION sequencing library in this study was prepared by pooling the gDNA samples of 30D07 and 31D03 with those of another 10 isolates of *Yersinia* spp. and *Vibrio* spp. (five *Yersinia* spp. strains and five New Zealand oyster isolates of *Vibrio* spp.) in multiplex, with a sequencing time of 72 h using MinKNOW software v24.11.8 (Oxford Nanopore). We tested the feasibility of using MinION technology to generate high-quality long-read outputs of complex bacterial DNA samples in a single run. The sequencing-produced raw read set was basecalled and demultiplexed using Guppy basecaller and debarcoder tools (Oxford Nanopore), respectively, with a specified quality filtration parameter (Q-score ≥ 10). Individual read sets of ≥30 kb of 30D07 and 31D03, respectively, were selected using Filtlong (v0.2.1). Contiguous sequences (genome and plasmid data) were assembled using Trycycler v0.5.3 [14] with three assemblers, namely Flye (v2.9) [15], Raven (v1.6.0) [16] and wtdbg2 (v2.5) [17]. Filtlong was used for pre-assembling read quality control to discard short reads below 1 kb and the shortest 2% of the input reads. The input reads of each sample were subsampled into 12 read subsets using Trycycler subsampler with default setting before assembly. Consensus contig sequences were generated with the built-in consensus command of Trycycler and polished using Medaka (v1.5.0). The completeness of assembled genomes was evaluated by using Benchmarking Universal Single-Copy Orthologs (BUSCO v.5.2.2) [18] at an order level with a completeness threshold of 90% for a complete genome. The genomes were annotated using National Center for Biotechnology Information (NCBI) Prokaryotic Genome Annotation Pipeline (PGAP) [19,20]. Functional classifications of coding genes were carried out using EggNOG (v5.0) [21] and BlastKOALA (KEGG Orthology And Links Annotation) [22] against the Clusters of Orthologous Genes (COG) [23] and the Kyoto Encyclopedia of Genes and Genomes (KEGG) database [24], respectively (accessed 14 September 2025). Virulence genes were also screened for using the Virulence Factor DataBase (VFDB) [25] (accessed 15 December 2025). Genes associated with resistance to antimicrobial agents were identified using the “Perfect or Strict” matching algorithm in the Comprehensive Antibiotic Resistance Database (CARD) [26] (accessed 13 March 2026).

Full length 16S rRNA gene sequence from each strain was subsequently extracted from the assembled genome using Geneious Prime v2022.0.2 (Auckland, New Zealand), and compared with those of taxa in the dedicated 16S database hosted by the National Center for Biotechnology Information using the Megablast algorithm (https://blast.ncbi.nlm.nih.gov/Blast.cgi, accessed on 23 May 2025).

In silico DNA-DNA hybridisation (isDDH) analyses between our strains and those of NCBI reference strains of 162 *Vibrio* species were performed using the online Genome-to-Genome Distance Calculator (GGDC 3.0, https://ggdc.dsmz.de, accessed on 27 June 2025) [27]. Furthermore, given these results, pair-wise average nucleotide identity (ANI) analyses were conducted across the genomes of our strains and the reference genomes of 21 *Vibrio* spp., including those of the *Vibrio harveyi* clade which includes *V. campbellii* and *V. rotiferianus* [28]. Complete genome sequences of these strains were downloaded from the NCBI database. Pair-wise average nucleotide identity (ANI) analyses were conducted across the genomes of our strains and the 21 reference genomes using the JSpeciesWS online service (https://jspecies.ribohost.com/jspeciesws/#home; v. 5.0.3. Ribocon, Bremen, Germany, accessed on 19 August 2025) with the ANI calculation based on BLAST+ (ANIb) and MUMmer (ANIm), respectively. The ANI results were plotted into dendrograms with maximum likelihood trees using Heatmap2 (Galaxy Version 3.2.0+galaxy1) on the Galaxy platform (https://usegalaxy.org/, accessed on 19 August 2025).

### 2.4. Elastic Light Scatter (ELS) Profiling

Isolates were cultured on heavy-filled (25 mL) Brain Heart Infusion + 2.0% NaCl agar media for 12 h at 37 °C and subjected to ELS profiling as described previously [29]. ELS profiles were compared with comparable results from representative strains of *V. parahaemolyticus* and *V. vulnificus* using Baclan software V12.19 (Purdue University, West Lafayette, USA).

### 2.5. Antimicrobial Susceptibility Testing

Antimicrobial susceptibility profiles of the *Vibrio* strains were tested using the Kirby-Bauer disk diffusion method [30] with minor modifications. In brief, a PBS suspension of the twice-subcultured colonies of each strain (37 °C for 24 h on BHI + 2.0% NaCl agar) was standardized to 0.5 McFarland and, using a sterile swab, spread on Mueller-Hinton (MH) agar which was supplemented with 2.0% NaCl. Antibiotic disks (Oxoid, UK) were placed on the surface of agar and the plates were incubated at 35 °C for 24 h. The diameter of bacterial inhibition zone formed surrounding each disk was measured (mm) using a ruler. The results were interpreted according to Clinical and Laboratory Institute (CLSI) guidelines or proposals [31,32] and recorded as susceptible or resistant. Thirteen antibiotics were tested in the study: azithromycin (15 μg), nalidixic acid (30 μg), cephalothin (30 μg), gentamicin (10 μg), cefoxitin (30 μg), ciprofloxacin (5 μg), erythromycin (15 μg), meropenem (10 μg), penicillin G (5 μg), tetracycline (30 μg), chloramphenicol (30 μg), kanamycin (30 μg) and ampicillin (2 μg and 10 μg).

### 2.6. Motility Assays

The motility assays were conducted using Luria–Bertani (LB) medium with 2% NaCl and 0.3% agar and BHI with 2% NaCl and 0.6% agar for swimming and swarming motilities, respectively. To evaluate swimming motility, a single colony was picked with a sterile inoculation needle and stabbed 5 cm deep into the center of 25 mL of semi-liquid agar medium, which was set in a translucent 50 mL centrifuge tube. The size of the swimming zone developed inside the agar was measured (mm) using a caliper after 24 h incubation at room temperature (24 ± 1 °C). For the swarming assay, a 3 μL aliquot of standardized overnight culture (OD_600_ = 1.0) was spot inoculated onto the center surface of an agar plate filled with 20 mL of the medium. Plates were incubated at 37 °C for 24 h to measure the diameters (mm) of swarming halos. Both swimming and swarming assays of each strain were triplicated.

### 2.7. Haemolytic Assay

A single colony of each *Vibrio* isolate was streaked on Columbia Horse Blood (5%) Agar (Fort Richard, Auckland, New Zealand) and incubated at room temperature (24 ± 1 °C) and 37 °C for up to 48 h. The haemolysis effects, namely α- (partial), β- (complete), or γ- (no) haemolysis, were determined according to the observation of haemolytic zones around the colony cultures on plates, as described elsewhere [33].

## 3. Results

### 3.1. API Profile Results

Isolate 30D07 generated the API strip code 5046105, and isolate 31D03 the code 5044005. Both isolates produced β-galactosidase, lysine decarboxylase, indole and NO_2_, and fermented glucose and amygdalin. Only 30D07 produced gelatinase and fermented mannitol. Neither of the strains produced arginine dihydrolase, ornithine decarboxylase, citrate simmons, hydrogen sulfide, urease, tryptophan deaminase, Voges-Proskauer, or produced acid from inositol, sorbitol, rhamnose, sucrose, melibiose, or arabinose. Using the official API 20E database (tested 23 May 2025), 30D07 and 31D03 were identified as *V. vulnificus* with identification scores of 99.4% and 99.2%, respectively. However, these results were not assigned to any named taxon in the “Bacdive” database (tested 26 May 2025).

### 3.2. 16S rRNA Gene Sequence Analysis

The full length 16S rRNA gene was extracted from the genomes of strains 30D07 and 31D03, and submitted to the NCBI BLAST database on 23 May 2025 for bacterial sequences of this type, using the most stringent matching algorithm available (Megablast). In both cases, no definitive species identification could be attained, since by any of the standard metrics such as E-values or percentage similarity, matches for up to 59 *Vibrio* species were obtained. For strain 30D07, E-values of 0.0 were obtained for each of 59 *Vibrio* species, and one of *Allocatenococcus thiocycli* with the lowest percentage similarity attained being 95.48% against *Vibrio lentus* (Supplementary Table S1). For strain 31D03, E-values of 0.0 were obtained for each of 50 *Vibrio* species, and one of *Allocatenococcus thiocycli*, with the lowest percentage similarity attained being 94.99% (Supplementary Table S2).

### 3.3. Elastic Light Scatter (ELS) Profiling

**Figure 1:**
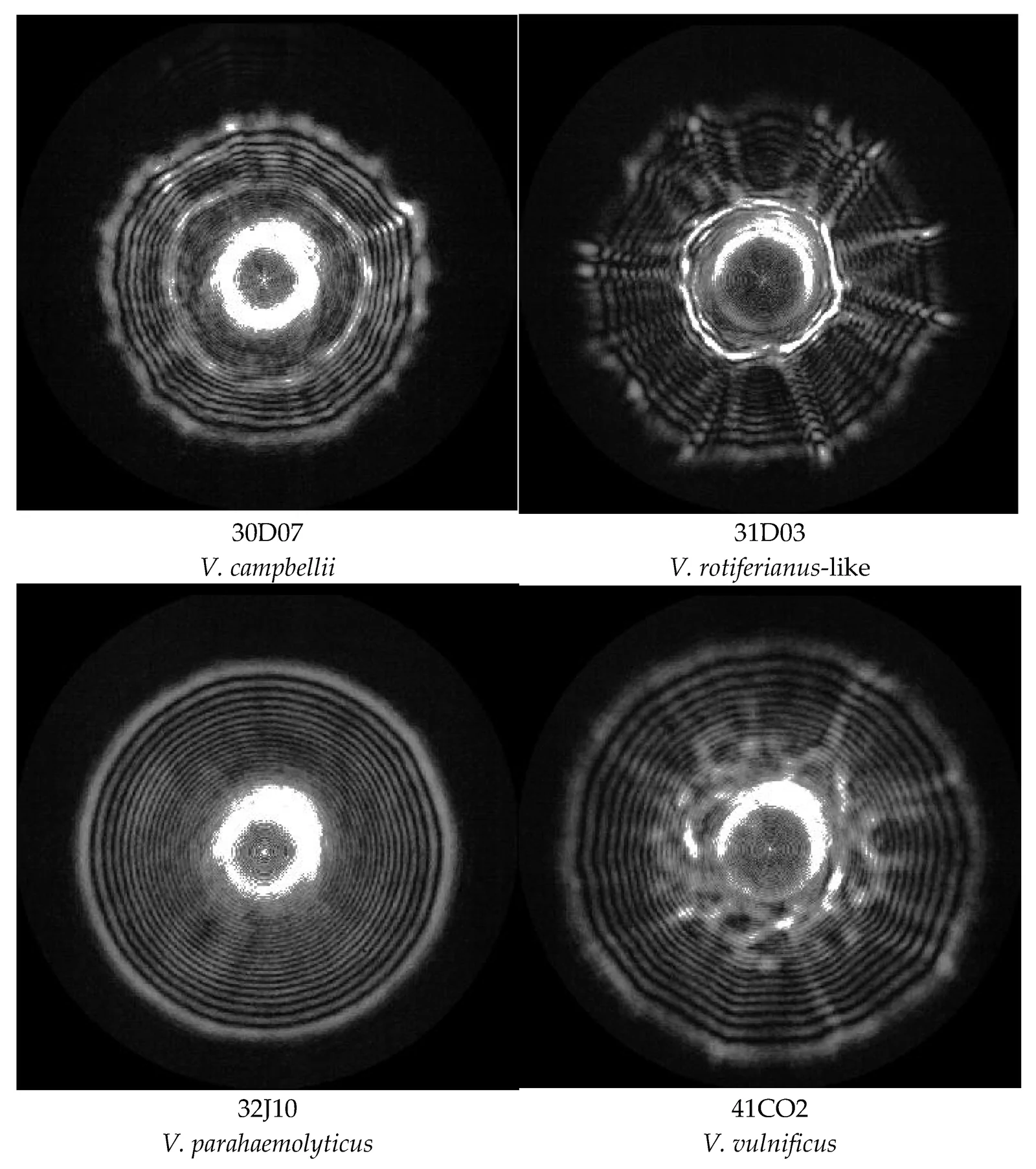
Representative Elastic Light Scatter profiles of New Zealand isolates 30D07 (*Vibrio campbellii*), 31D03 (*V. rotiferianus*-like), *V. parahaemolyticus* and *V. vulnificus*.

The ELS *Vibrio* profile database comprised 250 individual spectra derived from multiple experiments on the taxa examined (Table 1). The ELS profiles for strains 30D07 and 31D03 were visually distinct from those previously described [34], and in our in-house database of *V. parahaemolyticus* and *V. vulnificus* (Figure 1) isolates. The Coefficient of Variance matrix (Table 1) also revealed each of these taxa to be distinguished from each other, with homologous comparisons reaching values exceeding 90%, as defined previously as a suitable threshold value for separation of taxa with this method [29].

### 3.4. Genome Characteristics

A total number of 44,639 (796,069,103 bp) and 95,281 (1,211,018,351 bp) long-reads were generated for strains 30D07 and 31D03, respectively. The N50s for strain 30D07 and 31D03 were 26,079 bp and 21,085 bp, respectively, through the MinION sequencing run with a N50 of 23,598 bp. Raw sequence reads were assembled into three contigs in a total size of 5,754,055 bp with an overall GC content of 45.6% and a 144× coverage for strain 30D07, and two contigs in a total size of 5,154,655 bp with an overall GC content of 44.6% and a 243× coverage for strain 31D03. The mean sequencing, polishing and assembly time per strain was approximately 24 h. Circularized genome assemblies were produced for both the strains.

The genome of strain 30D07 includes two chromosomes of 3,530,783 bp and 2,192,494 bp with GC content of 45.4–45.7%, and a 30,778 bp plasmid with a 42.9% GC content. PGAP identified 4979 coding genes (3058 on Chromosome I and 1921 on Chromosome II), 34 rRNAs (31 on Chromosome I and three on Chromosome II), 131 tRNAs (116 on Chromosome I and 15 on Chromosome II), one tmRNA (Chromosome I) and three ncRNAs (Chromosome I) predicted on the chromosome region. Additionally, 28 coding genes were annotated on the 30,778 bp plasmid.

The genome of strain 31D03 consists of a 3,295,458 bp chromosome I and a 1,859,197 bp chromosome II with GC contents of 44.7% and 44.4%, respectively. PGAP predicted 4702 coding regions, including 4527 coding genes (2921 on Chromosome I and 1606 on Chromosome II), 37 rRNAs (34 on Chromosome I and 3 on Chromosome II), 134 tRNAs (119 on Chromosome I and 15 on Chromosome II), one tmRNA (Chromosome I) and three ncRNAs (Chromosome II).

Completeness scores of 93.5% and 96.3% were assigned to the genomes of 30D07 and 31D03 by BUSCO analysis, respectively, which were both above the 90% cut-off for noting a genome as complete.

**Figure 2:**
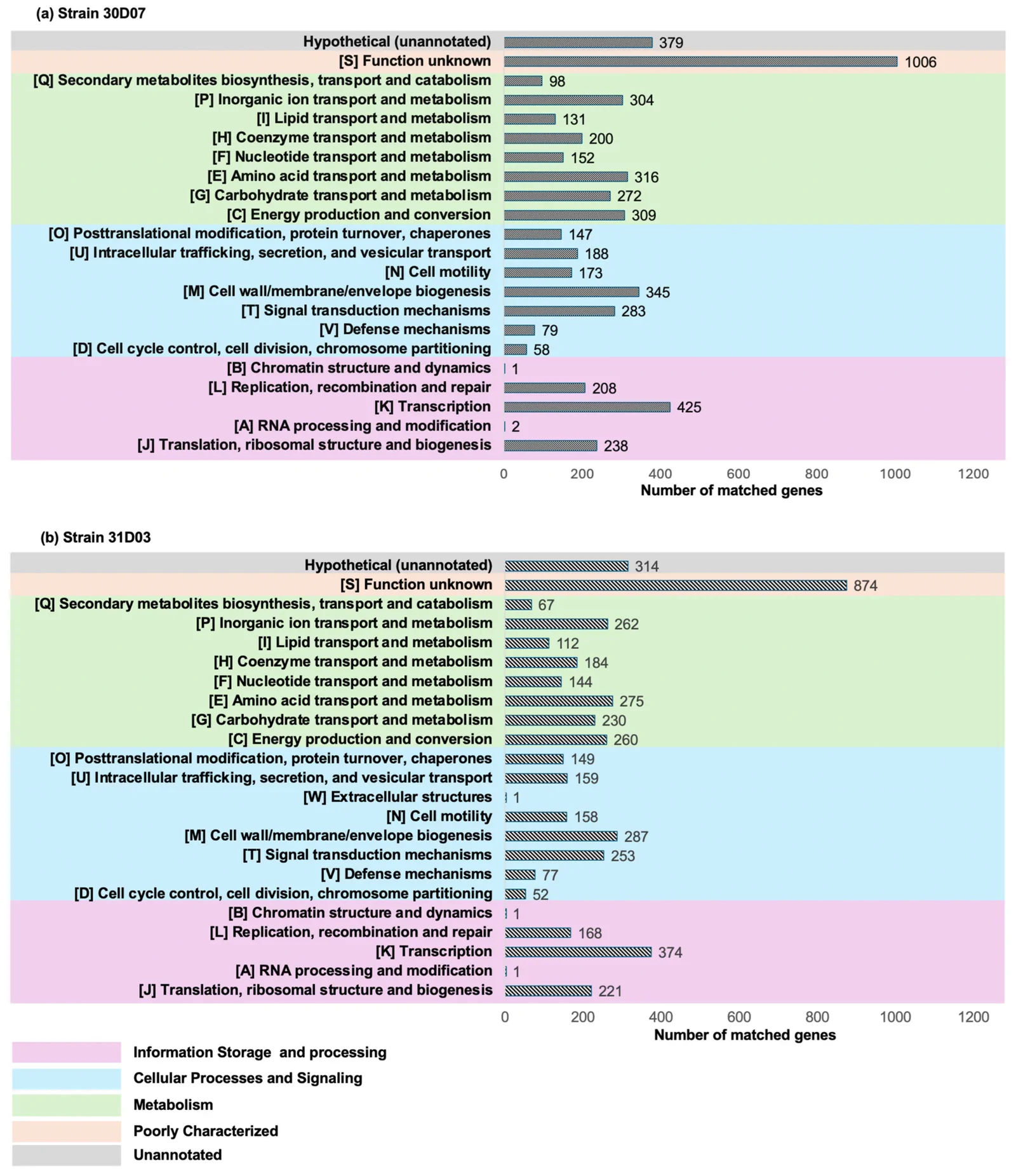
COG functional annotation of coding genes in the genome of (**a**) strain 30D07 and (**b**) strain 31D03.

**Figure 3:**
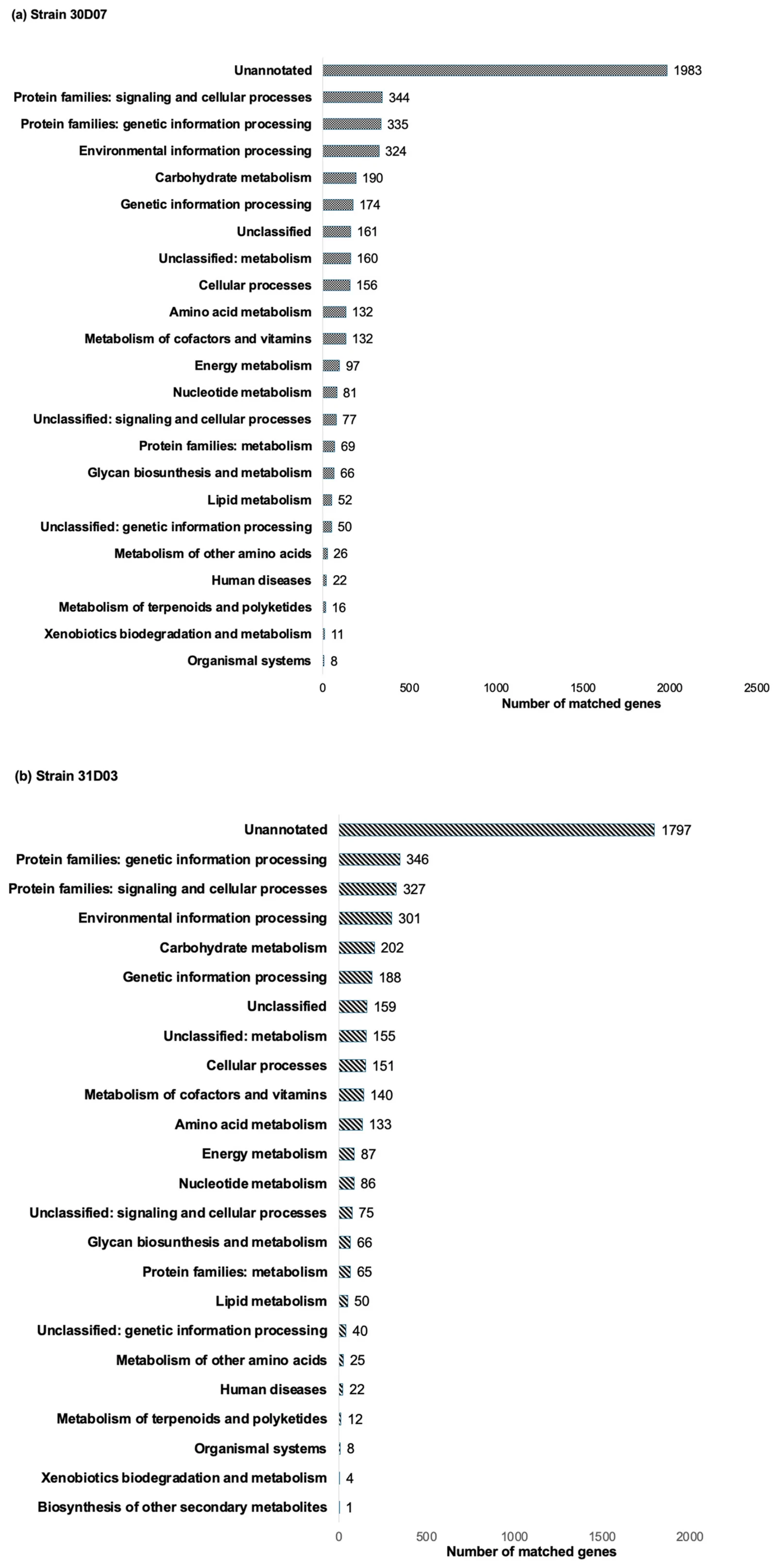
The KEGG functional annotation of the genome of (**a**) strain 30D07 and (**b**) strain 31D03.

**Figure 4:**
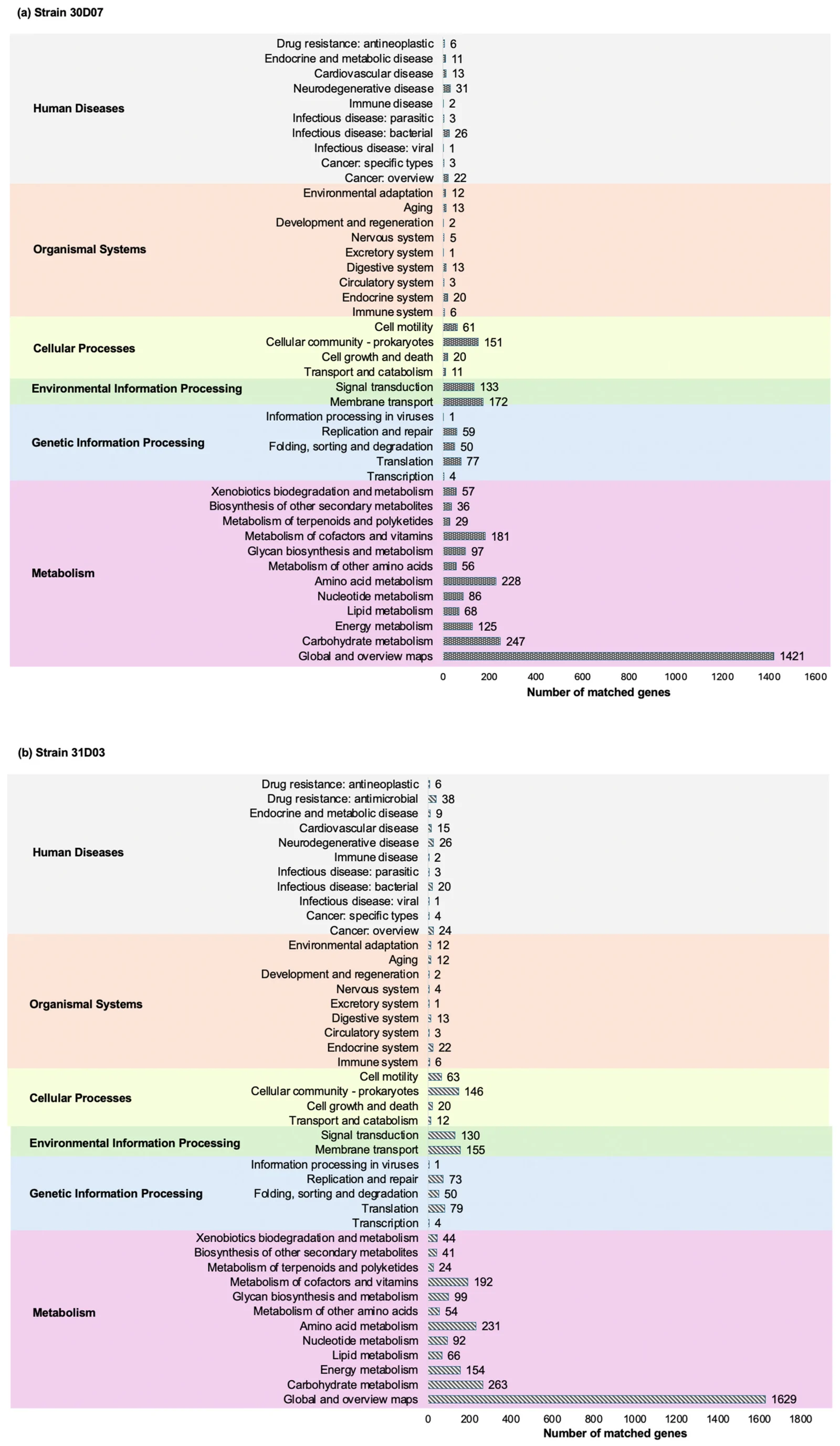
The KEGG pathway annotation results of (**a**) strain 30D07 and (**b**) 31D03. The ordinates indicate the level 2 KEGG pathway categories, and the abscissas indicate the number of coding genes annotated under each of the categories. Different colors represent the level 1 KEGG categories which are labeled.

COG annotation detected a total of 4997 genes in the genome of strain 30D07 and 4337 genes in the genome of strain 31D03, of which 4661 (93.3%) and 4023 (92.8%) were assigned into 21 and 22 functional groups, respectively (Figure 2). The KEGG annotation results showed an annotation rate of 57.5% and 59.5% in the 4666 and 4440 entries detected in strain 30D07 and 31D03, respectively. Besides the 22 KEGG functional categories assigned to the annotated genes of strain 30D07, one more KEGG category, namely, the biosynthesis of other secondary metabolites, which consisted of one gene only, was found among the annotated genes of strain 31D03 (Figure 3). The KEGG pathway analysis further assigned the annotated genes to 256 and 253 known pathways for strain 30D07 and 31D03, respectively, of six pathway categories, namely metabolism, genetic information processing, environmental information processing, cellular processes, organismal systems and human diseases (Supplementary Table S3). Notably, 26 and 20 orthologs linked to 10 and 9 pathways of bacterial infections in humans were indicated in strains 30D07 and 31D03, respectively, including that of *Vibrio cholerae* infection which were assigned with two genes of strain 30D07 and one gene of strain 31D03 (Supplementary Table S3). Similarly, the Virulence Factor Database identified 148 and 109 genes respectively in strains 30D07 and 31D03, supporting the virulence potential of the strains (Supplementary Table S4). The KEGG pathway annotation results of strain 30D07 and 31D03 at the Level 1 and Level 2 KEGG pathway categories are summarized in Figure 4.

### 3.5. In Silico DNA-DNA Hybridisation

We compared our genome sequences with those of type and reference strains of 162 *Vibrio* species using the GGDC online tool that models DNA-DNA hybridisation experiments, for which a threshold value of 70% matching similarity is used to designate strains belonging to the same species [35]. For 30D07, each of the three formulae resulted in values above 70% homology with the NCBI reference genome (GCA_002906475.1) of *V. campbellii*, providing unambiguous identification. For 31D03, two of the three formulae yielded values exceeding 70% similarity with the NCBI reference genome (GCA_002214395.1) of *V. rotiferianus,* but Formula 2 results fell far short of this value (42.9, confidence intervals 40.4–45.5) with this species. This ambiguity leads us to describe the strain as *V. rotiferianus*-like. Our results are provided in Supplementary Table S5.

### 3.6. ANI Analysis

**Figure 5:**
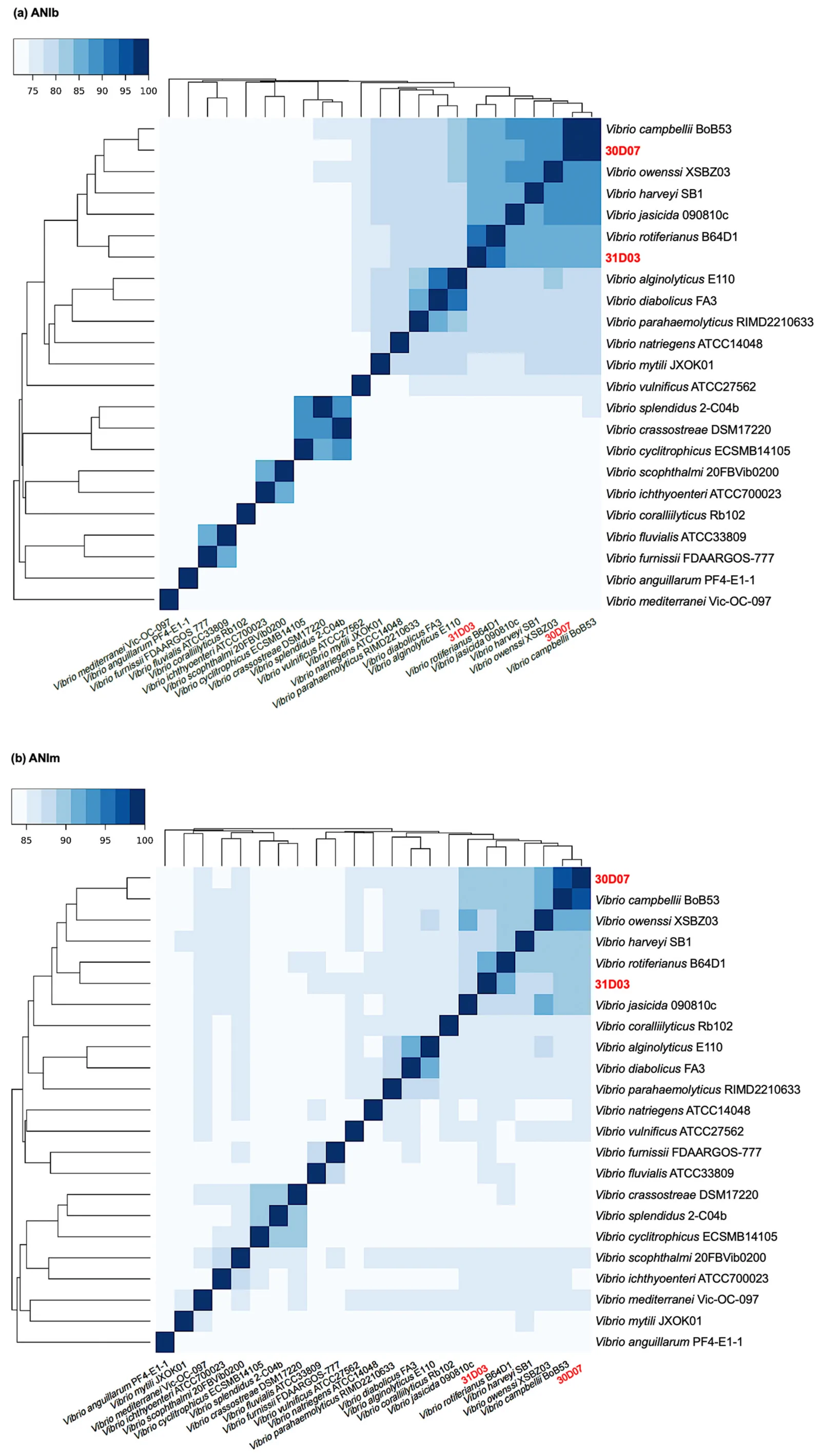
Phylogenetic analysis across genomes of strain 30D07, strain 31D03 and the NCBI reference strains representing 21 *Vibrio* spp. based on (**a**) ANIb and (**b**) ANIm analysis results.

The ANI value of 95% is commonly used as the threshold for identifications at the prokaryotic species level [36]. ANIb and ANIm results between strain 30D07, strain 31D03 and the NCBI reference genomes of 21 *Vibrio* species (Supplementary Table S6) are summarized in Figure 5, which were unsurprisingly comparable not only between the two ANI methods but also to our DNA-DNA hybridization results. Both ANIb and ANIm analyses generated below-threshold ANI values (85.41–85.5% by ANIb and 89.19–89.2% by ANIm) between strain 30D07 and 31D03, suggesting our two strains belong to two different species. For strain 30D07, both the methods produced ANI values (96.77–96.89% by ANIb and 97.2% by ANIm) above 95% with the genome of NCBI *V. campbellii* reference strain BoB53 (GCA_002906475.1) only, hence strain 30D07 was considered to come from this species. For strain 31D03, none of the reference genomes presented an ANI above 95% with its genome, regardless of the algorithms used. Among genomes of the 21 reference *Vibrio* species including those belongs to the *V. harveyi* clade, only the genome of NCBI *V. rotiferianus* reference strain B64D1 got ANI values (90.63–90.67% by ANIb and 91.43–91.44% by ANIm) above 90%. These results supported our description of strain 31D03 as a *V. rotiferianus*-like strain.

### 3.7. Prediction of Genes Associated with Antimicrobial Resistance (AMR)

The CARD [26] analysis indicated six AMR genes in each strain, with *adeF* and *parE* absent in 30D07, and PBP3 and FosA8 absent in 31D03 (Table 2). However, the percentage identity of seven such genes was relatively low (≤50.75).

### 3.8. Antimicrobial Susceptibility Testing

The antimicrobial susceptivity of strain 30D07 and 31D03 was tested against 13 antibiotics, as shown in Table 3. The antimicrobial susceptibility profiles of the two *Vibrio* strains were found to be nearly identical. Using what we considered to be the most appropriate interpretative criteria (Table 3, refs. [27,28]), of the antibiotics tested, only sensitivity to tetracycline was determined, and this was only for strain 31D03. Otherwise, the strains could be considered resistant to the remaining antimicrobial agents of β-lactam, aminoglycoside-, carbapenem-, cephalosporin-, macrolide-, fluoriquinolone-, phenicols and quinolones classes, although their tolerance to ciprofloxacin, chloramphenicol, nalidixidic acid and tetracycline (for 30D07) was relatively weak. Extremely high resistance to cefoxitin and the two β-lactam antibiotics was exhibited by the strains, with no inhibition zones observed around the disks of ampicillin, penicillin G and cefoxitin.

The correlation of these phenotypic results with AMR genes identified by CARD analysis (3.7 above) with percentage identity scores exceeding 70 appears reasonably sound, given that *CRP* is characterized as an efflux pump conferring resistance to multiple antimicrobials. However, the sensitivity of 31D03 to tetracycline did not correlate with the prediction of the TxR—ATP-binding cassette in this strain. Neither of the *Vibrio* species indicated in this study are represented in the CARD, which may explain this discrepancy. Equally, no formalized guidelines for interpreting antibiotic disk diffusion results for these taxa are extant and so must also be judged cautiously.

### 3.9. Swimming and Swarming Motilities

Distinct motile behaviors in soft agar systems were observed between the two strains. For strain 31D03, clear expansion zones were found to develop in both swimming (8.4 ± 2.6 mm, mean and range of the three replicates) and swarming (49.3 ± 2.7 mm) tests after 24 h of incubation, suggesting the relatively outperforming motile behavior of this strain. In contrast, neither swimming nor swarming motilities were observed for strain 30D07 within the 24 h incubation period (Supplementary Figure S1). No change was seen even when the incubation was prolonged to 48 h, in contrast with strain 31D03, which continuously expanded until entirely merging with the soft agar (Supplementary Figure S2). Interestingly, poorly developed swimming zones (6.1 ± 3.1 mm) of strain 30D07 were seen after one week of incubation at room temperature (Supplementary Figure S3), indicating the weak swimming ability exhibited by this strain under the experimental conditions.

### 3.10. Haemolytic Activity

β-haemolysis was displayed by strain 30D07 on Columbia Horse Blood agar at 37 °C, while γ-haemolysis (no haemolysis) was showed by strain 31D03 under the same condition (Supplementary Figure S4). Notably, the β-haemolytic activity of strain 30D07 on blood agar was not triggered at room temperature, while the growth of strain 31D03 remained non-haemolytic.

### 3.11. Bioluminescent Activity

A distinctive fluorescent effect was observed in the darkroom (i.e., no light source present) when strain 30D07 was cultured on Columbia Horse Blood agar at 37 °C for the haemolytic assay (Supplementary Figure S5). As with the presence of β-haemolysis, such fluorescent effect was not observed at room temperature. Strain 31D03 did not show any degree of bioluminescent activity at either 37 °C or at room temperature.

## 4. Discussion

Species-level identification of microorganisms is an essential prelude to various activities such as clinical intervention, outbreak investigation, food recalls, environmental remediation or enhanced monitoring. The practice is not always straightforward, especially when the organism belongs to a large and taxonomically complex group of organisms, including the *Vibrionaceae* [11,28] as discussed before for the Epsilonproteobacteria—a phylogenetically distinct group presenting similar identification challenges [37].

A previous strategy for identification of New Zealand shellfish isolates as *V. vulnificus* was based on colony morphology on two selective agars and a PCR assay, followed by the use of the commercial API20E kit [12]. Their identities were “confirmed” by this method, and were submitted for further study as *V. vulnificus*. Our current results clearly show that misidentifications can occur with this system, where the manufacturers database is used: the alternative database “BacDive” fails to provide a suggested taxon. Presumably, differences in database composition or assessment algorithm explain these results. Other isolates (41C02, 41B08, 34A09) identified by this method were confirmed to be *V. vulnificus* by their 97.9% homology of their ELS profile with the New Zealand reference strain (NZRM2506) (Table 1). Elastic light scatter (ELS) analysis examines the architecture of bacterial colonies using a laser and displays the forward-projected light scatter pattern, which has been shown to be a useful taxonomic “fingerprint” [29,34,38]. The method has been shown capable of distinguishing 11 *Vibrio* species (including *V. parahaemolyticus*, *V. vulnificus* and *V. campbellii*) and three *Aeromonas* species [34]. Our results support the ability of ELS to differentiate *Vibrio* species; however, identification of novel isolates requires a database of appropriate reference strains for effective comparison and matching. Unfortunately, our laboratory does not have an extensive collection of reference strains of each of the currently known 159 *Vibrio* species needed for full comparison.

Comparative analysis of 16S rRNA gene sequences is an extensively used molecular approach for bacterial identification [39]. However, the limitations of the tool are well established, since high (>95%) similarity values can be found between genetically separate species, including *Vibrio* [7,40,41,42]. This was the case here, with matches for both strains being found with up to 59 distinct *Vibrio* species, making for a sound assessment as to the genus identity of the isolates, but having insufficient granularity for effective species identification. *Allocatenococcus thiocycli* (formerly *Catenococcus thiocycli*), despite having a different genus assignation, is highly related to other *Vibrio* species [43,44]) and similarly could not be confidently distinguished from our strains with this method.

The WGS pipeline described here was found to be highly effective for identifying *Vibrio*. Other researchers have proposed that isDDH of WGS represents a “gold standard” for *Vibrio* species identification, but raised questions concerning the speed, cost and complexity of analysis [7,8,11]. Our experience addresses these questions positively. Our pipeline allows for complete sequences to be readily obtained using the relatively cost-effective Minion platform. This is no trivial matter, given that *Vibrio* species possess two chromosomes with a combined genome size exceeding 5 Mb, and often with plasmids (as seen here in strain 30D07). Furthermore, we believe that the surfeit of sequence data >30 kb produced (>140× oversampling) was an important factor underlying the efficiency of assembly, as suggested before [45]. Presently, faster pipelines for WGS of bacteria yield data better suited for surveillance purposes [46].

The ease by which our sequence data were assembled expedited their analysis for species identification using the online GGDC tool, which replicates the practice of DNA-DNA hybridisation that remains the “gold standard” for bacterial species definition [35]. GGDC can compare up to 75 genomes at a time without the need of downloading the reference genomes, provided they are publicly available [27]. This feature expedited the comparison of the genomes for our strains with those all other known *Vibrio* species. Our results clearly identify strain 30D03 as *V. campbellii*; to our knowledge this has not been reported in New Zealand bivalve shellfish before. *Vibrio campbellii* and *V. campbelli*-like species have previously been reported as causing vibriosis in New Zealand hatchery-reared fish [47] and pustule disease in abalone [48] while *V. rotiferianus* has not previously been reported in New Zealand. It is claimed that *V. campbellii* has been frequently misidentified as its close phylogenetic relative *V. harveyi*; nevertheless, *V. campbellii* is now confirmed as a predominant pathogen of shrimp in some regions [4]. Since *V. harveyi* is well established as a pathogen causing various diseases in a range of aquaculturally important species [6], and also in humans [49], the possibility that some of these conditions may also be caused by *V. campbellii* cannot be excluded. This warrants further consideration.

The GGDC results for strain 31D03 identified a close relationship to *V. rotiferianus*, an established pathogen for coral [5], black rockfish, rainbow trout and brine shrimp [2]. Such results were further supported by ANIb and ANIm analyses (Figure 5). Two of the three formulae included in the GGDC system predicted genome homology results exceeding the 70% threshold value for species assignation, whereas Formula 2 values fell short. The GGDC developers recommend the use of Formula 2 where such discrepancies are observed (cf. https://ggdc.dsmz.de/faq.php#qggdc6; accessed on 27 June 2025). However, *Vibrio* species differ from the majority of other bacteria in possessing two chromosomes, rather than one. Furthermore, for other taxonomically complex classes, Formula 3 appears to more accurately reflect established taxonomic relationships, and on this basis has been recommended for describing genomic relatedness [50]. Given that GGDC-based isDDH estimates model the laboratory-based equivalent, these results suggest that further work to understand the nature of these discrepancies may be warranted. Nonetheless, a genomic similarity of strain 31D03 to *V. rotiferianus* was the only relationship indicated in our analyses. Given, also, the phenotypic differences observed between *V. rotiferianus* [51] and strain 31D03—namely in its failure to ferment arabinose and melibiose, and produce urease, gelatinase, ornithine decarboxylase and tryptophan deaminase—we suggest a preliminary designation to 31D03 as ‘*V. novazealandii*’, pending the identification of additional isolates to make for a satisfactory formal taxonomic description. Importantly, our strain bears virulence genes similar to one proven to cause disease in black rockfish [2], as well as genes conferring resistance to several antimicrobial agents, clearly indicating pathogenic potential.

*V. campbellii* is a widely distributed aquacultural pathogen involved in diseases of molluscs, fish and shrimp, and its pathogenicity has been correlated with bioluminescence [52]. There is a lack of research evidence for its pathogenicity to humans and other mammals, although consuming contaminated seafood could clearly result in the risk of *V. campbellii* entering human and mammal food chains. Motility assays revealed the active swimming and swarming behaviors of strain 31D03, which could reflect the sound adaptation of this strain to the marine environment. Strain 30D07 barely showed any vibrant motilities in either 0.3% or 0.6% soft agar systems and its β-haemolysis and bioluminescent effect was displayed only at 37 °C but not at the lower room temperature. This temperature effect could suggest haemolytic activity of this strain to homeothermic animals rather than to poikilothermic aquatic animals, and hence, the possible pathogenic potential of this *V. campbellii* strain for humans. Clearly misidentifications potentially lead to underreporting in both human and aquacultural incidents, which obstruct pathogenesis and epidemiological investigation of foodborne vibriosis in humans. Furthermore, *Vibrio* is a genus with highly active horizontal gene-transfer events, so none of the emerging species or strains should be assumed to be non-pathogenic to humans. Certain genes that linked to infectious pathways of multiple bacterial pathogens, including *V. cholerae*, were predicted in the genomes of both the strains by KEGG functional annotation. Moreover, a considerable number of genes in the genomes of both strain 30D07 and 31D03 still remain hypothetical or functionally mysterious. The *V. vulnificus*-like API 20E profile and the 37 °C-triggered β-haemolysis and fluorescent effect, which were determined on *V. campbellii* strain 30D07 in this study, may provide certain phenotypic clues for tracking the pathogenic potential of this species to humans.

In conclusion, we describe, for the first time, *Vibrio* species present in the New Zealand marine environment that have been associated with diseases of aquaculturally important species in other jurisdictions. Since seafood exports achieved export revenues exceeding $NZ2 billion in 2024 (https://www.statista.com/statistics/1101037/new-zealand-export-revenue-from-seafood/; accessed on 19 September 2025), with rising ocean temperatures said to be influencing the prevalence of related species *V. parahaemolyticus* and *V. vulnificus* [12,53], these findings may have implications for this key economic area. Surveys for *Vibrio* species in aquacultural products in New Zealand are sporadic, thus the prevalence of the taxa described in this report cannot be determined presently, and perhaps should be considered in the future.

Our study also highlights the challenges faced when striving to identify bacterial isolates that belong to taxonomically complex groups. Of the methods applied, one (API20E) resulted in clear misidentification, or no identification when a different database was used. The widely used molecular approach of 16S rRNA gene sequence analysis fared little better, and while ELS showed promise, a significant upscaling of a reference database is required. Our WGS pipeline yielded high quality data quickly and efficiently, and comparisons with existing sequences using a widely accessible online tool readily achieved identification, making for an effective strategy for identifying isolates of taxonomically challenging groups. For clinically and industrially important organisms such as *Vibrio* species, this is a clear advantage.

## Figures and Tables

**Table 1:** Coefficient of Variance (%) matrix for Elastic Light Scatter profile comparisons between novel isolates 30D07 (*Vibrio campbellii*), 31D03 (*V. rotiferianus*-like), and type and reference strains of *V. parahaemolyticus* (30C10, 31C03, 32J10, 39D02, C11) and *V. vulnificus* (NZRM2506, 41C02, 41B08, 34A09). Homologous comparison values should exceed 90.0%. The number of ELS images used for each taxon in the analysis is presented in the format *n* = X in the table.

	30D07 (*n* = 50)	31D03 (*n* = 30)	*V. parahaemolyticus* (*n* = 80)	*V. vulnificus* (*n* = 90)
30D07	90.2	0	7.6	2.2
31D03	0	91	9	0
*V. parahaemolyticus*	0.3	0.3	98.7	0.7
*V. vulnificus*	0.2	0	1.9	97.9

**Table 2:** Summary of antimicrobial resistance (AMR) genes identified using the Comprehensive Antibiotic Resistance Database (CARD) [26]. ARO, Antibiotic Resistance Ontology; AST, Antibiotic Susceptibility Testing; NM, no match.

ARO Term	Detection Criteria	AMR Gene Family	Drug Class	Resistance Mechanism	AST Source	% Identity of Matching Region	
						30D07	31D03
*CRP*	protein homolog model	resistance-nodulation-cell division (RND) antibiotic efflux pump	macrolide antibiotic, fluoroquinolone antibiotic, penicillin beta-lactam	antibiotic efflux		95.24	95.24
*adeF*	protein homolog model	resistance-nodulation-cell division (RND) antibiotic efflux pump	fluoroquinolone antibiotic, tetracycline antibiotic	antibiotic efflux		NM	43.06
Escherichia coli *parE*	protein variant model	fluoroquinolone resistant *parE*	fluoroquinolone antibiotic	antibiotic target alteration	Curated-R	NM	78.66
Haemophilus influenzae *PBP3*	protein variant model	Penicillin-binding protein mutations conferring resistance to beta-lactam antibiotics	cephalosporin, penicillin beta-lactam	antibiotic target alteration	Curated-R	45.93	NM
*TxR*	protein homolog model	ATP-binding cassette (ABC) antibiotic efflux pump	tetracycline antibiotic	antibiotic efflux		98.43	69.59
*FosA8*	protein homolog model	fosfomycin thiol transferase	phosphonic acid antibiotic	antibiotic inactivation		50.75	NM
*qacG*	protein homolog model	small multidrug resistance (SMR) antibiotic efflux pump	disinfecting agents and antiseptics	antibiotic efflux		34.65	36.63
*vanT* in *vanG* cluster	protein homolog model	glycopeptide resistance gene cluster, *vanT*	glycopeptide antibiotic	antibiotic target alteration		33.06	33.06

**Table 3:** Antimicrobial resistance of *Vibrio* strains 30D07 and 31D03. Values are mean diameters observed ± standard deviation (*n* = 3).

Category	Antimicrobial Agents		30D07		31D03	
			Diameter of Inhibition Zone (mm)	Sensitivity	Diameter of Inhibition Zone (mm)	Sensitivity
β-lactam	Ampicillin	2 µg	0.0 ± 0.0	R *	0 ± 0.0	R *
		10 µg	0.0 ± 0.0	R *	0 ± 0.0	R *
	Penicillin G	5 U	0.0 ± 0.0	R *	0 ± 0.0	R *
Aminoglycosides	Gentamicin	10 µg	11.3 ± 0.2	R ^1^	10.4 ± 0.1	R ^1^
	Kanamycin	30 µg	12.8 ± 0.1	R ^2^	10.2 ± 0.3	R ^2^
Carbapenems	Meropenem	10 µg	16.8 ± 0.1	R ^1^	18.8 ± 0.4	R ^1^
Cephalosporins	Cefoxitin	30 µg	0.0 ± 0.0	R *	0.0 ± 0.0	R *
	Cephalothin	30 µg	9.5 ± 0.3	R ^3^	11.9 ± 0.2	R ^3^
Fluoroquinolones	Ciprofloxacin	5 µg	16.2 ± 0.1	R ^1^	19.0 ± 0.2	R ^1^
Macrolides	Azithromycin	15 µg	12.9 ± 0.4	R ^4^	11.0 ± 0.3	R ^4^
	Erythromycin	15 µg	11.6 ± 0.3	R ^5^	11.1 ± 0.2	R ^5^
Phenicols	Chloramphenicol	30 µg	22.0 ± 0.1	R ^1^	23.0 ± 0.3	R ^1^
Quinolones	Nalidixic acid	30 µg	23.2 ± 0.2	R ^1^	25.6 ± 0.7	R ^1^
Tetracyclines	Tetracycline	30 µg	20.3 ± 1.0	R ^1^	24.2 ± 1.4	S ^1^

R: resistant; S: sensitive. * No visible inhibition zone was observed. ^1^ As per recommendations to CLSI for *V. cholerae* from Smith et al. (2023) [32]. ^2^ As per recommendations for Enterobacterales (excluding *Salmonella* and *Shigella*), CLSI M100, 35th edition. ^3^ As per quality control ranges for sensitivity using strain ATCC 25922 (*Escherichia coli*), CLSI M100, 35th edition. ^4^ As per recommendations for *Salmonella* and *Shigella*, CLSI M100, 35th edition. ^5^ As per quality control ranges for sensitivity using strain ATCC 25923 (Staphylococcus aureus), CLSI M100, 35th edition.

## Data Availability

The genome and plasmid sequences described in this article are available from GenBank with the accession numbers CP192309–CP192311 for Chromosome I, Chromosome II and the plasmid of 30D07 (*V. campbellii*), respectively, and CP194563 and CP194564 for Chromosome I and Chromosome II of 31D03 (*V. rotifeiranus*-like). The MinION read data can be accessed at doi.org/10.25400/lincolnuninz.33011450.

## Supplementary Materials

The following supporting information can be downloaded at: https://www.mdpi.com/article/10.3390/microorganisms14081658/s1.
