# Supplementary material for: Addressing the Challenges of Identifying Vibrio Species; Genomic and Phenotypic Analyses of Misidentified Vibrio vulnificus Strains Reveal Vibrio campbellii and a Potentially Novel V. rotiferianus-like Species in New Zealand Oysters
Source: Microorganisms. 2026 Jul 29;14(8):1658. doi: 10.3390/microorganisms14081658 (PMC13515124; doi:10.3390/microorganisms14081658)

Supplementary Table S1. 16S rRNA gene sequence comparisons of strain 30D07 with closely related taxa in NCBI using BLAST.

23/05/2025, 16:26 NCBI Blast:30D07contig_1_-_16S_rRNA1551 Vibrio strain

Description	Scientific Name	Max Score	Total Score	Query Cover	E value	Per. Ident	Acc. Len	Accession
Vibrio alginolyticus strain NBRC 15630 16S ribosomal RNA, partial sequence	Vibrio alginolyticus	2778	2778	99%	0.0	99.22%	1541	NR_121709.1
Vibrio alginolyticus strain NBRC 15630 16S ribosomal RNA, partial sequence	Vibrio alginolyticus	2772	2772	99%	0.0	99.16%	1541	NR_122060.1
Vibrio alginolyticus strain NBRC 15630 16S ribosomal RNA, partial sequence	Vibrio alginolyticus	2756	2756	99%	0.0	98.96%	1541	NR_122050.1
Vibrio alginolyticus strain NBRC 15630 16S ribosomal RNA, partial sequence	Vibrio alginolyticus	2745	2745	99%	0.0	98.83%	1541	NR_122059.1
Vibrio campbellii strain NBRC 15631 16S ribosomal RNA, partial sequence	Vibrio campbellii	2686	2686	95%	0.0	99.39%	1478	NR_113782.1
Vibrio parahaemolyticus strain ATCC 17802 16S ribosomal RNA, partial sequence	Vibrio parahaemolyticus	2684	2684	97%	0.0	98.74%	1514	NR_114630.1
Vibrio rotiferianus CAIM 577 = LMG 21460 16S ribosomal RNA, partial sequence	Vibrio rotiferianus CAIM 577 = LMG 21460	2682	2682	95%	0.0	99.59%	1471	NR_118091.1
Vibrio rotiferianus CAIM 577 = LMG 21460 16S ribosomal RNA, partial sequence	Vibrio rotiferianus CAIM 577 = LMG 21460	2676	2676	95%	0.0	99.59%	1468	NR_042081.1
Vibrio campbellii CAIM 519 = NBRC 15631 = ATCC 25920 16S ribosomal RNA, partial sequence	Vibrio campbellii CAIM 519 = NBRC 15631 = ATCC 25920	2676	2676	94%	0.0	99.59%	1467	NR_119050.1
Vibrio parahaemolyticus strain ATCC 17802 16S ribosomal RNA, partial sequence	Vibrio parahaemolyticus	2673	2673	97%	0.0	98.61%	1514	NR_114632.1
Vibrio parahaemolyticus strain ATCC 17802 16S ribosomal RNA, partial sequence	Vibrio parahaemolyticus	2673	2673	97%	0.0	98.61%	1514	NR_041838.1
Vibrio alginolyticus strain NBRC 15630 16S ribosomal RNA, partial sequence	Vibrio alginolyticus	2669	2669	95%	0.0	99.26%	1478	NR_113781.1
Vibrio parahaemolyticus strain ATCC 17802 16S ribosomal RNA, partial sequence	Vibrio parahaemolyticus	2667	2667	97%	0.0	98.55%	1514	NR_114631.1
Vibrio ouci strain BEI176 16S ribosomal RNA, partial sequence	Vibrio ouci	2656	2656	97%	0.0	98.41%	1529	NR_179908.1
Vibrio alginolyticus strain ATCC 17749 16S ribosomal RNA, partial sequence	Vibrio alginolyticus	2654	2654	95%	0.0	99.25%	1471	NR_117895.1
Vibrio harveyi strain ATCC 14126 16S ribosomal RNA, partial sequence	Vibrio harveyi	2651	2651	96%	0.0	98.79%	1488	NR_119054.1
Vibrio natriegens NBRC 15636 = ATCC	Vibrio natriegens NBRC 15636 =	2645	2645	95%	0.0	98.99%	1478	NR_113786.1

Description	Scientific Name	Max Score	Total Score	Query Cover	E value	Per. Ident	Acc. Len	Accession
14048 = DSM 759 16S ribosomal RNA, partial sequence	ATCC 14048 = DSM 759							
Vibrio natriegens NBRC 15636 = ATCC 14048 = DSM 759 16S ribosomal RNA, partial sequence	Vibrio natriegens NBRC 15636 = ATCC 14048 = DSM 759	2643	2643	95%	0.0	99.12%	1471	NR_117890.1
Vibrio harveyi strain NBRC 15634 16S ribosomal RNA, partial sequence	Vibrio harveyi	2634	2634	95%	0.0	98.85%	1477	NR_113784.1
Vibrio parahaemolyticus strain ATCC 17802 16S ribosomal RNA, partial sequence	Vibrio parahaemolyticus	2632	2632	95%	0.0	98.91%	1471	NR_119058.1
Vibrio parahaemolyticus strain NBRC 12711 16S ribosomal RNA, partial sequence	Vibrio parahaemolyticus	2630	2630	95%	0.0	98.78%	1478	NR_113604.1
Vibrio alginolyticus strain ATCC 17749 16S ribosomal RNA, partial sequence	Vibrio alginolyticus	2630	2630	95%	0.0	98.98%	1468	NR_119049.1
Vibrio natriegens NBRC 15636 = ATCC 14048 = DSM 759 16S ribosomal RNA, partial sequence	Vibrio natriegens NBRC 15636 = ATCC 14048 = DSM 759	2625	2625	94%	0.0	98.98%	1466	NR_026124.1
Vibrio azureus strain LC2-005 16S ribosomal RNA, partial sequence	Vibrio azureus	2623	2623	95%	0.0	98.78%	1475	NR_117997.1
Vibrio parahaemolyticus strain ATCC 17802 16S ribosomal RNA, partial sequence	Vibrio parahaemolyticus	2615	2615	95%	0.0	98.78%	1471	NR_117893.1
Vibrio crosai strain CAIM 1437 16S ribosomal RNA, partial sequence	Vibrio crosai	2612	2612	97%	0.0	97.94%	1507	NR_148264.1
Vibrio europaeus strain PP-638 16S ribosomal RNA, partial sequence	Vibrio europaeus	2610	2610	98%	0.0	97.51%	1528	NR_146051.1
Vibrio alginolyticus strain ATCC 17749 16S ribosomal RNA, partial sequence	Vibrio alginolyticus	2603	2603	96%	0.0	98.32%	1482	NR_044825.2
Vibrio pelagius strain ATCC 25916 16S ribosomal RNA, partial sequence	Vibrio pelagius	2601	2601	94%	0.0	98.57%	1464	NR_119059.1
Vibrio campbellii strain 40 16S ribosomal RNA, partial sequence	Vibrio campbellii	2599	2599	96%	0.0	98.26%	1484	NR_029222.1
Vibrio pelagius strain CECT 4202 16S ribosomal RNA, partial sequence	Vibrio pelagius	2591	2591	100%	0.0	96.96%	1544	NR_042043.1
Vibrio nitrifigilis strain NFV-1 16S ribosomal RNA, partial sequence	Vibrio nitrifigilis	2586	2586	100%	0.0	96.84%	1547	NR_181541.1
Vibrio ezurae strain HDS1-1 16S ribosomal RNA, partial sequence	Vibrio ezurae	2584	2584	97%	0.0	97.49%	1520	NR_025779.1
Vibrio fortis strain CAIM 629 16S	Vibrio fortis	2582	2582	97%	0.0	97.54%	1505	NR_025575.1

Description	Scientific Name	Max Score	Total Score	Query Cover	E value	Per. Ident	Acc. Len	Accession
ribosomal RNA, partial sequence								
Vibrio agarivorans strain 289 16S ribosomal RNA, partial sequence	Vibrio agarivorans	2577	2577	99%	0.0	96.89%	1542	NR_028946.1
Vibrio proteolyticus strain NBRC 13287 16S ribosomal RNA, partial sequence	Vibrio proteolyticus	2577	2577	95%	0.0	98.11%	1478	NR_113610.1
Vibrio azureus strain NBRC 104587 16S ribosomal RNA, partial sequence	Vibrio azureus	2577	2577	95%	0.0	98.17%	1477	NR_114268.1
Vibrio proteolyticus strain ATCC 15338 16S ribosomal RNA, partial sequence	Vibrio proteolyticus	2573	2573	94%	0.0	98.36%	1465	NR_026128.1
Vibrio proteolyticus strain LMG 3772 16S ribosomal RNA, partial sequence	Vibrio proteolyticus	2571	2571	95%	0.0	98.23%	1471	NR_118095.1
Vibrio alginolyticus strain ATCC 17749 16S ribosomal RNA, partial sequence	Vibrio alginolyticus	2567	2567	92%	0.0	99.16%	1458	NR_118258.1
Vibrio jasicida strain BFLP-10 16S ribosomal RNA, partial sequence	Vibrio jasicida	2567	2567	96%	0.0	97.91%	1484	NR_134829.1
Vibrio harveyi strain NCIMB1280 16S ribosomal RNA, partial sequence	Vibrio harveyi	2564	2564	93%	0.0	98.89%	1436	NR_043165.1
Vibrio aquaticus strain BEI207 16S ribosomal RNA, partial sequence	Vibrio aquaticus	2562	2562	97%	0.0	97.29%	1529	NR_179909.1
Vibrio maritimus strain R-40493 16S ribosomal RNA, partial sequence	Vibrio maritimus	2562	2562	97%	0.0	97.14%	1504	NR_117551.1
Vibrio tubiashii ATCC 19109 strain NBRC 15644 16S ribosomal RNA, partial sequence	Vibrio tubiashii ATCC 19109	2551	2551	95%	0.0	97.77%	1479	NR_113791.1
Vibrio furnissii strain 9119-82 16S ribosomal RNA, partial sequence	Vibrio furnissii	2551	2551	99%	0.0	96.44%	1546	NR_037067.1
Vibrio owensii CAIM 1854 = LMG 25443 strain DY05 16S ribosomal RNA, partial sequence	Vibrio owensii CAIM 1854 = LMG 25443	2547	2547	91%	0.0	99.01%	1421	NR_117424.1
Vibrio bivalvicida 16S ribosomal RNA, partial sequence	Vibrio bivalvicida	2543	2543	96%	0.0	97.52%	1489	NR_135208.1
Vibrio tubiashii ATCC 19109 16S ribosomal RNA, partial sequence	Vibrio tubiashii ATCC 19109	2543	2543	95%	0.0	97.89%	1471	NR_118093.1
Vibrio brasiliensis LMG 20546 16S ribosomal RNA, partial sequence	Vibrio brasiliensis LMG 20546	2542	2542	97%	0.0	97.01%	1504	NR_025477.1
Vibrio galatheae strain S2757 16S ribosomal RNA, partial sequence	Vibrio galatheae	2540	2540	93%	0.0	98.41%	1444	NR_147758.1
Vibrio japonicus strain Bio7-2 16S	Vibrio japonicus	2538	2538	95%	0.0	97.29%	1477	NR_149201.1

Description	Scientific Name	Max Score	Total Score	Query Cover	E value	Per. Ident	Acc. Len	Accession
ribosomal RNA, partial sequence								
Vibrio pelagius strain NBRC 15639 16S ribosomal RNA, partial sequence	Vibrio pelagius	2534	2534	95%	0.0	97.50%	1478	NR_113789.1
Vibrio hepatarius strain LMG 20362 16S ribosomal RNA, partial sequence	Vibrio hepatarius	2527	2527	95%	0.0	97.76%	1468	NR_025491.1
Vibrio proteolyticus strain ATCC 15338 16S ribosomal RNA, partial sequence	Vibrio proteolyticus	2523	2523	96%	0.0	97.18%	1482	NR_118927.1
Vibrio hangzhouensis strain cn83 16S ribosomal RNA, partial sequence	Vibrio hangzhouensis	2523	2523	95%	0.0	97.56%	1475	NR_044396.1
Vibrio mediterranei strain CECT 621 16S ribosomal RNA, partial sequence	Vibrio mediterranei	2521	2521	98%	0.0	96.71%	1517	NR_117681.1
Vibrio ziniensis strain ZWAL4003 16S ribosomal RNA, complete sequence	Vibrio ziniensis	2519	2519	100%	0.0	96.08%	1543	NR_181540.1
Vibrio hispanicus strain LMG 13240 16S ribosomal RNA, partial sequence	Vibrio hispanicus	2518	2518	98%	0.0	96.64%	1517	NR_042806.1
Vibrio nereis strain NBRC 15637 16S ribosomal RNA, partial sequence	Vibrio nereis	2516	2516	95%	0.0	97.30%	1478	NR_113787.1
Vibrio hispanicus strain LMG 13240 16S ribosomal RNA, partial sequence	Vibrio hispanicus	2516	2516	98%	0.0	96.64%	1516	NR_115180.1
Vibrio neocaledonicus strain NC470 16S ribosomal RNA, partial sequence	Vibrio neocaledonicus	2514	2514	91%	0.0	98.66%	1418	NR_118432.1
Vibrio vulnificus strain 324 16S ribosomal RNA, partial sequence	Vibrio vulnificus	2508	2508	99%	0.0	96.06%	1536	NR_036888.1
Vibrio fluvialis strain VL 5125 16S ribosomal RNA, partial sequence	Vibrio fluvialis	2508	2508	99%	0.0	96.05%	1537	NR_036790.1
Vibrio ezurae strain NBRC 102218 16S ribosomal RNA, partial sequence	Vibrio ezurae	2507	2507	95%	0.0	97.09%	1478	NR_114045.1
Vibrio ponticus strain 69 16S ribosomal RNA, partial sequence	Vibrio ponticus	2505	2505	98%	0.0	96.28%	1529	NR_029032.1
Vibrio nereis strain ATCC 25917 16S ribosomal RNA, partial sequence	Vibrio nereis	2505	2505	95%	0.0	97.42%	1471	NR_118092.1
Vibrio caribbeanicus ATCC BAA-2122 strain N384 16S ribosomal RNA, partial sequence	Vibrio caribbeanicus ATCC BAA-2122	2503	2503	95%	0.0	97.42%	1470	NR_108732.1
Vibrio mytili strain CECT 632 16S ribosomal RNA, partial sequence	Vibrio mytili	2501	2501	93%	0.0	97.99%	1441	NR_044911.1

Description	Scientific Name	Max Score	Total Score	Query Cover	E value	Per. Ident	Acc. Len	Accession
Vibrio taketomensis strain C4III282 16S ribosomal RNA, partial sequence	Vibrio taketomensis	2494	2494	97%	0.0	96.68%	1501	NR_179297.1
Vibrio brasiliensis LMG 20546 16S ribosomal RNA, partial sequence	Vibrio brasiliensis LMG 20546	2494	2494	95%	0.0	97.28%	1470	NR_117887.1
Allocatenococcus thiocycli strain TG 5-3 16S ribosomal RNA, partial sequence	Allocatenococcus thiocycli	2492	2492	99%	0.0	95.92%	1539	NR_104870.1
Vibrio chemaguriensis strain Iso1 16S ribosomal RNA, partial sequence	Vibrio chemaguriensis	2490	2490	93%	0.0	97.86%	1454	NR_179846.1
Vibrio parahaemolyticus strain ATCC 17802 16S ribosomal RNA, partial sequence	Vibrio parahaemolyticus	2488	2488	96%	0.0	96.91%	1481	NR_118928.1
Vibrio orientalis strain NBRC 15638 16S ribosomal RNA, partial sequence	Vibrio orientalis	2484	2484	95%	0.0	97.03%	1478	NR_113788.1
Vibrio mediterranei strain AK1 16S ribosomal RNA, partial sequence	Vibrio mediterranei	2484	2484	97%	0.0	96.54%	1501	NR_114417.1
Vibrio xuii strain R-15052 16S ribosomal RNA, partial sequence	Vibrio xuii	2479	2479	92%	0.0	97.84%	1435	NR_025478.1
Vibrio alfacsensis strain CAIM 1831 16S ribosomal RNA, partial sequence	Vibrio alfacsensis	2479	2479	96%	0.0	96.60%	1497	NR_118129.1
Vibrio jasicida CAIM 1864 = LMG 25398 strain TCFB 0772 16S ribosomal RNA, partial sequence	Vibrio jasicida CAIM 1864 = LMG 25398	2479	2479	90%	0.0	98.64%	1400	NR_113182.1
Vibrio sinaloensis strain CAIM 797 16S ribosomal RNA, partial sequence	Vibrio sinaloensis	2475	2475	96%	0.0	96.65%	1488	NR_043858.1
Vibrio tubiashii strain Milford 74 16S ribosomal RNA, partial sequence	Vibrio tubiashii	2473	2473	92%	0.0	97.64%	1436	NR_026129.1
Vibrio lentus strain 40M4T 16S ribosomal RNA, partial sequence	Vibrio lentus	2471	2471	100%	0.0	95.48%	1559	NR_028926.1
Vibrio tapetis subsp. britannicus strain HH6087 16S ribosomal RNA, partial sequence	Vibrio tapetis subsp. britannicus	2471	2471	99%	0.0	95.73%	1533	NR_132307.1
Vibrio halioticoli strain NBRC 102217 16S ribosomal RNA, partial sequence	Vibrio halioticoli	2471	2471	95%	0.0	96.75%	1478	NR_114044.1
Vibrio orientalis CIP 102891 = ATCC 33934 16S ribosomal RNA, partial sequence	Vibrio orientalis CIP 102891 = ATCC 33934	2471	2471	95%	0.0	97.01%	1471	NR_118094.1
Vibrio ishigakensis strain C1 16S ribosomal RNA, complete sequence	Vibrio ishigakensis	2470	2470	94%	0.0	96.99%	1460	NR_156028.1

Description	Scientific Name	Max Score	Total Score	Query Cover	E value	Per. Ident	Acc. Len	Accession
Vibrio hyugaensis strain 090810a 16S ribosomal RNA, partial sequence	Vibrio hyugaensis	2470	2470	89%	0.0	98.63%	1390	NR_145569.1
Vibrio nigripulchritudo ATCC 27043 16S ribosomal RNA, partial sequence	Vibrio nigripulchritudo ATCC 27043	2470	2470	95%	0.0	97.01%	1470	NR_117897.1
Vibrio xiamenensis strain G21 16S ribosomal RNA, partial sequence	Vibrio xiamenensis	2468	2468	95%	0.0	96.88%	1475	NR_108603.1
Vibrio agarilyticus strain SM6 16S ribosomal RNA, partial sequence	Vibrio agarilyticus	2466	2466	97%	0.0	96.23%	1513	NR_180856.1
Vibrio tapetis strain CECT 4600 16S ribosomal RNA, partial sequence	Vibrio tapetis	2462	2462	99%	0.0	95.66%	1531	NR_117643.1
Vibrio orientalis strain 717 16S ribosomal RNA, partial sequence	Vibrio orientalis	2460	2460	94%	0.0	96.93%	1466	NR_026127.1
Vibrio artabrorum strain VB 11.8 16S ribosomal RNA, partial sequence	Vibrio artabrorum	2460	2460	99%	0.0	95.48%	1544	NR_116068.1
Vibrio chagasii strain LMG 21353 16S ribosomal RNA, partial sequence	Vibrio chagasii	2460	2460	95%	0.0	96.87%	1471	NR_117891.1
Vibrio penaeicida strain DSM 14398 16S ribosomal RNA, partial sequence	Vibrio penaeicida	2457	2457	98%	0.0	96.11%	1504	NR_042121.1
Vibrio mediterranei strain NBRC 15635 16S ribosomal RNA, partial sequence	Vibrio mediterranei	2457	2457	95%	0.0	96.62%	1478	NR_113785.1
Vibrio atlanticus strain VB 11.11 16S ribosomal RNA, partial sequence	Vibrio atlanticus	2457	2457	99%	0.0	95.58%	1536	NR_116067.1
Vibrio mediterranei strain ATCC 43341 16S ribosomal RNA, partial sequence	Vibrio mediterranei	2455	2455	95%	0.0	96.81%	1471	NR_117896.1
Vibrio coralliilyticus strain ATCC BAA-450 16S ribosomal RNA, partial sequence	Vibrio coralliilyticus	2455	2455	95%	0.0	96.81%	1471	NR_117892.1
Vibrio thalassae strain CECT 8203 16S ribosomal RNA, partial sequence	Vibrio thalassae	2449	2449	94%	0.0	96.87%	1467	NR_134228.1

Supplementary Table S2. 16S rRNA gene sequence comparisons of strain 31D03 with closely related taxa in NCBI using BLAST.

Description	Scientific Name	Max Score	Total Score	Query Cover	E value	Per. Ident	Acc. Len	Accession
Vibrio alginolyticus strain NBRC 15630 16S ribosomal RNA, partial sequence	Vibrio alginolyticus	2747	2747	99%	0.0	98.83%	1541	NR_122050.1
Vibrio alginolyticus strain NBRC 15630 16S ribosomal RNA, partial sequence	Vibrio alginolyticus	2741	2741	99%	0.0	98.77%	1541	NR_122059.1
Vibrio alginolyticus strain NBRC 15630 16S ribosomal RNA, partial sequence	Vibrio alginolyticus	2736	2736	99%	0.0	98.70%	1541	NR_121709.1
Vibrio alginolyticus strain NBRC 15630 16S ribosomal RNA, partial sequence	Vibrio alginolyticus	2730	2730	99%	0.0	98.64%	1541	NR_122060.1
Vibrio parahaemolyticus strain ATCC 17802 16S ribosomal RNA, partial sequence	Vibrio parahaemolyticus	2691	2691	97%	0.0	98.81%	1514	NR_114630.1
Vibrio parahaemolyticus strain ATCC 17802 16S ribosomal RNA, partial sequence	Vibrio parahaemolyticus	2691	2691	97%	0.0	98.81%	1514	NR_041838.1
Vibrio parahaemolyticus strain ATCC 17802 16S ribosomal RNA, partial sequence	Vibrio parahaemolyticus	2686	2686	97%	0.0	98.74%	1514	NR_114631.1
Vibrio parahaemolyticus strain ATCC 17802 16S ribosomal RNA, partial sequence	Vibrio parahaemolyticus	2680	2680	97%	0.0	98.68%	1514	NR_114632.1
Vibrio rotiferianus CAIM 577 = LMG 21460 16S ribosomal RNA, partial sequence	Vibrio rotiferianus CAIM 577 = LMG 21460	2662	2662	95%	0.0	99.32%	1471	NR_118091.1
Vibrio campbellii strain NBRC 15631 16S ribosomal RNA, partial sequence	Vibrio campbellii	2658	2658	95%	0.0	99.05%	1478	NR_113782.1
Vibrio rotiferianus CAIM 577 = LMG 21460 16S ribosomal RNA, partial sequence	Vibrio rotiferianus CAIM 577 = LMG 21460	2656	2656	95%	0.0	99.32%	1468	NR_042081.1
Vibrio campbellii CAIM 519 = NBRC 15631 = ATCC 25920 16S ribosomal RNA, partial sequence	Vibrio campbellii CAIM 519 = NBRC 15631 = ATCC 25920	2645	2645	94%	0.0	99.18%	1467	NR_119050.1
Vibrio ouci strain BEI176 16S ribosomal RNA, partial sequence	Vibrio ouci	2641	2641	97%	0.0	98.22%	1529	NR_179908.1
Vibrio parahaemolyticus strain NBRC 12711 16S ribosomal RNA, partial sequence	Vibrio parahaemolyticus	2638	2638	95%	0.0	98.85%	1478	NR_113604.1
Vibrio alginolyticus strain NBRC 15630 16S ribosomal RNA, partial sequence	Vibrio alginolyticus	2632	2632	95%	0.0	98.78%	1478	NR_113781.1
Vibrio azureus strain LC2-005 16S ribosomal RNA, partial sequence	Vibrio azureus	2630	2630	95%	0.0	98.85%	1475	NR_117997.1

Description	Scientific Name	Max Score	Total Score	Query Cover	E value	Per. Ident	Acc. Len	Accession
Vibrio harveyi strain ATCC 14126 16S ribosomal RNA, partial sequence	Vibrio harveyi	2625	2625	96%	0.0	98.46%	1488	NR_119054.1
Vibrio parahaemolyticus strain ATCC 17802 16S ribosomal RNA, partial sequence	Vibrio parahaemolyticus	2623	2623	95%	0.0	98.85%	1471	NR_117893.1
Vibrio harveyi strain NBRC 15634 16S ribosomal RNA, partial sequence	Vibrio harveyi	2619	2619	95%	0.0	98.65%	1477	NR_113784.1
Vibrio alginolyticus strain ATCC 17749 16S ribosomal RNA, partial sequence	Vibrio alginolyticus	2612	2612	95%	0.0	98.71%	1471	NR_117895.1
Vibrio parahaemolyticus strain ATCC 17802 16S ribosomal RNA, partial sequence	Vibrio parahaemolyticus	2606	2606	95%	0.0	98.57%	1471	NR_119058.1
Vibrio alginolyticus strain ATCC 17749 16S ribosomal RNA, partial sequence	Vibrio alginolyticus	2604	2604	95%	0.0	98.64%	1468	NR_119049.1
Vibrio crosai strain CAIM 1437 16S ribosomal RNA, partial sequence	Vibrio crosai	2603	2603	97%	0.0	97.81%	1507	NR_148264.1
Vibrio natriegens NBRC 15636 = ATCC 14048 = DSM 759 16S ribosomal RNA, partial sequence	Vibrio natriegens NBRC 15636 = ATCC 14048 = DSM 759	2603	2603	95%	0.0	98.45%	1478	NR_113786.1
Vibrio europaeus strain PP-638 16S ribosomal RNA, partial sequence	Vibrio europaeus	2601	2601	98%	0.0	97.39%	1528	NR_146051.1
Vibrio natriegens NBRC 15636 = ATCC 14048 = DSM 759 16S ribosomal RNA, partial sequence	Vibrio natriegens NBRC 15636 = ATCC 14048 = DSM 759	2601	2601	95%	0.0	98.57%	1471	NR_117890.1
Vibrio pelagius strain CECT 4202 16S ribosomal RNA, partial sequence	Vibrio pelagius	2593	2593	100%	0.0	96.97%	1544	NR_042043.1
Vibrio ezurae strain HDS1-1 16S ribosomal RNA, partial sequence	Vibrio ezurae	2586	2586	97%	0.0	97.50%	1520	NR_025779.1
Vibrio jasicida strain BFLP-10 16S ribosomal RNA, partial sequence	Vibrio jasicida	2586	2586	96%	0.0	98.12%	1484	NR_134829.1
Vibrio azureus strain NBRC 104587 16S ribosomal RNA, partial sequence	Vibrio azureus	2584	2584	95%	0.0	98.24%	1477	NR_114268.1
Vibrio nitrifigilis strain NFV-1 16S ribosomal RNA, partial sequence	Vibrio nitrifigilis	2582	2582	100%	0.0	96.78%	1547	NR_181541.1
Vibrio natriegens NBRC 15636 = ATCC 14048 = DSM 759 16S ribosomal RNA, partial sequence	Vibrio natriegens NBRC 15636 = ATCC 14048 = DSM 759	2582	2582	94%	0.0	98.43%	1466	NR_026124.1
Vibrio fortis strain CAIM 629 16S ribosomal RNA, partial sequence	Vibrio fortis	2579	2579	97%	0.0	97.48%	1505	NR_025575.1

Description	Scientific Name	Max Score	Total Score	Query Cover	E value	Per. Ident	Acc. Len	Accession
Vibrio aquaticus strain BEI207 16S ribosomal RNA, partial sequence	Vibrio aquaticus	2575	2575	97%	0.0	97.42%	1529	NR_179909.1
Vibrio furnissii strain 9119-82 16S ribosomal RNA, partial sequence	Vibrio furnissii	2575	2575	99%	0.0	96.71%	1546	NR_037067.1
Vibrio bivalvicida 16S ribosomal RNA, partial sequence	Vibrio bivalvicida	2573	2573	96%	0.0	97.85%	1489	NR_135208.1
Vibrio hepatarius strain LMG 20362 16S ribosomal RNA, partial sequence	Vibrio hepatarius	2571	2571	95%	0.0	98.23%	1468	NR_025491.1
Vibrio alginolyticus strain ATCC 17749 16S ribosomal RNA, partial sequence	Vibrio alginolyticus	2571	2571	96%	0.0	97.92%	1482	NR_044825.2
Vibrio brasiliensis LMG 20546 16S ribosomal RNA, partial sequence	Vibrio brasiliensis LMG 20546	2569	2569	97%	0.0	97.41%	1504	NR_025477.1
Vibrio pelagius strain ATCC 25916 16S ribosomal RNA, partial sequence	Vibrio pelagius	2569	2569	94%	0.0	98.16%	1464	NR_119059.1
Vibrio campbellii strain 40 16S ribosomal RNA, partial sequence	Vibrio campbellii	2567	2567	96%	0.0	97.86%	1484	NR_029222.1
Vibrio ponticus strain 69 16S ribosomal RNA, partial sequence	Vibrio ponticus	2567	2567	98%	0.0	97.00%	1529	NR_029032.1
Vibrio maritimus strain R-40493 16S ribosomal RNA, partial sequence	Vibrio maritimus	2564	2564	97%	0.0	97.21%	1504	NR_117551.1
Vibrio agarivorans strain 289 16S ribosomal RNA, partial sequence	Vibrio agarivorans	2562	2562	99%	0.0	96.70%	1542	NR_028946.1
Vibrio tubiashii ATCC 19109 strain NBRC 15644 16S ribosomal RNA, partial sequence	Vibrio tubiashii ATCC 19109	2558	2558	95%	0.0	97.84%	1479	NR_113791.1
Vibrio taketomensis strain C4III282 16S ribosomal RNA, partial sequence	Vibrio taketomensis	2556	2556	97%	0.0	97.41%	1501	NR_179297.1
Vibrio nereis strain NBRC 15637 16S ribosomal RNA, partial sequence	Vibrio nereis	2556	2556	95%	0.0	97.77%	1478	NR_113787.1
Vibrio mediterranei strain CECT 621 16S ribosomal RNA, partial sequence	Vibrio mediterranei	2556	2556	98%	0.0	97.10%	1517	NR_117681.1
Vibrio proteolyticus strain NBRC 13287 16S ribosomal RNA, partial sequence	Vibrio proteolyticus	2551	2551	95%	0.0	97.77%	1478	NR_113610.1
Vibrio tubiashii ATCC 19109 16S ribosomal RNA, partial sequence	Vibrio tubiashii ATCC 19109	2551	2551	95%	0.0	97.96%	1471	NR_118093.1
Vibrio alfacsensis strain CAIM 1831 16S ribosomal RNA, partial sequence	Vibrio alfacsensis	2547	2547	96%	0.0	97.40%	1497	NR_118129.1

Description	Scientific Name	Max Score	Total Score	Query Cover	E value	Per. Ident	Acc. Len	Accession
Vibrio nereis strain ATCC 25917 16S ribosomal RNA, partial sequence	Vibrio nereis	2545	2545	95%	0.0	97.89%	1471	NR_118092.1
Vibrio xuii strain R-15052 16S ribosomal RNA, partial sequence	Vibrio xuii	2542	2542	92%	0.0	98.61%	1435	NR_025478.1
Vibrio proteolyticus strain ATCC 15338 16S ribosomal RNA, partial sequence	Vibrio proteolyticus	2542	2542	94%	0.0	97.96%	1465	NR_026128.1
Vibrio proteolyticus strain LMG 3772 16S ribosomal RNA, partial sequence	Vibrio proteolyticus	2540	2540	95%	0.0	97.83%	1471	NR_118095.1
Allocatenococcus thiocycli strain TG 5-3 16S ribosomal RNA, partial sequence	Allocatenococcus thiocycli	2538	2538	99%	0.0	96.43%	1539	NR_104870.1
Vibrio harveyi strain NCIMB1280 16S ribosomal RNA, partial sequence	Vibrio harveyi	2538	2538	93%	0.0	98.54%	1436	NR_043165.1
Vibrio owensii CAIM 1854 = LMG 25443 strain DY05 16S ribosomal RNA, partial sequence	Vibrio owensii CAIM 1854 = LMG 25443	2532	2532	92%	0.0	98.81%	1421	NR_117424.1
Vibrio galatheae strain S2757 16S ribosomal RNA, partial sequence	Vibrio galatheae	2531	2531	93%	0.0	98.27%	1444	NR_147758.1
Vibrio pelagius strain NBRC 15639 16S ribosomal RNA, partial sequence	Vibrio pelagius	2531	2531	95%	0.0	97.43%	1478	NR_113789.1
Vibrio brasiliensis LMG 20546 16S ribosomal RNA, partial sequence	Vibrio brasiliensis LMG 20546	2527	2527	95%	0.0	97.69%	1470	NR_117887.1
Vibrio alginolyticus strain ATCC 17749 16S ribosomal RNA, partial sequence	Vibrio alginolyticus	2525	2525	92%	0.0	98.60%	1458	NR_118258.1
Vibrio mediterranei strain AK1 16S ribosomal RNA, partial sequence	Vibrio mediterranei	2525	2525	97%	0.0	97.01%	1501	NR_114417.1
Vibrio japonicus strain Bio7-2 16S ribosomal RNA, partial sequence	Vibrio japonicus	2523	2523	95%	0.0	97.23%	1477	NR_149201.1
Vibrio fluvialis strain VL 5125 16S ribosomal RNA, partial sequence	Vibrio fluvialis	2521	2521	99%	0.0	96.19%	1537	NR_036790.1
Vibrio madracius strain A-354 16S ribosomal RNA, partial sequence	Vibrio madracius	2519	2519	95%	0.0	97.37%	1481	NR_178583.1
Vibrio mytili strain CECT 632 16S ribosomal RNA, partial sequence	Vibrio mytili	2519	2519	93%	0.0	98.20%	1441	NR_044911.1
Vibrio hispanicus strain LMG 13240 16S ribosomal RNA, partial sequence	Vibrio hispanicus	2519	2519	98%	0.0	96.65%	1517	NR_042806.1
Vibrio hispanicus strain LMG 13240	Vibrio hispanicus	2518	2518	98%	0.0	96.65%	1516	NR_115180.1

Description	Scientific Name	Max Score	Total Score	Query Cover	E value	Per. Ident	Acc. Len	Accession
16S ribosomal RNA, partial sequence								
Vibrio ziniensis strain ZWAL4003 16S ribosomal RNA, complete sequence	Vibrio ziniensis	2516	2516	100%	0.0	96.03%	1543	NR_181540.1
Vibrio vulnificus strain 324 16S ribosomal RNA, partial sequence	Vibrio vulnificus	2516	2516	99%	0.0	96.13%	1536	NR_036888.1
Vibrio ezurae strain NBRC 102218 16S ribosomal RNA, partial sequence	Vibrio ezurae	2508	2508	95%	0.0	97.09%	1478	NR_114045.1
Vibrio parahaemolyticus strain ATCC 17802 16S ribosomal RNA, partial sequence	Vibrio parahaemolyticus	2507	2507	96%	0.0	97.11%	1481	NR_118928.1
Vibrio atypicus strain HHS02 16S ribosomal RNA, partial sequence	Vibrio atypicus	2505	2505	90%	0.0	98.93%	1401	NR_116535.1
Vibrio mediterranei strain NBRC 15635 16S ribosomal RNA, partial sequence	Vibrio mediterranei	2501	2501	95%	0.0	97.16%	1478	NR_113785.1
Vibrio orientalis strain NBRC 15638 16S ribosomal RNA, partial sequence	Vibrio orientalis	2497	2497	95%	0.0	97.16%	1478	NR_113788.1
Vibrio hangzhouensis strain cn83 16S ribosomal RNA, partial sequence	Vibrio hangzhouensis	2497	2497	95%	0.0	97.22%	1475	NR_044396.1
Vibrio tapetis subsp. britannicus strain HH6087 16S ribosomal RNA, partial sequence	Vibrio tapetis subsp. britannicus	2495	2495	99%	0.0	95.99%	1533	NR_132307.1
Vibrio thalassae strain CECT 8203 16S ribosomal RNA, partial sequence	Vibrio thalassae	2490	2490	94%	0.0	97.34%	1467	NR_134228.1
Vibrio mediterranei strain ATCC 43341 16S ribosomal RNA, partial sequence	Vibrio mediterranei	2490	2490	95%	0.0	97.21%	1471	NR_117896.1
Vibrio tubiashii strain Milford 74 16S ribosomal RNA, partial sequence	Vibrio tubiashii	2488	2488	92%	0.0	97.77%	1436	NR_026129.1
Vibrio caribbeanicus ATCC BAA-2122 strain N384 16S ribosomal RNA, partial sequence	Vibrio caribbeanicus ATCC BAA-2122	2488	2488	95%	0.0	97.22%	1470	NR_108732.1
Vibrio tapetis strain CECT 4600 16S ribosomal RNA, partial sequence	Vibrio tapetis	2486	2486	99%	0.0	95.92%	1531	NR_117643.1
Vibrio proteolyticus strain ATCC 15338 16S ribosomal RNA, partial sequence	Vibrio proteolyticus	2486	2486	96%	0.0	96.71%	1482	NR_118927.1
Vibrio sinaloensis strain CAIM 797 16S ribosomal RNA, partial sequence	Vibrio sinaloensis	2484	2484	96%	0.0	96.78%	1488	NR_043858.1
Vibrio orientalis CIP 102891 = ATCC 33934 16S	Vibrio orientalis CIP 102891 = ATCC 33934	2484	2484	95%	0.0	97.15%	1471	NR_118094.1

Description	Scientific Name	Max Score	Total Score	Query Cover	E value	Per. Ident	Acc. Len	Accession
ribosomal RNA, partial sequence								
Vibrio halioticoli strain NBRC 102217 16S ribosomal RNA, partial sequence	Vibrio halioticoli	2483	2483	95%	0.0	96.89%	1478	NR_114044.1
Vibrio lentus strain 40M4T 16S ribosomal RNA, partial sequence	Vibrio lentus	2479	2479	100%	0.0	95.55%	1559	NR_028926.1
Vibrio artabrorum strain VB 11.8 16S ribosomal RNA, partial sequence	Vibrio artabrorum	2479	2479	99%	0.0	95.67%	1544	NR_116068.1
Vibrio coralliilyticus strain ATCC BAA-450 16S ribosomal RNA, partial sequence	Vibrio coralliilyticus	2479	2479	95%	0.0	97.08%	1471	NR_117892.1
Vibrio neocaledonicus strain NC470 16S ribosomal RNA, partial sequence	Vibrio neocaledonicus	2477	2477	91%	0.0	98.17%	1418	NR_118432.1
Vibrio nereis strain 80 16S ribosomal RNA, partial sequence	Vibrio nereis	2475	2475	92%	0.0	97.56%	1436	NR_026125.1
Vibrio orientalis strain 717 16S ribosomal RNA, partial sequence	Vibrio orientalis	2473	2473	94%	0.0	97.07%	1466	NR_026127.1
Vibrio mediterranei strain 50 16S ribosomal RNA, partial sequence	Vibrio mediterranei	2473	2473	95%	0.0	97.00%	1470	NR_029257.1
Vibrio chagasii strain LMG 21353 16S ribosomal RNA, partial sequence	Vibrio chagasii	2468	2468	95%	0.0	96.95%	1471	NR_117891.1
Vibrio ishigakensis strain C1 16S ribosomal RNA, complete sequence	Vibrio ishigakensis	2466	2466	94%	0.0	96.99%	1460	NR_156028.1
Vibrio bathopelagicus strain Sal10 16S ribosomal RNA, complete sequence	Vibrio bathopelagicus	2466	2466	100%	0.0	94.99%	1555	NR_181507.1
Vibrio jasicida CAIM 1864 = LMG 25398 strain TCFB 0772 16S ribosomal RNA, partial sequence	Vibrio jasicida CAIM 1864 = LMG 25398	2464	2464	90%	0.0	98.43%	1400	NR_113182.1
Vibrio nigripulchritudo ATCC 27043 16S ribosomal RNA, partial sequence	Vibrio nigripulchritudo ATCC 27043	2460	2460	95%	0.0	96.88%	1470	NR_117897.1
Vibrio penaeicida strain DSM 14398 16S ribosomal RNA, partial sequence	Vibrio penaeicida	2459	2459	98%	0.0	96.12%	1504	NR_042121.1

Supplementary Table S3. The KEGG pathway annotation of strain 30D07 and 31D03 at the 1st, 2nd and 3rd KEGG pathway categories.

30D07:	31D03:
**Metabolism**	**Metabolism**
Global and overview maps	Global and overview maps
01100 Metabolic pathways (642)	01100 Metabolic pathways (670)
01110 Biosynthesis of secondary metabolites (269)	01110 Biosynthesis of secondary metabolites (291)
01120 Microbial metabolism in diverse environments (178)	01120 Microbial metabolism in diverse environments (182)
01200 Carbon metabolism (80)	01200 Carbon metabolism (90)
01210 2-Oxocarboxylic acid metabolism (34)	01210 2-Oxocarboxylic acid metabolism (36)
01212 Fatty acid metabolism (18)	01212 Fatty acid metabolism (19)
01230 Biosynthesis of amino acids (92)	01230 Biosynthesis of amino acids (103)
01232 Nucleotide metabolism (43)	01232 Nucleotide metabolism (44)
01250 Biosynthesis of nucleotide sugars (30)	01250 Biosynthesis of nucleotide sugars (30)
01240 Biosynthesis of cofactors (138)	01240 Biosynthesis of cofactors (144)
01220 Degradation of aromatic compounds (6)	01220 Degradation of aromatic compounds (5)
01310 Nitrogen cycle (5)	01310 Nitrogen cycle (7)
01320 Sulfur cycle (11)	01320 Sulfur cycle (8)
Carbohydrate metabolism	Carbohydrate metabolism
00010 Glycolysis / Gluconeogenesis (26)	00010 Glycolysis / Gluconeogenesis (29)
00020 Citrate cycle (TCA cycle) (23)	00020 Citrate cycle (TCA cycle) (23)
00030 Pentose phosphate pathway (21)	00030 Pentose phosphate pathway (21)
00040 Pentose and glucuronate interconversions (7)	00040 Pentose and glucuronate interconversions (15)
00051 Fructose and mannose metabolism (16)	00051 Fructose and mannose metabolism (14)
00052 Galactose metabolism (11)	00052 Galactose metabolism (13)
00053 Ascorbate and aldarate metabolism (5)	00053 Ascorbate and aldarate metabolism (2)
00500 Starch and sucrose metabolism (19)	00500 Starch and sucrose metabolism (18)
00620 Pyruvate metabolism (37)	00620 Pyruvate metabolism (41)
00630 Glyoxylate and dicarboxylate metabolism (22)	00630 Glyoxylate and dicarboxylate metabolism (26)
00640 Propanoate metabolism (23)	00640 Propanoate metabolism (28)
00650 Butanoate metabolism (25)	00650 Butanoate metabolism (22)
00660 C5-Branched dibasic acid metabolism (9)	00660 C5-Branched dibasic acid metabolism (8)
00562 Inositol phosphate metabolism (3)	00562 Inositol phosphate metabolism (3)

30D07:	31D03:
Energy metabolism	Energy metabolism
00190 Oxidative phosphorylation (39)	00190 Oxidative phosphorylation (36)
00195 Photosynthesis (8)	00195 Photosynthesis (8)
00710 Carbon fixation by Calvin cycle (14)	00710 Carbon fixation by Calvin cycle (15)
00720 Other carbon fixation pathways (26)	00720 Other carbon fixation pathways (28)
00680 Methane metabolism (25)	00680 Methane metabolism (30)
00910 Nitrogen metabolism (13)	00910 Nitrogen metabolism (17)
00920 Sulfur metabolism (22)	00920 Sulfur metabolism (20)
Lipid metabolism	Lipid metabolism
00061 Fatty acid biosynthesis (12)	00061 Fatty acid biosynthesis (13)
00071 Fatty acid degradation (10)	00071 Fatty acid degradation (9)
00074 Mycolic acid biosynthesis (3)	00074 Mycolic acid biosynthesis (2)
00120 Primary bile acid biosynthesis (1)	00120 Primary bile acid biosynthesis (1)
00121 Secondary bile acid biosynthesis (1)	00121 Secondary bile acid biosynthesis (1)
00561 Glycerolipid metabolism (10)	00561 Glycerolipid metabolism (10)
00564 Glycerophospholipid metabolism (16)	00564 Glycerophospholipid metabolism (16)
00565 Ether lipid metabolism (1)	00565 Ether lipid metabolism (1)
00600 Sphingolipid metabolism (6)	00600 Sphingolipid metabolism (5)
00590 Arachidonic acid metabolism (1)	00590 Arachidonic acid metabolism (1)
00591 Linoleic acid metabolism (1)	00591 Linoleic acid metabolism (1)
00592 alpha-Linolenic acid metabolism (2)	00592 alpha-Linolenic acid metabolism (2)
01040 Biosynthesis of unsaturated fatty acids (4)	01040 Biosynthesis of unsaturated fatty acids (4)
Nucleotide metabolism	Nucleotide metabolism
00230 Purine metabolism (52)	00230 Purine metabolism (57)
00240 Pyrimidine metabolism (34)	00240 Pyrimidine metabolism (35)

30D07:	31D03:
Amino acid metabolism	Amino acid metabolism
00250 Alanine, aspartate and glutamate metabolism (26)	00250 Alanine, aspartate and glutamate metabolism (25)
00260 Glycine, serine and threonine metabolism (29)	00260 Glycine, serine and threonine metabolism (33)
00270 Cysteine and methionine metabolism (27)	00270 Cysteine and methionine metabolism (30)
00280 Valine, leucine and isoleucine degradation (16)	00280 Valine, leucine and isoleucine degradation (16)
00290 Valine, leucine and isoleucine biosynthesis (12)	00290 Valine, leucine and isoleucine biosynthesis (13)
00300 Lysine biosynthesis (12)	00300 Lysine biosynthesis (14)
00310 Lysine degradation (11)	00310 Lysine degradation (8)
00220 Arginine biosynthesis (15)	00220 Arginine biosynthesis (20)
00330 Arginine and proline metabolism (17)	00330 Arginine and proline metabolism (15)
00340 Histidine metabolism (13)	00340 Histidine metabolism (12)
00350 Tyrosine metabolism (13)	00350 Tyrosine metabolism (11)
00360 Phenylalanine metabolism (8)	00360 Phenylalanine metabolism (7)
00380 Tryptophan metabolism (9)	00380 Tryptophan metabolism (7)
00400 Phenylalanine, tyrosine and tryptophan biosynthesis (20)	00400 Phenylalanine, tyrosine and tryptophan biosynthesis (20)
Metabolism of other amino acids	Metabolism of other amino acids
00410 beta-Alanine metabolism (6)	00410 beta-Alanine metabolism (4)
00430 Taurine and hypotaurine metabolism (5)	00430 Taurine and hypotaurine metabolism (5)
00440 Phosphonate and phosphinate metabolism (3)	00440 Phosphonate and phosphinate metabolism (3)
00450 Selenocompound metabolism (8)	00450 Selenocompound metabolism (9)
00460 Cyanoamino acid metabolism (6)	00460 Cyanoamino acid metabolism (6)
00470 D-Amino acid metabolism (11)	00470 D-Amino acid metabolism (9)
00480 Glutathione metabolism (17)	00480 Glutathione metabolism (18)

30D07:	31D03:
Glycan biosynthesis and metabolism	Glycan biosynthesis and metabolism
00520 Amino sugar and nucleotide sugar metabolism (29)	00520 Amino sugar and nucleotide sugar metabolism (29)
00541 Biosynthesis of various nucleotide sugars (18)	00541 Biosynthesis of various nucleotide sugars (17)
00513 Various types of N-glycan biosynthesis (1)	00513 Various types of N-glycan biosynthesis (1)
00531 Glycosaminoglycan degradation (1)	00531 Glycosaminoglycan degradation (2
00603 Glycosphingolipid biosynthesis - globo and isoglobo series (3)	00603 Glycosphingolipid biosynthesis - globo and isoglobo series (3)
00604 Glycosphingolipid biosynthesis - ganglio series (1)	00604 Glycosphingolipid biosynthesis - ganglio series (1)
00511 Other glycan degradation (5)	00511 Other glycan degradation (6)
00540 Lipopolysaccharide biosynthesis (13)	00540 Lipopolysaccharide biosynthesis (13)
00542 O-Antigen repeat unit biosynthesis (1)	00550 Peptidoglycan biosynthesis (21)
00550 Peptidoglycan biosynthesis (18)	00552 Teichoic acid biosynthesis (4)
00552 Teichoic acid biosynthesis (3)	00543 Exopolysaccharide biosynthesis (2
00572 Arabinogalactan biosynthesis - Mycobacterium (1)	Metabolism of cofactors and vitamins
00543 Exopolysaccharide biosynthesis (3)	00730 Thiamine metabolism (15)
Metabolism of cofactors and vitamins	00740 Riboflavin metabolism (13)
00730 Thiamine metabolism (15)	00750 Vitamin B6 metabolism (8)
00740 Riboflavin metabolism (12)	00760 Nicotinate and nicotinamide metabolism (20)
00750 Vitamin B6 metabolism (8)	00770 Pantothenate and CoA biosynthesis (17)
00760 Nicotinate and nicotinamide metabolism (17)	00780 Biotin metabolism (11)
00770 Pantothenate and CoA biosynthesis (18)	00785 Lipoic acid metabolism (13)
00780 Biotin metabolism (10)	00790 Folate biosynthesis (27)
00785 Lipoic acid metabolism (12)	00670 One carbon pool by folate (18)
00790 Folate biosynthesis (26)	00830 Retinol metabolism (1)
00670 One carbon pool by folate (17)	00860 Porphyrin metabolism (25)
00830 Retinol metabolism (1)	00130 Ubiquinone and other terpenoid-quinone biosynthesis (24)
00860 Porphyrin metabolism (24)	Metabolism of terpenoids and polyketides
00130 Ubiquinone and other terpenoid-quinone biosynthesis (21)	00900 Terpenoid backbone biosynthesis (14)

30D07:	31D03:
00860 Porphyrin metabolism (24)	**Metabolism of terpenoids and polyketides**
00130 Ubiquinone and other terpenoid-quinone biosynthesis (21)	00900 Terpenoid backbone biosynthesis (14)
**Metabolism of terpenoids and polyketides**	00908 Zeatin biosynthesis (1)
00900 Terpenoid backbone biosynthesis (12)	00907 Pinene, camphor and geraniol degradation (4)
00981 Insect hormone biosynthesis (1)	01051 Biosynthesis of ansamycins (1)
00908 Zeatin biosynthesis (1)	00523 Polyketide sugar unit biosynthesis (2)
00903 Limonene degradation (1)	01053 Biosynthesis of siderophore group nonribosomal peptides (1)
00907 Pinene, camphor and geraniol degradation (4)	01055 Biosynthesis of vancomycin group antibiotics (1)
01051 Biosynthesis of ansamycins (1)	**Biosynthesis of other secondary metabolites**
00523 Polyketide sugar unit biosynthesis (1)	00940 Phenylpropanoid biosynthesis (1)
01053 Biosynthesis of siderophore group nonribosomal peptides (8)	00946 Degradation of flavonoids (2)
**Biosynthesis of other secondary metabolites**	00950 Isoquinoline alkaloid biosynthesis (2)
00940 Phenylpropanoid biosynthesis (1)	00960 Tropane, piperidine and pyridine alkaloid biosynthesis (4)
00946 Degradation of flavonoids (2)	00966 Glucosinolate biosynthesis (2)
00950 Isoquinoline alkaloid biosynthesis (2)	00311 Penicillin and cephalosporin biosynthesis (1)
00960 Tropane, piperidine and pyridine alkaloid biosynthesis (4)	00332 Carbapenem biosynthesis (2)
00966 Glucosinolate biosynthesis (2)	00261 Monobactam biosynthesis (8)
00332 Carbapenem biosynthesis (2)	00521 Streptomycin biosynthesis (4)
00261 Monobactam biosynthesis (6)	00525 Acarbose and validamycin biosynthesis (2)
00521 Streptomycin biosynthesis (3)	00401 Novobiocin biosynthesis (4)
00525 Acarbose and validamycin biosynthesis (1)	00405 Phenazine biosynthesis (2)
00401 Novobiocin biosynthesis (4)	00333 Prodigiosin biosynthesis (2)
00405 Phenazine biosynthesis (2)	00998 Biosynthesis of various antibiotics; Including: Kanosamine biosynthesis, Aurachin biosynthesis, Bacilysin biosynthesis, Puromycin biosynthesis, Dapdiamides biosynthesis, Fosfomycin biosynthesis, Cremeomycin biosynthesis, Fumagillin biosynthesis, Pentalenolactone biosynthesis, Terpentecin biosynthesis, Roseoflavin biosynthesis, Cycloserine biosynthesis (1)

30D07:	31D03:
00333 Prodigiosin biosynthesis (2)	00999 Biosynthesis of various plant secondary metabolites; Including: Crocin biosynthesis, Ginsenoside biosynthesis, Saponin adjuvant biosynthesis, Cannabidiol biosynthesis, Mugineic acid biosynthesis, Pentagalloylglucose biosynthesis, Benzoxazinoid biosynthesis, Gramine biosynthesis, Coumarin biosynthesis, Furanocoumarin biosynthesis, Hordatine biosynthesis, Podophyllotoxin biosynthesis (4)
00998 Biosynthesis of various antibiotics; Including: Kanosamine biosynthesis, Aurachin biosynthesis, Bacilysin biosynthesis, Puromycin biosynthesis, Dapdiamides biosynthesis, Fosfomycin biosynthesis, Cremeomycin biosynthesis, Fumagillin biosynthesis, Pentalenolactone biosynthesis, Terpentecin biosynthesis, Roseoflavin biosynthesis, Cycloserine biosynthesis (1)	Xenobiotics biodegradation and metabolism
00999 Biosynthesis of various plant secondary metabolites; Including: Crocin biosynthesis, Ginsenoside biosynthesis, Saponin adjuvant biosynthesis, Cannabidiol biosynthesis, Mugineic acid biosynthesis, Pentagalloylglucose biosynthesis, Benzoxazinoid biosynthesis, Gramine biosynthesis, Coumarin biosynthesis, Furanocoumarin biosynthesis, Hordatine biosynthesis, Podophyllotoxin biosynthesis (4)	00362 Benzoate degradation (6)
Xenobiotics biodegradation and metabolism	00627 Aminobenzoate degradation (2)
00362 Benzoate degradation (14)	00364 Fluorobenzoate degradation (1)
00627 Aminobenzoate degradation (5)	00625 Chloroalkane and chloroalkene degradation (3)
00364 Fluorobenzoate degradation (1)	00361 Chlorocyclohexane and chlorobenzene degradation (1)
00625 Chloroalkane and chloroalkene degradation (4)	00623 Toluene degradation (1)
00361 Chlorocyclohexane and chlorobenzene degradation (1)	00633 Nitrotoluene degradation (1)
00623 Toluene degradation (1)	00642 Ethylbenzene degradation (1)
00633 Nitrotoluene degradation (1)	00643 Styrene degradation (3)
00642 Ethylbenzene degradation (1)	00791 Atrazine degradation (3)
00643 Styrene degradation (3)	00930 Caprolactam degradation (2)

30D07:	31D03:
00998 Biosynthesis of various antibiotics; Including: Kanosamine biosynthesis, Aurachin biosynthesis, Bacilysin biosynthesis, Puromycin biosynthesis, Dapdiamides biosynthesis, Fosfomycin biosynthesis, Cremeomycin biosynthesis, Fumagillin biosynthesis, Pentalenolactone biosynthesis, Terpentecin biosynthesis, Roseoflavin biosynthesis, Cycloserine biosynthesis (1)	Xenobiotics biodegradation and metabolism
00999 Biosynthesis of various plant secondary metabolites; Including: Crocin biosynthesis, Ginsenoside biosynthesis, Saponin adjuvant biosynthesis, Cannabidiol biosynthesis, Mugineic acid biosynthesis, Pentagalloylglucose biosynthesis, Benzoxazinoid biosynthesis, Gramine biosynthesis, Coumarin biosynthesis, Furanocoumarin biosynthesis, Hordatine biosynthesis, Podophyllotoxin biosynthesis (4)	00362 Benzoate degradation (6)
Xenobiotics biodegradation and metabolism	00627 Aminobenzoate degradation (2)
00362 Benzoate degradation (14)	00364 Fluorobenzoate degradation (1)
00627 Aminobenzoate degradation (5)	00625 Chloroalkane and chloroalkene degradation (3)
00364 Fluorobenzoate degradation (1)	00361 Chlorocyclohexane and chlorobenzene degradation (1)
00625 Chloroalkane and chloroalkene degradation (4)	00623 Toluene degradation (1)
00361 Chlorocyclohexane and chlorobenzene degradation (1)	00633 Nitrotoluene degradation (1)
00623 Toluene degradation (1)	00642 Ethylbenzene degradation (1)
00633 Nitrotoluene degradation (1)	00643 Styrene degradation (3)
00642 Ethylbenzene degradation (1)	00791 Atrazine degradation (3)
00643 Styrene degradation (3)	00930 Caprolactam degradation (2)
00930 Caprolactam degradation (2)	00626 Naphthalene degradation (3)
00626 Naphthalene degradation (3)	00984 Steroid degradation (1)
00624 Polycyclic aromatic hydrocarbon degradation (3)	00980 Metabolism of xenobiotics by cytochrome P450 (2)
00984 Steroid degradation (2)	00982 Drug metabolism - cytochrome P450 (2)
00980 Metabolism of xenobiotics by cytochrome P450 (2)	00983 Drug metabolism - other enzymes (12)
[illegible]	

30D07:

00980 Metabolism of xenobiotics by cytochrome P450 (2)
00982 Drug metabolism - cytochrome P450 (2)
00983 Drug metabolism - other enzymes (12)

Genetic Information Processing

Transcription
03020 RNA polymerase (4)

Translation
03010 Ribosome (53)
00970 Aminoacyl-tRNA biosynthesis (22)
03008 Ribosome biogenesis in eukaryotes (2)

Folding, sorting and degradation
03060 Protein export (16)
04141 Protein processing in endoplasmic reticulum (1)
04122 Sulfur relay system (16)
03018 RNA degradation (17)

Replication and repair
03030 DNA replication (13)
03410 Base excision repair (7)
03420 Nucleotide excision repair (4)
03430 Mismatch repair (17)
03440 Homologous recombination (18)

Information processing in viruses
03250 Viral life cycle - HIV-1 (1)

Environmental Information Processing

Membrane transport
02010 ABC transporters (106)
02060 Phosphotransferase system (PTS) (23)
03070 Bacterial secretion system (43)

31D03:

00983 Drug metabolism - other enzymes (12)

Genetic Information Processing

Transcription
03020 RNA polymerase (4)

Translation
03010 Ribosome (54)
00970 Aminoacyl-tRNA biosynthesis (23)
03008 Ribosome biogenesis in eukaryotes (2)

Folding, sorting and degradation
03060 Protein export (16)
04141 Protein processing in endoplasmic reticulum (1)
04122 Sulfur relay system (17)
03018 RNA degradation (16)

Replication and repair
03030 DNA replication (14)
03410 Base excision repair (10)
03420 Nucleotide excision repair (6)
03430 Mismatch repair (18)
03440 Homologous recombination (25)

Information processing in viruses
03250 Viral life cycle - HIV-1 (1)

Environmental Information Processing

Membrane transport
02010 ABC transporters (107)
02060 Phosphotransferase system (PTS) (16)
03070 Bacterial secretion system (32)

Signal transduction
02020 Two-component system (108)

30D07:
02060 Phosphotransferase system (PTS) (23)
03070 Bacterial secretion system (43)
Signal transduction
02020 Two-component system (112)
04013 MAPK signaling pathway - fly (1)
04016 MAPK signaling pathway - plant (3)
04011 MAPK signaling pathway - yeast (1)
04066 HIF-1 signaling pathway (4)
04068 FoxO signaling pathway (2)
04070 Phosphatidylinositol signaling system (3)
04072 Phospholipase D signaling pathway (1)
04151 PI3K-Akt signaling pathway (1)
04152 AMPK signaling pathway (4)
04075 Plant hormone signal transduction (1)
**Cellular Processes**
Transport and catabolism
04142 Lysosome (1)
04146 Peroxisome (8)
04148 Efferocytosis (2)
Cell growth and death
04112 Cell cycle - Caulobacter (12)
04210 Apoptosis (1)
04214 Apoptosis - fly (1)
04216 Ferroptosis (2)
04217 Necroptosis (4)
Cellular community - prokaryotes
02024 Quorum sensing (44)
05111 Biofilm formation - Vibrio cholerae (63)

31D03:
Signal transduction
02020 Two-component system (108)
04013 MAPK signaling pathway - fly (1)
04016 MAPK signaling pathway - plant (3)
04011 MAPK signaling pathway - yeast (1)
04066 HIF-1 signaling pathway (5)
04068 FoxO signaling pathway (2)
04070 Phosphatidylinositol signaling system (3)
04072 Phospholipase D signaling pathway (1)
04151 PI3K-Akt signaling pathway (1)
04152 AMPK signaling pathway (4)
04075 Plant hormone signal transduction (1)
**Cellular Processes**
Transport and catabolism
04142 Lysosome (2)
04146 Peroxisome (7)
04137 Mitophagy - animal (1)
04148 Efferocytosis (2)
Cell growth and death
04112 Cell cycle - Caulobacter (13)
04214 Apoptosis - fly (1)
04216 Ferroptosis (2)
04217 Necroptosis (4)
Cellular community - prokaryotes
02024 Quorum sensing (40)
05111 Biofilm formation - Vibrio cholerae (64)
02025 Biofilm formation - Pseudomonas aeruginosa (21)
02026 Biofilm formation - Escherichia coli (21)

30D07:	31D03:
Cellular community - prokaryotes	05111 Biofilm formation - Vibrio cholerae (64)
02024 Quorum sensing (44)	02025 Biofilm formation - Pseudomonas aeruginosa (21)
05111 Biofilm formation - Vibrio cholerae (63)	02026 Biofilm formation - Escherichia coli (21)
02025 Biofilm formation - Pseudomonas aeruginosa (22)	Cell motility
02026 Biofilm formation - Escherichia coli (22)	02030 Bacterial chemotaxis (17)
Cell motility	02040 Flagellar assembly (45)
02030 Bacterial chemotaxis (16)	04820 Cytoskeleton in muscle cells (1)
02040 Flagellar assembly (44)	
04820 Cytoskeleton in muscle cells (1)	**Organismal Systems**
**Organismal Systems**	Immune system
	04621 NOD-like receptor signaling pathway (3)
Immune system	04612 Antigen processing and presentation (1)
04621 NOD-like receptor signaling pathway (3)	04659 Th17 cell differentiation (1)
04612 Antigen processing and presentation (1)	04657 IL-17 signaling pathway (1)
04659 Th17 cell differentiation (1)	Endocrine system
04657 IL-17 signaling pathway (1)	04910 Insulin signaling pathway (2)
Endocrine system	04922 Glucagon signaling pathway (6)
04910 Insulin signaling pathway (2)	04920 Adipocytokine signaling pathway (1)
04922 Glucagon signaling pathway (4)	03320 PPAR signaling pathway (4)
04920 Adipocytokine signaling pathway (1)	04915 Estrogen signaling pathway (1)
03320 PPAR signaling pathway (4)	04914 Progesterone-mediated oocyte maturation (1)
04915 Estrogen signaling pathway (1)	04917 Prolactin signaling pathway (1)
04914 Progesterone-mediated oocyte maturation (1)	04918 Thyroid hormone synthesis (3)
04917 Prolactin signaling pathway (1)	04919 Thyroid hormone signaling pathway (1)
04918 Thyroid hormone synthesis (3)	04928 Parathyroid hormone synthesis, secretion and action (1)
04919 Thyroid hormone signaling pathway (1)	04614 Renin-angiotensin system (1)
04928 Parathyroid hormone synthesis, secretion and action (1)	Circulatory system
04614 Renin-angiotensin system (1)	04260 Cardiac muscle contraction (3)

30D07:
Circulatory system
04260 Cardiac muscle contraction (3)
Digestive system
04970 Salivary secretion (1)
04972 Pancreatic secretion (1)
04976 Bile secretion (1)
04973 Carbohydrate digestion and absorption (1)
04975 Fat digestion and absorption (1)
04981 Folate transport and metabolism (5)
04980 Cobalamin transport and metabolism (1)
04978 Mineral absorption (2)
Excretory system
04964 Proximal tubule bicarbonate reclamation (1)
Nervous system
04724 Glutamatergic synapse (2)
04727 GABAergic synapse (3)
Development and regeneration
04361 Axon regeneration (1)
04382 Cornified envelope formation (1)
Aging
04211 Longevity regulating pathway (2)
04212 Longevity regulating pathway - worm (7)
04213 Longevity regulating pathway - multiple species (4)
Environmental adaptation
04714 Thermogenesis (7)
04626 Plant-pathogen interaction (5)

31D03:
Digestive system
04970 Salivary secretion (1)
04972 Pancreatic secretion (1)
04976 Bile secretion (1)
04973 Carbohydrate digestion and absorption (1)
04975 Fat digestion and absorption (1)
04981 Folate transport and metabolism (5)
04980 Cobalamin transport and metabolism (1)
04978 Mineral absorption (2)
Excretory system
04964 Proximal tubule bicarbonate reclamation (1)
Nervous system
04724 Glutamatergic synapse (2)
04727 GABAergic synapse (2)
Development and regeneration
04361 Axon regeneration (1)
04382 Cornified envelope formation (1)
Aging
04211 Longevity regulating pathway (2)
04212 Longevity regulating pathway - worm (7)
04213 Longevity regulating pathway - multiple species (3)
Environmental adaptation
04714 Thermogenesis (7)
04626 Plant-pathogen interaction (5)
**Human Diseases**

30D07:
**Human Diseases**
Cancer: overview
05200 Pathways in cancer (2)
05206 MicroRNAs in cancer (2)
05205 Proteoglycans in cancer (1)
05204 Chemical carcinogenesis - DNA adducts (1)
05207 Chemical carcinogenesis - receptor activation (2)
05208 Chemical carcinogenesis - reactive oxygen species (7)
05203 Viral carcinogenesis (1)
05230 Central carbon metabolism in cancer (5)
05231 Choline metabolism in cancer (1)
Cancer: specific types
05225 Hepatocellular carcinoma (1)
05219 Bladder cancer (1)
05215 Prostate cancer (1)
Infectious disease: viral
05165 Human papillomavirus infection (1)
Infectious disease: bacterial
05110 Vibrio cholerae infection (2)
05120 Epithelial cell signaling in Helicobacter pylori infection (1)
05130 Pathogenic Escherichia coli infection (2)
05132 Salmonella infection (5)
05131 Shigellosis (3)
05135 Yersinia infection (2)
05133 Pertussis (3)
05134 Legionellosis (4)
05150 Staphylococcus aureus infection (1)
05152 Tuberculosis (3)
Infectious disease: parasitic
05146 Amoebiasis (1)
05142 Chagas disease (1)
05143 African trypanosomiasis (1)

31D03:
Cancer: overview
05200 Pathways in cancer (3)
05206 MicroRNAs in cancer (1)
05205 Proteoglycans in cancer (1)
05204 Chemical carcinogenesis - DNA adducts (1)
05207 Chemical carcinogenesis - receptor activation (2)
05208 Chemical carcinogenesis - reactive oxygen species (7)
05203 Viral carcinogenesis (1)
05230 Central carbon metabolism in cancer (7)
05231 Choline metabolism in cancer (1)
Cancer: specific types
05225 Hepatocellular carcinoma (2)
05219 Bladder cancer (1)
05215 Prostate cancer (1)
Infectious disease: viral
05165 Human papillomavirus infection (1)
Infectious disease: bacterial
05110 Vibrio cholerae infection (1)
05120 Epithelial cell signaling in Helicobacter pylori infection (2)
05130 Pathogenic Escherichia coli infection (1)
05132 Salmonella infection (4)
05131 Shigellosis (1)
05133 Pertussis (3)
05134 Legionellosis (4)
05150 Staphylococcus aureus infection (1)
05152 Tuberculosis (3)
Infectious disease: parasitic
05146 Amoebiasis (1)
05142 Chagas disease (1)
05143 African trypanosomiasis (1)
Immune disease
05340 Primary immunodeficiency (2)
Neurodegenerative disease

30D07:
05143 African trypanosomiasis (1)
Immune disease
05340 Primary immunodeficiency (2)
Neurodegenerative disease
05010 Alzheimer disease (4)
05012 Parkinson disease (5)
05014 Amyotrophic lateral sclerosis (6)
05016 Huntington disease (6)
05020 Prion disease (4)
05022 Pathways of neurodegeneration - multiple diseases (6)
Cardiovascular disease
05417 Lipid and atherosclerosis (3)
05418 Fluid shear stress and atherosclerosis (3)
05415 Diabetic cardiomyopathy (7)
Endocrine and metabolic disease
04930 Type II diabetes mellitus (1)
04940 Type I diabetes mellitus (2)
04936 Alcoholic liver disease (3)
04932 Non-alcoholic fatty liver disease (3)
04931 Insulin resistance (2)
Drug resistance: antimicrobial
01501 beta-Lactam resistance (13)
01502 Vancomycin resistance (4)
01503 Cationic antimicrobial peptide (CAMP) resistance (17)
Drug resistance: antineoplastic
01524 Platinum drug resistance (2)
01523 Antifolate resistance (4)

31D03:
Neurodegenerative disease
05010 Alzheimer disease (4)
05012 Parkinson disease (4)
05014 Amyotrophic lateral sclerosis (5)
05016 Huntington disease (5)
05020 Prion disease (3)
05022 Pathways of neurodegeneration - multiple diseases (5)
Cardiovascular disease
05417 Lipid and atherosclerosis (3)
05418 Fluid shear stress and atherosclerosis (5)
05415 Diabetic cardiomyopathy (7)
Endocrine and metabolic disease
04930 Type II diabetes mellitus (1)
04940 Type I diabetes mellitus (1)
04936 Alcoholic liver disease (2)
04932 Non-alcoholic fatty liver disease (3)
04931 Insulin resistance (2)
Drug resistance: antimicrobial
01501 beta-Lactam resistance (16)
01502 Vancomycin resistance (5)
01503 Cationic antimicrobial peptide (CAMP) resistance (17)
Drug resistance: antineoplastic
01524 Platinum drug resistance (2)
01523 Antifolate resistance (4)

Supplementary Table S4. Virulence Factor Gene Database output for strains 30D07 and 31D03.

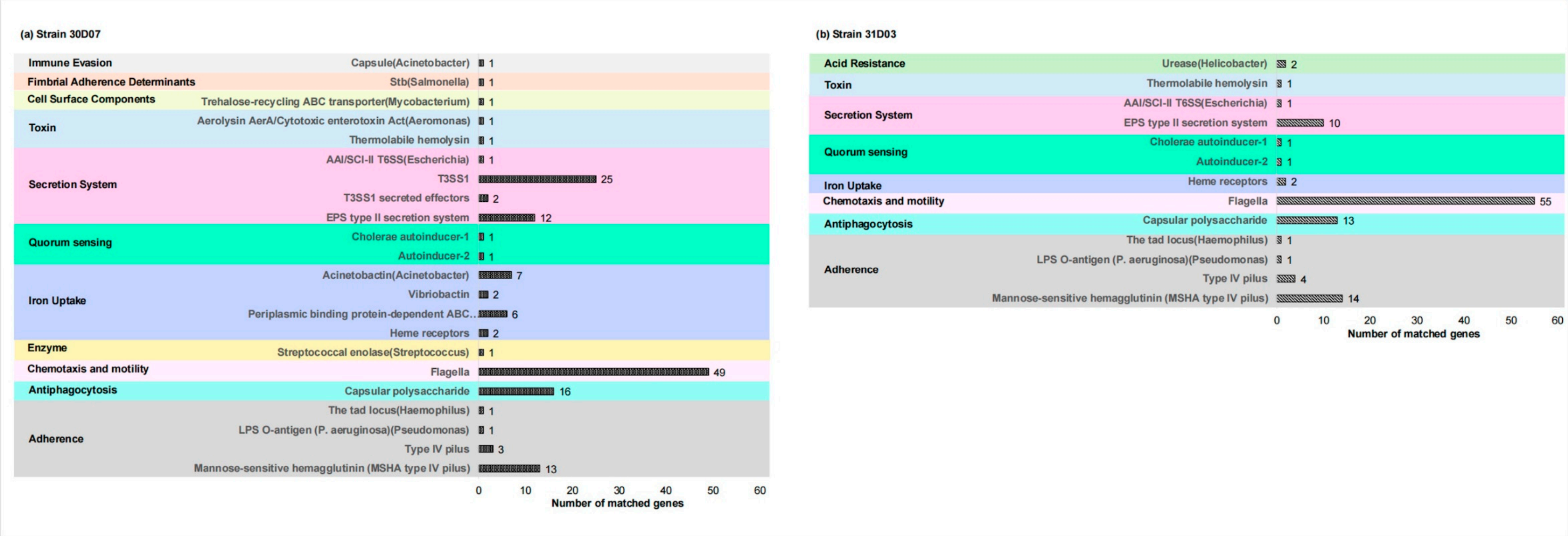

Supplementary Table S5. *In silico* DNA-DNA hybridisation analysis of *Vibrio* strain 30D07 and 31D03 against the NCBI genome reference strains of 162 *Vibrio* spp.

Reference genome	30D07: Formula 1				30D07: Formula 2				30D07: Formula 3				30D07: G+C difference	31D03: Formula 1				31D03: Formula 2				31D03: Formula 3				31D03: G+C difference
	DDH	Model C.I.	Distance	Prob. DDH >=	DDH	Model C.I.	Distance	Prob. DDH >= 70	DDH	Model C.I.	Distance	Prob. DDH >= 70%		DDH	Model C.I.	Distance	Prob. DDH >=	DDH	Model C.I.	Distance	Prob. DDH >=	DDH	Model C.I.	Distance	Prob. DDH >= 70%	
GCA_000024825.1	18.3	[15.2 - 21.9%]	0.7599	0	22.5	[20.3 - 25%]	0.1943	0	18.1	[15.4 - 21.1%]	0.8065	0	0.62	17.4	[14.4 - 20.9%	0.7893	0	22.6	[20.3 - 25%]	0.1941	0	17.3	[14.7 - 20.3%	0.8302	0	0.38
GCA_000165125.2	13.9	[11.2 - 17.3%]	0.9255	0	21.3	[19.1 - 23.8%]	0.2058	0	14.2	[11.8 - 17%]	0.9409	0	3.92	14.1	[11.3 - 17.5%	0.9172	0	21.2	[19 - 23.6%]	0.2071	0	14.4	[11.9 - 17.2%	0.9344	0	2.92
GCA_000176235.1	18.3	[15.2 - 21.9%]	0.7598	0	22.6	[20.4 - 25.1%]	0.1934	0	18.1	[15.4 - 21.1%]	0.8063	0	0.81	19	[15.9 - 22.6%	0.7393	0	22.6	[20.4 - 25.1%	0.1934	0	18.7	[16 - 21.7%]	0.7897	0	0.19
GCA_000189255.2	17.6	[14.5 - 21.1%]	0.7827	0	22	[19.8 - 24.5%]	0.1992	0	17.4	[14.8 - 20.4%]	0.826	0	0.2	17.9	[14.8 - 21.5%	0.7726	0	22	[19.7 - 24.5%	0.1992	0	17.7	[15.1 - 20.7%	0.8179	0	1.19
GCA_000196095.1	41	[37.6 - 44.5%]	0.3827	2.14	24	[21.7 - 26.5%]	0.1817	0.01	35.3	[32.4 - 38.4%]	0.4948	0.02	0.19	38.8	[35.4 - 42.2%	0.4049	1.25	23.8	[21.5 - 26.3%	0.1832	0	33.8	[30.8 - 36.8%	0.5139	0.01	0.81
GCA_000222605.2	15.9	[12.9 - 19.4%]	0.8434	0	22	[19.8 - 24.5%]	0.1989	0	16	[13.4 - 18.9%]	0.8745	0	1.14	16.2	[13.2 - 19.6%	0.8331	0	22.1	[19.9 - 24.6%	0.1982	0	16.2	[13.6 - 19.1%	0.8662	0	0.14
GCA_000287155.2	15.1	[12.2 - 18.6%]	0.8736	0	20.1	[17.9 - 22.5%]	0.2182	0	15.2	[12.7 - 18.1%]	0.9012	0	1.02	15.5	[12.5 - 18.9%	0.8603	0	20.3	[18 - 22.7%]	0.2168	0	15.5	[13 - 18.4%]	0.8906	0	0.02
GCA_000426765.1	13.7	[10.9 - 17%]	0.9372	0	21.1	[18.8 - 23.5%]	0.2085	0	14	[11.6 - 16.8%]	0.9503	0	3.64	13.8	[11 - 17.1%]	0.934	0	21.2	[18.9 - 23.6%	0.2074	0	14.1	[11.6 - 16.9%	0.9477	0	2.64
GCA_000467125.1	16.6	[13.6 - 20.1%]	0.8179	0	20.1	[17.9 - 22.5%]	0.219	0	16.4	[13.9 - 19.4%]	0.8578	0	4.45	16.6	[13.6 - 20.1%	0.8176	0	20.2	[18 - 22.6%]	0.2172	0	16.5	[13.9 - 19.4%	0.8573	0	5.45
GCA_000711795.1	16.4	[13.4 - 19.8%]	0.8262	0	20.6	[18.4 - 23%]	0.213	0	16.3	[13.7 - 19.2%]	0.8632	0	0.35	16.5	[13.5 - 20%]	0.82	0	21	[18.7 - 23.4%	0.2095	0	16.4	[13.9 - 19.4%	0.8577	0	0.65
GCA_000764325.1	17.9	[14.8 - 21.5%]	0.7731	0	20.2	[18 - 22.6%]	0.2176	0	17.5	[14.9 - 20.5%]	0.8224	0	2.84	18.5	[15.4 - 22.1%	0.7543	0	20.2	[17.9 - 22.6%	0.2181	0	18	[15.4 - 21%]	0.8079	0	3.84
GCA_000772105.1	17.6	[14.5 - 21.1%]	0.7834	0	22.6	[20.4 - 25.1%]	0.1935	0	17.5	[14.8 - 20.4%]	0.8253	0	0.56	17.5	[14.5 - 21.1%	0.7852	0	23.1	[20.8 - 25.6%	0.1891	0	17.4	[14.8 - 20.4%	0.8258	0	0.44
GCA_000827885.1	15.9	[12.9 - 19.3%]	0.8447	0	22.1	[19.9 - 24.6%]	0.198	0	15.9	[13.4 - 18.8%]	0.8754	0	0.35	16.2	[13.2 - 19.7%	0.831	0	22.1	[19.8 - 24.6%	0.1983	0	16.2	[13.7 - 19.2%	0.8645	0	0.64
GCA_000967495.1	16.5	[13.5 - 20%]	0.8202	0	21.5	[19.3 - 23.9%]	0.204	0	16.5	[13.9 - 19.4%]	0.8569	0	0.58	16.6	[13.6 - 20.2%	0.8155	0	21.6	[19.3 - 24%]	0.2034	0	16.6	[14 - 19.5%]	0.8531	0	0.42
GCA_000967545.1	17	[14 - 20.5%]	0.8031	0	21.7	[19.4 - 24.1%]	0.2023	0	16.9	[14.3 - 19.8%]	0.8429	0	0.29	17.6	[14.5 - 21.1%	0.7832	0	21.7	[19.4 - 24.1%	0.2023	0	17.4	[14.8 - 20.4%	0.827	0	0.71
GCA_001012815.1	16.1	[13.2 - 19.6%]	0.8342	0	21.8	[19.6 - 24.3%]	0.201	0	16.2	[13.6 - 19.1%]	0.8675	0	0.5	16.5	[13.5 - 20%]	0.8201	0	21.8	[19.5 - 24.2%	0.2014	0	16.5	[13.9 - 19.4%	0.8563	0	0.5
GCA_001274785.1	17.4	[14.4 - 21%]	0.7885	0	22.3	[20 - 24.7%]	0.197	0	17.3	[14.7 - 20.3%]	0.8302	0	0.67	17.5	[14.5 - 21.1%	0.7846	0	22.4	[20.1 - 24.8%	0.196	0	17.4	[14.8 - 20.4%	0.8268	0	1.67
GCA_001399455.2	17.4	[14.3 - 20.9%]	0.7909	0	21.7	[19.5 - 24.2%]	0.2018	0	17.2	[14.6 - 20.2%]	0.8331	0	1.27	17.6	[14.5 - 21.1%	0.7841	0	21.9	[19.6 - 24.3%	0.2006	0	17.4	[14.8 - 20.4%	0.8274	0	0.27
GCA_001456255.1	28.4	[25.1 - 32%]	0.5375	0.05	23	[20.7 - 25.5%]	0.1901	0	26.1	[23.3 - 29.2%]	0.6254	0	0.48	28	[24.7 - 31.7%	0.5439	0.04	22.8	[20.5 - 25.3%	0.1918	0	25.8	[22.9 - 28.9%	0.6314	0	0.52
GCA_001547935.1	14.4	[11.5 - 17.8%]	0.9059	0	22.3	[20 - 24.7%]	0.1968	0	14.6	[12.2 - 17.5%]	0.9244	0	1.67	14.5	[11.6 - 17.9%	0.9022	0	22.4	[20.1 - 24.8%	0.1956	0	14.7	[12.2 - 17.6%	0.9213	0	0.67
GCA_001558415.2	16.2	[13.2 - 19.6%]	0.8334	0	20.7	[18.5 - 23.1%]	0.2124	0	16.1	[13.6 - 19%]	0.8688	0	4.36	16.2	[13.3 - 19.7%	0.8307	0	20.9	[18.7 - 23.4%	0.2099	0	16.2	[13.6 - 19.1%	0.8662	0	5.36
GCA_001591105.1	17.1	[14.1 - 20.6%]	0.7991	0	21.8	[19.5 - 24.2%]	0.2013	0	17	[14.4 - 20%]	0.8395	0	0.13	17.4	[14.4 - 21%]	0.788	0	22.1	[19.9 - 24.6%	0.1981	0	17.3	[14.7 - 20.3%	0.83	0	0.86
GCA_001639065.2	14.9	[12 - 18.3%]	0.8846	0	21.5	[19.2 - 23.9%]	0.2042	0	15	[12.6 - 17.9%]	0.9082	0	1.4	14.8	[12 - 18.2%]	0.8866	0	22.2	[20 - 24.7%]	0.197	0	15	[12.5 - 17.9%	0.9089	0	0.4
GCA_001695575.1	17.5	[14.4 - 21%]	0.7874	0	22	[19.7 - 24.4%]	0.1997	0	17.3	[14.7 - 20.3%]	0.8299	0	0.6	17.4	[14.4 - 21%]	0.7887	0	22.3	[20 - 24.7%]	0.1966	0	17.3	[14.7 - 20.3%	0.8303	0	0.39
GCA_001854765.1	15.2	[12.3 - 18.6%]	0.8724	0	20.8	[18.6 - 23.2%]	0.2111	0	15.3	[12.8 - 18.2%]	0.8993	0	1.22	15.3	[12.4 - 18.8%	0.8649	0	20.8	[18.6 - 23.2%	0.2113	0	15.4	[12.9 - 18.3%	0.8934	0	0.22
GCA_001895205.1	14	[11.2 - 17.4%]	0.9216	0	20.7	[18.5 - 23.2%]	0.2118	0	14.3	[11.9 - 17.1%]	0.9382	0	1.59	14.2	[11.4 - 17.5%	0.9152	0	20.3	[18.1 - 22.7%	0.2164	0	14.4	[12 - 17.2%]	0.9336	0	0.59
GCA_002021755.1	72.4	[68.5 - 76.1%]	0.1707	79.83	40	[37.6 - 42.6%]	0.0993	2.75	65.5	[62.1 - 68.7%]	0.253	52.83	0.12	50.8	[47.4 - 54.2%	0.3009	13.97	31.7	[29.3 - 34.2%	0.1332	0.21	45.5	[42.5 - 48.5%	0.394	0.8	1.12
GCA_002119525.1	16.4	[13.4 - 19.9%]	0.8241	0	22.5	[20.2 - 25%]	0.1947	0	16.4	[13.9 - 19.4%]	0.8584	0	1.5	16.5	[13.5 - 20%]	0.8193	0	22.5	[20.2 - 25%]	0.1947	0	16.5	[14 - 19.5%]	0.8545	0	0.5
GCA_002156525.1	16.4	[13.4 - 19.9%]	0.8259	0	21.8	[19.5 - 24.2%]	0.2014	0	16.4	[13.8 - 19.3%]	0.861	0	1.2	16.6	[13.6 - 20.1%	0.8175	0	21.7	[19.4 - 24.1%	0.2023	0	16.5	[14 - 19.5%]	0.8544	0	0.2
GCA_002157735.2	13.7	[10.9 - 17.1%]	0.9367	0	24	[21.7 - 26.5%]	0.1815	0.01	14	[11.6 - 16.9%]	0.9482	0	3.44	13.8	[11 - 17.2%]	0.9309	0	24.1	[21.8 - 26.6%	0.1809	0.01	14.2	[11.7 - 17%]	0.9434	0	2.45
GCA_002214395.1	53.8	[50.3 - 57.3%]	0.2795	21.54	35.7	[33.3 - 38.2%]	0.1153	0.82	49.3	[46.3 - 52.4%]	0.3626	2.36	0.72	79.6	[75.7 - 83.1%	0.1342	90.65	42.9	[40.4 - 45.5%	0.0901	5.4	72.5	[69 - 75.7%]	0.2122	82.37	0.27
GCA_002218045.2	13.7	[11 - 17.1%]	0.9357	0	23.5	[21.2 - 26%]	0.1859	0	14.1	[11.6 - 16.9%]	0.9476	0	3.26	13.9	[11.1 - 17.2%	0.9283	0	23	[20.7 - 25.5%	0.1903	0	14.2	[11.8 - 17%]	0.942	0	2.26
GCA_002224265.1	18.7	[15.6 - 22.3%]	0.7475	0	21.6	[19.4 - 24.1%]	0.2028	0	18.3	[15.7 - 21.3%]	0.7987	0	1.18	19.9	[16.7 - 23.5%	0.7146	0	21.3	[19 - 23.7%]	0.2064	0	19.3	[16.6 - 22.3%	0.7735	0	2.18
GCA_002257545.1	15.4	[12.5 - 18.8%]	0.8642	0	20.6	[18.3 - 23%]	0.2137	0	15.4	[12.9 - 18.3%]	0.8932	0	1.09	15.9	[13 - 19.4%]	0.8416	0	20.7	[18.5 - 23.1%	0.2123	0	15.9	[13.4 - 18.8%	0.8753	0	0.09
GCA_002749895.1	14.2	[11.4 - 17.6%]	0.9124	0	20.1	[17.9 - 22.5%]	0.2188	0	14.4	[12 - 17.3%]	0.9315	0	2.14	14.6	[11.7 - 18%]	0.8981	0	20.2	[18 - 22.6%]	0.2172	0	14.7	[12.3 - 17.6%	0.9202	0	1.15

Reference genome	30D07: Formula 1				30D07: Formula 2				30D07: Formula 3				30D07: G+C difference	31D03: Formula 1				31D03: Formula 2				31D03: Formula 3				31D03: G+C difference
GCA_002811245.1	16.6	[13.6 - 20.1%]	0.8187	0	21.6	[19.4 - 24.1%]	0.2028	0	16.5	[13.9 - 19.5%]	0.8555	0	0.46	16.7	[13.7 - 20.2%	0.8123	0	21.6	[19.4 - 24.1%	0.2028	0	16.7	[14.1 - 19.6%	0.8504	0	0.54
GCA_002849855.1	18.8	[15.6 - 22.3%]	0.7465	0	23.7	[21.4 - 26.2%]	0.1842	0	18.5	[15.9 - 21.5%]	0.7932	0	3.29	18.7	[15.6 - 22.3%	0.7484	0	23.7	[21.4 - 26.2%	0.1844	0	18.5	[15.8 - 21.5%	0.7948	0	2.29
GCA_002877605.2	16.6	[13.6 - 20.1%]	0.8181	0	21.8	[19.5 - 24.2%]	0.2017	0	16.5	[14 - 19.5%]	0.8548	0	1.42	16.4	[13.4 - 19.9%	0.8237	0	22.1	[19.8 - 24.5%	0.1987	0	16.4	[13.9 - 19.3%	0.8588	0	0.42
GCA_002887615.1	73.1	[69.2 - 76.8%]	0.1671	81.22	33.4	[31 - 35.9%]	0.1253	0.38	62.5	[59.2 - 65.7%]	0.2715	37.03	0.49	49.7	[46.3 - 53.2%	0.3088	11.8	29.8	[27.4 - 32.3%	0.1431	0.1	43.9	[40.9 - 47%]	0.4077	0.5	0.51
GCA_002906475.1	84.6	[80.8 - 87.7%]	0.1094	94.69	74.9	[71.9 - 77.7%]	0.0296	85.64	85.8	[82.7 - 88.4%]	0.1357	98.55	0.01	53.1	[49.6 - 56.6%	0.2844	19.58	32.3	[29.9 - 34.8%	0.1304	0.26	47.4	[44.4 - 50.5%	0.3777	1.41	0.99
GCA_002906655.1	67.2	[63.4 - 70.9%]	0.1984	66.74	31.9	[29.5 - 34.4%]	0.1322	0.23	57.4	[54.2 - 60.5%]	0.3043	15.68	0.62	50.7	[47.3 - 54.2%	0.3015	13.81	29.4	[27 - 31.9%]	0.1457	0.08	44.4	[41.4 - 47.5%	0.4032	0.58	0.38
GCA_003144035.1	13.6	[10.9 - 17%]	0.9406	0	23.1	[20.8 - 25.6%]	0.1894	0	14	[11.6 - 16.8%]	0.9518	0	0.51	13.9	[11.1 - 17.2%	0.9289	0	22.6	[20.3 - 25%]	0.1942	0	14.2	[11.8 - 17%]	0.9427	0	1.51
GCA_003263785.1	15.3	[12.4 - 18.8%]	0.8663	0	22.2	[19.9 - 24.6%]	0.1978	0	15.5	[12.9 - 18.3%]	0.8928	0	1.03	15.2	[12.3 - 18.6%	0.8706	0	22.4	[20.1 - 24.8%	0.1957	0	15.4	[12.9 - 18.2%	0.896	0	2.03
GCA_003311805.1	15	[12.1 - 18.4%]	0.8782	0	20.1	[17.9 - 22.5%]	0.219	0	15.1	[12.6 - 18%]	0.9049	0	1.51	14.8	[11.9 - 18.2%	0.8882	0	20.7	[18.5 - 23.1%	0.2123	0	14.9	[12.5 - 17.8%	0.912	0	2.51
GCA_003335255.1	13.6	[10.8 - 16.9%]	0.9421	0	22.6	[20.3 - 25.1%]	0.1938	0	13.9	[11.5 - 16.7%]	0.9533	0	4.94	13.8	[11 - 17.1%]	0.9342	0	22.9	[20.6 - 25.4%	0.1909	0	14.1	[11.7 - 16.9%	0.9468	0	3.94
GCA_003350295.1	14.7	[11.9 - 18.2%]	0.8898	0	20.6	[18.4 - 23%]	0.2133	0	14.9	[12.4 - 17.8%]	0.9133	0	3.05	14.8	[11.9 - 18.2%	0.8869	0	20.8	[18.6 - 23.2%	0.211	0	15	[12.5 - 17.8%	0.9108	0	2.05
GCA_003350325.1	16.9	[13.9 - 20.4%]	0.8061	0	22.6	[20.4 - 25.1%]	0.1935	0	16.9	[14.3 - 19.8%]	0.8436	0	0.35	17.2	[14.2 - 20.8%	0.7955	0	22.6	[20.3 - 25%]	0.1942	0	17.1	[14.5 - 20.1%	0.8352	0	0.65
GCA_003390515.1	15.1	[12.2 - 18.5%]	0.8757	0	20.3	[18.1 - 22.7%]	0.2168	0	15.2	[12.7 - 18.1%]	0.9026	0	0.93	15.5	[12.6 - 19%]	0.8582	0	20.1	[17.9 - 22.5%	0.2185	0	15.5	[13 - 18.4%]	0.8892	0	0.06
GCA_003415655.1	16.1	[13.1 - 19.6%]	0.8366	0	22.2	[19.9 - 24.7%]	0.1974	0	16.1	[13.6 - 19%]	0.8689	0	1.09	16	[13.1 - 19.5%	0.8374	0	22.4	[20.1 - 24.8%	0.196	0	16.1	[13.6 - 19%]	0.8693	0	0.1
GCA_003568965.1	13.8	[11 - 17.2%]	0.9321	0	22.2	[19.9 - 24.7%]	0.1973	0	14.1	[11.7 - 16.9%]	0.9455	0	2.77	13.9	[11.1 - 17.3%	0.9273	0	22.4	[20.1 - 24.8%	0.1961	0	14.2	[11.8 - 17%]	0.9415	0	1.77
GCA_003568985.1	13.9	[11.1 - 17.2%]	0.9301	0	22.2	[19.9 - 24.6%]	0.1979	0	14.1	[11.7 - 17%]	0.9439	0	2.27	13.7	[11 - 17.1%]	0.9352	0	22.9	[20.6 - 25.3%	0.1913	0	14.1	[11.6 - 16.9%	0.9476	0	1.27
GCA_003569005.1	13.8	[11 - 17.2%]	0.9311	0	23.8	[21.5 - 26.2%]	0.1839	0	14.2	[11.7 - 17%]	0.9437	0	1.02	13.9	[11.1 - 17.3%	0.9263	0	23.5	[21.2 - 25.9%	0.1863	0	14.2	[11.8 - 17.1%	0.94	0	0.02
GCA_003605645.1	15.7	[12.8 - 19.2%]	0.85	0	21.4	[19.1 - 23.8%]	0.2056	0	15.8	[13.3 - 18.7%]	0.8808	0	3.13	15.9	[12.9 - 19.4%	0.8431	0	21.6	[19.4 - 24.1%	0.2027	0	15.9	[13.4 - 18.9%	0.8749	0	2.13
GCA_003716875.1	14	[11.2 - 17.3%]	0.9245	0	21.4	[19.1 - 23.8%]	0.2056	0	14.2	[11.8 - 17.1%]	0.94	0	0.13	14.1	[11.3 - 17.4%	0.9196	0	21.6	[19.3 - 24%]	0.2035	0	14.3	[11.9 - 17.2%	0.9359	0	0.87
GCA_003856525.1	13.7	[10.9 - 17%]	0.9381	0	21.9	[19.7 - 24.4%]	0.1999	0	14	[11.6 - 16.8%]	0.9505	0	3.68	13.8	[11 - 17.2%]	0.9319	0	21.6	[19.4 - 24.1%	0.203	0	14.1	[11.7 - 16.9%	0.9457	0	2.69
GCA_003970385.1	17.8	[14.8 - 21.4%]	0.7753	0	22.1	[19.9 - 24.6%]	0.1981	0	17.6	[15 - 20.6%]	0.8198	0	0.46	18.1	[15 - 21.7%]	0.7667	0	22.5	[20.2 - 25%]	0.1946	0	17.9	[15.2 - 20.9%	0.8121	0	0.54
GCA_004551525.1	17.7	[14.6 - 21.2%]	0.7804	0	22.5	[20.2 - 24.9%]	0.195	0	17.5	[14.9 - 20.5%]	0.8232	0	0.7	18.1	[15 - 21.7%]	0.7669	0	22.8	[20.6 - 25.3%	0.1918	0	17.9	[15.3 - 20.9%	0.8116	0	0.3
GCA_005144905.1	16.4	[13.4 - 19.9%]	0.8239	0	22	[19.7 - 24.4%]	0.1994	0	16.4	[13.8 - 19.3%]	0.8591	0	1.79	16.6	[13.6 - 20.1%	0.8169	0	22.2	[20 - 24.7%]	0.1972	0	16.6	[14 - 19.5%]	0.853	0	0.8
GCA_005281835.1	17.1	[14.1 - 20.6%]	0.7995	0	20.6	[18.4 - 23%]	0.2134	0	16.9	[14.3 - 19.9%]	0.8423	0	1.83	17.3	[14.2 - 20.8%	0.7933	0	20.4	[18.2 - 22.8%	0.2155	0	17.1	[14.5 - 20%]	0.8378	0	0.83
GCA_006364355.1	15.7	[12.8 - 19.1%]	0.8513	0	21.3	[19.1 - 23.8%]	0.2058	0	15.7	[13.2 - 18.7%]	0.8819	0	5.03	15.9	[13 - 19.4%]	0.8428	0	21.1	[18.9 - 23.5%	0.208	0	15.9	[13.4 - 18.8%	0.8755	0	6.03
GCA_007990935.1	18.5	[15.4 - 22%]	0.7558	0	22.8	[20.5 - 25.2%]	0.1922	0	18.2	[15.5 - 21.2%]	0.8027	0	4.92	18.4	[15.3 - 22%]	0.7576	0	22.6	[20.3 - 25%]	0.1942	0	18.1	[15.5 - 21.1%	0.8046	0	3.92
GCA_008369605.1	15	[12.2 - 18.5%]	0.8771	0	20.9	[18.6 - 23.3%]	0.2106	0	15.2	[12.7 - 18.1%]	0.903	0	1.86	15	[12.1 - 18.4%	0.8784	0	21	[18.8 - 23.4%	0.2092	0	15.2	[12.7 - 18%]	0.9038	0	2.86
GCA_009363415.1	14.9	[12.1 - 18.4%]	0.8815	0	21.3	[19 - 23.7%]	0.2062	0	15.1	[12.6 - 18%]	0.906	0	2.91	14.8	[12 - 18.3%]	0.8858	0	21.8	[19.6 - 24.3%	0.2009	0	15	[12.5 - 17.9%	0.9088	0	1.91
GCA_009601765.2	13.6	[10.8 - 16.9%]	0.9422	0	23.7	[21.4 - 26.2%]	0.1842	0	13.9	[11.5 - 16.7%]	0.9528	0	3.17	13.7	[10.9 - 17.1%	0.9369	0	24	[21.7 - 26.5%	0.1817	0.01	14	[11.6 - 16.9%	0.9484	0	2.18
GCA_009665415.1	17.7	[14.6 - 21.2%]	0.7809	0	20.6	[18.4 - 23%]	0.213	0	17.4	[14.8 - 20.4%]	0.8276	0	2.69	18.4	[15.3 - 21.9%	0.7587	0	20.5	[18.3 - 22.9%	0.2145	0	17.9	[15.3 - 20.9%	0.8104	0	3.69
GCA_009811315.1	17.9	[14.8 - 21.4%]	0.7738	0	21.8	[19.6 - 24.3%]	0.2008	0	17.7	[15 - 20.6%]	0.8192	0	1.67	18.2	[15.1 - 21.7%	0.765	0	22.2	[20 - 24.7%]	0.1972	0	17.9	[15.3 - 20.9%	0.8114	0	0.67
GCA_009874545.1	14.2	[11.3 - 17.5%]	0.9162	0	21.2	[18.9 - 23.6%]	0.2076	0	14.4	[12 - 17.2%]	0.9336	0	3.1	14.2	[11.4 - 17.6%	0.9118	0	21.4	[19.2 - 23.8%	0.2051	0	14.5	[12 - 17.3%]	0.9299	0	2.1
GCA_009938185.1	16.2	[13.3 - 19.7%]	0.8308	0	23.4	[21.1 - 25.9%]	0.1867	0	16.3	[13.8 - 19.2%]	0.8624	0	1.27	16.6	[13.6 - 20.2%	0.8155	0	23.3	[21 - 25.7%]	0.188	0	16.7	[14.1 - 19.6%	0.8502	0	0.27
GCA_009938205.1	16.3	[13.3 - 19.8%]	0.8288	0	22.9	[20.6 - 25.4%]	0.1912	0	16.3	[13.8 - 19.3%]	0.8615	0	0.41	16.5	[13.5 - 20%]	0.8202	0	23.1	[20.8 - 25.6%	0.1894	0	16.6	[14 - 19.5%]	0.8542	0	0.59
GCA_009938225.1	16.7	[13.7 - 20.3%]	0.812	0	23.1	[20.8 - 25.5%]	0.1897	0	16.7	[14.2 - 19.7%]	0.8477	0	0.73	17	[14 - 20.5%]	0.8027	0	23.2	[20.9 - 25.7%	0.1886	0	17	[14.4 - 19.9%	0.8399	0	0.27
GCA_010587385.1	15.3	[12.4 - 18.7%]	0.8678	0	23.2	[20.9 - 25.7%]	0.1886	0	15.5	[12.9 - 18.3%]	0.8927	0	0.07	15.3	[12.4 - 18.8%	0.8662	0	23.5	[21.2 - 25.9%	0.1864	0	15.5	[13 - 18.4%]	0.8911	0	1.06

Reference genome	30D07: Formula 1				30D07: Formula 2				30D07: Formula 3				30D07: G+C difference	31D03: Formula 1				31D03: Formula 2				31D03: Formula 3				31D03: G+C difference
GCA_011064285.1	15.3	[12.3 - 18.7%]	0.8687	0	22.2	[20 - 24.7%]	0.1972	0	15.4	[12.9 - 18.3%]	0.8946	0	2.75	15.4	[12.5 - 18.9%	0.8622	0	22.3	[20 - 24.7%]	0.1967	0	15.5	[13 - 18.4%]	0.8893	0	1.76
GCA_011801455.1	39.5	[36.1 - 43%]	0.3975	1.5	24.1	[21.8 - 26.6%]	0.1808	0.01	34.4	[31.4 - 37.4%]	0.5064	0.02	0.66	37.9	[34.5 - 41.3%	0.4145	0.99	23.9	[21.6 - 26.3%	0.183	0	33.1	[30.2 - 36.2%	0.5216	0.01	0.34
GCA_012275705.1	39.3	[35.9 - 42.7%]	0.3999	1.41	23.9	[21.6 - 26.3%]	0.1831	0	34.1	[31.1 - 37.2%]	0.5098	0.01	0.64	36.9	[33.5 - 40.4%	0.4252	0.76	23.6	[21.3 - 26.1%	0.1853	0	32.4	[29.4 - 35.4%	0.5317	0.01	0.36
GCA_012641465.1	14.4	[11.5 - 17.8%]	0.906	0	21.9	[19.6 - 24.3%]	0.2007	0	14.6	[12.2 - 17.5%]	0.9248	0	0.93	14.4	[11.6 - 17.8%	0.9044	0	22	[19.8 - 24.5%	0.1989	0	14.6	[12.2 - 17.5%	0.9234	0	1.93
GCA_013074385.2	14.8	[11.9 - 18.2%]	0.8886	0	20	[17.8 - 22.4%]	0.2201	0	14.9	[12.4 - 17.8%]	0.9131	0	0.07	15	[12.1 - 18.4%	0.8803	0	20.2	[18 - 22.6%]	0.2173	0	15.1	[12.6 - 18%]	0.9063	0	1.07
GCA_013364395.1	15.1	[12.2 - 18.5%]	0.8744	0	22.2	[19.9 - 24.6%]	0.1976	0	15.3	[12.8 - 18.2%]	0.8992	0	1.45	15.1	[12.2 - 18.5%	0.8746	0	22.2	[19.9 - 24.6%	0.1978	0	15.3	[12.8 - 18.2%	0.8994	0	0.46
GCA_014050145.1	16.2	[13.3 - 19.7%]	0.8306	0	21.7	[19.4 - 24.1%]	0.2022	0	16.2	[13.7 - 19.2%]	0.8649	0	1.44	16.5	[13.5 - 20%]	0.82	0	21.9	[19.7 - 24.4%	0.2001	0	16.5	[13.9 - 19.4%	0.856	0	0.45
GCA_014879975.1	16.6	[13.6 - 20.1%]	0.8182	0	22.1	[19.8 - 24.6%]	0.1983	0	16.6	[14 - 19.5%]	0.8542	0	1.48	16.5	[13.5 - 20%]	0.8204	0	22.4	[20.1 - 24.9%	0.1955	0	16.5	[13.9 - 19.5%	0.8555	0	0.48
GCA_015223435.1	13.2	[10.5 - 16.5%]	0.9615	0	23.1	[20.8 - 25.5%]	0.1897	0	13.6	[11.2 - 16.4%]	0.9688	0	5	13.3	[10.6 - 16.6%	0.9559	0	22.4	[20.1 - 24.8%	0.1959	0	13.7	[11.3 - 16.5%	0.9646	0	4
GCA_015278125.1	16.5	[13.5 - 20%]	0.8199	0	22.5	[20.2 - 24.9%]	0.1949	0	16.5	[14 - 19.5%]	0.855	0	1.11	16.7	[13.7 - 20.2%	0.814	0	22.6	[20.3 - 25.1%	0.1938	0	16.7	[14.1 - 19.6%	0.8501	0	0.11
GCA_015686695.1	14.3	[11.5 - 17.7%]	0.9105	0	22.1	[19.8 - 24.5%]	0.1984	0	14.5	[12.1 - 17.4%]	0.9282	0	3.1	14.3	[11.5 - 17.7%	0.9075	0	22.5	[20.2 - 25%]	0.1947	0	14.6	[12.1 - 17.4%	0.9255	0	2.1
GCA_016900875.1	13.4	[10.7 - 16.8%]	0.9502	0	24.2	[21.9 - 26.6%]	0.1805	0.01	13.8	[11.4 - 16.6%]	0.9592	0	0.21	13.6	[10.8 - 16.9%	0.9443	0	24.2	[21.9 - 26.7%	0.1803	0.01	13.9	[11.5 - 16.7%	0.9543	0	0.79
GCA_019226825.1	14.9	[12.1 - 18.4%]	0.8813	0	22	[19.7 - 24.4%]	0.1996	0	15.1	[12.6 - 18%]	0.9049	0	4.62	15	[12.1 - 18.4%	0.8785	0	22.4	[20.1 - 24.8%	0.1958	0	15.2	[12.7 - 18.1%	0.9023	0	5.61
GCA_019670485.1	35.8	[32.4 - 39.3%]	0.4379	0.56	26.7	[24.3 - 29.2%]	0.1623	0.02	32.6	[29.6 - 35.6%]	0.5292	0.01	1.55	36.5	[33.1 - 40%]	0.4292	0.69	26.1	[23.8 - 28.6%	0.1661	0.02	33	[30 - 36%]	0.524	0.01	0.55
GCA_019977755.1	14.3	[11.5 - 17.7%]	0.9092	0	22.7	[20.5 - 25.2%]	0.1925	0	14.6	[12.1 - 17.4%]	0.9267	0	1.65	14.5	[11.7 - 17.9%	0.8997	0	22.4	[20.2 - 24.9%	0.1953	0	14.8	[12.3 - 17.6%	0.9193	0	0.66
GCA_021272385.1	13.7	[10.9 - 17%]	0.9377	0	23	[20.8 - 25.5%]	0.1899	0	14	[11.6 - 16.8%]	0.9495	0	3.41	13.9	[11.1 - 17.2%	0.9288	0	23.1	[20.8 - 25.6%	0.1894	0	14.2	[11.8 - 17%]	0.9423	0	2.41
GCA_021852965.1	18.4	[15.3 - 22%]	0.7561	0	20.6	[18.4 - 23%]	0.213	0	18	[15.4 - 21%]	0.808	0	1.02	19.2	[16.1 - 22.8%	0.7339	0	20.8	[18.6 - 23.3%	0.2107	0	18.7	[16 - 21.7%]	0.79	0	2.01
GCA_022371215.1	16.9	[13.9 - 20.4%]	0.806	0	22	[19.8 - 24.5%]	0.1989	0	16.8	[14.3 - 19.8%]	0.8446	0	1.28	17	[14 - 20.5%]	0.8023	0	22.1	[19.9 - 24.6%	0.198	0	16.9	[14.3 - 19.9%	0.8414	0	0.28
GCA_022812045.1	16.4	[13.4 - 19.8%]	0.826	0	22.3	[20 - 24.7%]	0.1965	0	16.4	[13.8 - 19.3%]	0.8602	0	1.29	16.5	[13.5 - 20%]	0.8224	0	22.1	[19.8 - 24.5%	0.1987	0	16.4	[13.9 - 19.4%	0.8577	0	0.3
GCA_022836845.1	14.5	[11.7 - 17.9%]	0.8996	0	21.4	[19.1 - 23.8%]	0.2052	0	14.7	[12.3 - 17.6%]	0.9202	0	1.04	14.8	[11.9 - 18.2%	0.8872	0	21.5	[19.3 - 24%]	0.2038	0	15	[12.5 - 17.9%	0.9102	0	0.04
GCA_023145695.1	14.9	[12 - 18.3%]	0.8847	0	21.7	[19.4 - 24.1%]	0.2024	0	15	[12.6 - 17.9%]	0.908	0	2.72	15.3	[12.4 - 18.7%	0.8681	0	21.2	[18.9 - 23.6%	0.2074	0	15.4	[12.9 - 18.3%	0.8955	0	1.72
GCA_023634645.1	13.9	[11.1 - 17.3%]	0.928	0	21.3	[19.1 - 23.7%]	0.2061	0	14.2	[11.8 - 17%]	0.9428	0	2.21	14	[11.2 - 17.4%	0.9213	0	21	[18.7 - 23.4%	0.2094	0	14.3	[11.9 - 17.1%	0.9378	0	1.21
GCA_023634655.1	14.3	[11.5 - 17.7%]	0.9092	0	21.6	[19.4 - 24.1%]	0.2029	0	14.5	[12.1 - 17.4%]	0.9276	0	1.28	14.4	[11.6 - 17.8%	0.9038	0	21.6	[19.3 - 24%]	0.2034	0	14.7	[12.2 - 17.5%	0.9234	0	0.28
GCA_023650915.1	39.3	[35.9 - 42.8%]	0.3996	1.42	25	[22.7 - 27.5%]	0.174	0.01	34.6	[31.6 - 37.6%]	0.5041	0.02	0.86	36.3	[32.9 - 39.8%	0.4318	0.65	24.4	[22.1 - 26.9%	0.1789	0.01	32.2	[29.3 - 35.3%	0.5334	0.01	0.14
GCA_023920225.1	13.6	[10.8 - 16.9%]	0.942	0	21.2	[19 - 23.7%]	0.2069	0	13.9	[11.5 - 16.7%]	0.954	0	0.3	13.7	[10.9 - 17.1%	0.9365	0	21.4	[19.2 - 23.9%	0.2046	0	14	[11.6 - 16.8%	0.9495	0	0.69
GCA_024346755.1	13.4	[10.6 - 16.7%]	0.9524	0	21.5	[19.3 - 24%]	0.2038	0	13.7	[11.3 - 16.5%]	0.9621	0	0.16	13.4	[10.7 - 16.8%	0.9501	0	21.9	[19.6 - 24.3%	0.2005	0	13.8	[11.4 - 16.6%	0.9601	0	1.16
GCA_024346795.1	14.2	[11.3 - 17.5%]	0.9159	0	24.1	[21.8 - 26.6%]	0.1812	0.01	14.5	[12 - 17.3%]	0.9311	0	0.51	14.2	[11.4 - 17.5%	0.9154	0	24.3	[22 - 26.8%]	0.1794	0.01	14.5	[12 - 17.3%]	0.9306	0	0.49
GCA_024346835.1	14.1	[11.3 - 17.4%]	0.9204	0	24.1	[21.8 - 26.6%]	0.1813	0.01	14.4	[11.9 - 17.2%]	0.9348	0	1.46	14.2	[11.4 - 17.6%	0.9151	0	24.1	[21.8 - 26.6%	0.1808	0.01	14.5	[12 - 17.3%]	0.9304	0	0.46
GCA_024346875.1	13.8	[11 - 17.1%]	0.9331	0	24.9	[22.6 - 27.4%]	0.1746	0.01	14.1	[11.7 - 16.9%]	0.9448	0	1.77	13.9	[11.1 - 17.3%	0.9277	0	24.8	[22.5 - 27.3%	0.1758	0.01	14.2	[11.8 - 17.1%	0.9404	0	0.77
GCA_024346935.1	13.8	[11 - 17.2%]	0.9315	0	24.5	[22.2 - 27%]	0.1781	0.01	14.2	[11.7 - 17%]	0.9437	0	2.61	13.9	[11.1 - 17.3%	0.9264	0	24.5	[22.2 - 27%]	0.1781	0.01	14.3	[11.8 - 17.1%	0.9395	0	1.61
GCA_024346955.1	13.5	[10.7 - 16.8%]	0.9479	0	22	[19.8 - 24.5%]	0.199	0	13.8	[11.4 - 16.6%]	0.9583	0	0.06	13.7	[10.9 - 17%]	0.9398	0	21.8	[19.5 - 24.2%	0.2013	0	14	[11.6 - 16.8%	0.9519	0	1.05
GCA_024346975.1	14.1	[11.3 - 17.5%]	0.9187	0	23.6	[21.3 - 26.1%]	0.1851	0	14.4	[12 - 17.2%]	0.9337	0	2.39	14.2	[11.3 - 17.5%	0.9157	0	24.2	[21.9 - 26.7%	0.18	0.01	14.5	[12 - 17.3%]	0.9308	0	1.39
GCA_024346995.1	14	[11.2 - 17.4%]	0.9227	0	21.8	[19.5 - 24.2%]	0.2015	0	14.3	[11.9 - 17.1%]	0.9383	0	2.09	14.2	[11.4 - 17.6%	0.9142	0	21.7	[19.5 - 24.1%	0.2021	0	14.4	[12 - 17.3%]	0.9315	0	1.09
GCA_024347015.1	14.6	[11.7 - 18%]	0.8981	0	21.3	[19 - 23.7%]	0.2062	0	14.8	[12.3 - 17.6%]	0.9191	0	4.26	14.8	[12 - 18.3%]	0.8856	0	21.3	[19.1 - 23.7%	0.2062	0	15	[12.5 - 17.9%	0.9092	0	3.26
GCA_024347055.1	14.7	[11.8 - 18.1%]	0.8937	0	21.2	[18.9 - 23.6%]	0.2073	0	14.8	[12.4 - 17.7%]	0.9157	0	0.75	14.6	[11.7 - 18%]	0.8982	0	22	[19.8 - 24.5%	0.1991	0	14.8	[12.3 - 17.6%	0.9185	0	0.25
GCA_024347075.1	13.7	[11 - 17.1%]	0.9355	0	24.8	[22.5 - 27.3%]	0.1758	0.01	14.1	[11.7 - 16.9%]	0.9469	0	2.61	13.8	[11 - 17.2%]	0.9319	0	25.4	[23.1 - 27.9%	0.1713	0.01	14.2	[11.7 - 17%]	0.9436	0	1.61

Reference genome	30D07: Formula 1				30D07: Formula 2				30D07: Formula 3				30D07: G+C difference	31D03: Formula 1				31D03: Formula 2				31D03: Formula 3				31D03: G+C difference
GCA_024347095.1	13.6	[10.8 - 16.9%]	0.9424	0	21.6	[19.4 - 24.1%]	0.2029	0	13.9	[11.5 - 16.7%]	0.9541	0	0.83	13.7	[10.9 - 17%]	0.938	0	22	[19.7 - 24.5%	0.1992	0	14	[11.6 - 16.8%	0.9504	0	1.83
GCA_024347115.1	13.6	[10.8 - 16.9%]	0.9438	0	22	[19.7 - 24.4%]	0.1996	0	13.9	[11.5 - 16.7%]	0.955	0	0.27	13.8	[11 - 17.1%]	0.9347	0	21.8	[19.5 - 24.2%	0.2015	0	14.1	[11.6 - 16.9%	0.9478	0	1.26
GCA_024347135.1	13.5	[10.8 - 16.9%]	0.9461	0	21.8	[19.6 - 24.3%]	0.201	0	13.8	[11.4 - 16.6%]	0.9569	0	0.1	13.6	[10.8 - 16.9%	0.944	0	22.3	[20 - 24.7%]	0.1966	0	13.9	[11.5 - 16.7%	0.955	0	0.9
GCA_024347295.1	16.3	[13.3 - 19.7%]	0.8299	0	22.3	[20 - 24.7%]	0.197	0	16.3	[13.7 - 19.2%]	0.8634	0	1.46	16.7	[13.7 - 20.2%	0.8148	0	22.1	[19.9 - 24.6%	0.1982	0	16.6	[14.1 - 19.6%	0.8515	0	0.46
GCA_024347315.1	16.4	[13.4 - 19.9%]	0.8241	0	22.5	[20.3 - 25%]	0.1945	0	16.4	[13.9 - 19.4%]	0.8583	0	1.57	16.6	[13.6 - 20.1%	0.8177	0	22.5	[20.2 - 24.9%	0.1948	0	16.6	[14 - 19.5%]	0.8532	0	0.57
GCA_024347355.1	17.2	[14.1 - 20.7%]	0.7972	0	22.7	[20.5 - 25.2%]	0.1925	0	17.1	[14.5 - 20.1%]	0.8363	0	1.34	16.9	[13.9 - 20.4%	0.8062	0	22.5	[20.2 - 24.9%	0.1948	0	16.9	[14.3 - 19.8%	0.8439	0	0.35
GCA_024347395.1	15.8	[12.9 - 19.3%]	0.8455	0	21	[18.7 - 23.4%]	0.2094	0	15.9	[13.3 - 18.8%]	0.8778	0	3.11	16.2	[13.3 - 19.7%	0.8306	0	21.4	[19.2 - 23.8%	0.205	0	16.2	[13.7 - 19.1%	0.8653	0	2.11
GCA_024347415.1	16.7	[13.7 - 20.2%]	0.815	0	22.1	[19.8 - 24.5%]	0.1989	0	16.6	[14 - 19.6%]	0.8518	0	1.15	16.6	[13.6 - 20.1%	0.8171	0	22.4	[20.1 - 24.8%	0.1958	0	16.6	[14 - 19.5%]	0.8529	0	0.15
GCA_024347455.1	16.4	[13.4 - 19.9%]	0.826	0	21.7	[19.5 - 24.2%]	0.2019	0	16.3	[13.8 - 19.3%]	0.8611	0	1.9	16.4	[13.4 - 19.9%	0.8243	0	22.2	[19.9 - 24.6%	0.1978	0	16.4	[13.8 - 19.3%	0.859	0	0.91
GCA_024347475.1	17.1	[14.1 - 20.6%]	0.8001	0	22.6	[20.3 - 25.1%]	0.1938	0	17	[14.4 - 20%]	0.8388	0	0.84	17.3	[14.3 - 20.8%	0.7927	0	22.2	[20 - 24.7%]	0.1972	0	17.2	[14.6 - 20.2%	0.8336	0	0.16
GCA_024347495.1	16	[13 - 19.5%]	0.8402	0	21.9	[19.7 - 24.4%]	0.1998	0	16	[13.5 - 18.9%]	0.8722	0	4.16	16.4	[13.4 - 19.8%	0.8261	0	21.9	[19.7 - 24.4%	0.2	0	16.4	[13.8 - 19.3%	0.8609	0	3.16
GCA_024347535.1	16.6	[13.6 - 20.2%]	0.8155	0	21.9	[19.7 - 24.4%]	0.2	0	16.6	[14 - 19.5%]	0.8524	0	1.62	16.6	[13.6 - 20.1%	0.817	0	22.1	[19.9 - 24.6%	0.198	0	16.6	[14 - 19.5%]	0.8533	0	0.62
GCA_024347575.1	17.4	[14.4 - 21%]	0.7887	0	22.5	[20.2 - 24.9%]	0.1951	0	17.3	[14.7 - 20.3%]	0.8299	0	0.74	16.9	[13.9 - 20.4%	0.8058	0	22.9	[20.6 - 25.4%	0.1911	0	16.9	[14.3 - 19.8%	0.8429	0	0.26
GCA_024347595.1	16.9	[13.9 - 20.4%]	0.8061	0	22.3	[20 - 24.7%]	0.1966	0	16.9	[14.3 - 19.8%]	0.8442	0	0.98	16.9	[13.9 - 20.4%	0.8066	0	22.4	[20.1 - 24.8%	0.1958	0	16.8	[14.3 - 19.8%	0.8445	0	0.02
GCA_024347635.1	16.4	[13.4 - 19.9%]	0.8252	0	22.6	[20.3 - 25.1%]	0.1938	0	16.4	[13.8 - 19.3%]	0.859	0	1.47	16.6	[13.6 - 20.1%	0.8166	0	22.5	[20.3 - 25%]	0.1944	0	16.6	[14 - 19.6%]	0.8522	0	0.48
GCA_024347655.1	16.7	[13.7 - 20.2%]	0.8144	0	21.9	[19.6 - 24.3%]	0.2005	0	16.6	[14.1 - 19.6%]	0.8516	0	1.54	16.8	[13.8 - 20.3%	0.8109	0	21.9	[19.7 - 24.4%	0.2001	0	16.7	[14.1 - 19.7%	0.8487	0	0.54
GCA_024347675.1	14.3	[11.5 - 17.7%]	0.9086	0	24.1	[21.8 - 26.5%]	0.1813	0.01	14.6	[12.2 - 17.5%]	0.9252	0	0.68	14.3	[11.5 - 17.7%	0.9084	0	24.3	[22 - 26.8%]	0.1796	0.01	14.6	[12.2 - 17.5%	0.9249	0	1.68
GCA_024529975.1	13.3	[10.5 - 16.6%]	0.9585	0	22.5	[20.3 - 25%]	0.1945	0	13.6	[11.2 - 16.4%]	0.9666	0	0.2	13.3	[10.6 - 16.6%	0.9575	0	22.8	[20.5 - 25.2%	0.1923	0	13.6	[11.3 - 16.4%	0.9657	0	1.2
GCA_024582835.1	17.5	[14.4 - 21%]	0.7862	0	22.8	[20.6 - 25.3%]	0.1917	0	17.4	[14.8 - 20.4%]	0.8272	0	0.53	17.7	[14.7 - 21.3%	0.7791	0	22.6	[20.4 - 25.1%	0.1936	0	17.6	[14.9 - 20.5%	0.8219	0	0.47
GCA_025195085.2	17.1	[14.1 - 20.6%]	0.8001	0	22.3	[20 - 24.7%]	0.1969	0	17	[14.4 - 20%]	0.8395	0	0.83	17.3	[14.3 - 20.8%	0.7924	0	22.3	[20 - 24.8%]	0.1964	0	17.2	[14.6 - 20.2%	0.8332	0	0.17
GCA_026001925.1	13.9	[11.1 - 17.2%]	0.9286	0	23.7	[21.4 - 26.1%]	0.1848	0	14.2	[11.8 - 17%]	0.9418	0	2.45	14	[11.2 - 17.3%	0.9245	0	23.8	[21.5 - 26.2%	0.1839	0	14.3	[11.9 - 17.1%	0.9384	0	1.46
GCA_026191655.1	17	[14 - 20.5%]	0.803	0	21.6	[19.3 - 24%]	0.2032	0	16.9	[14.3 - 19.8%]	0.843	0	0.87	17	[13.9 - 20.5%	0.8043	0	21.7	[19.5 - 24.2%	0.2018	0	16.9	[14.3 - 19.8%	0.8438	0	0.13
GCA_026191675.1	14.3	[11.5 - 17.7%]	0.9072	0	21.8	[19.6 - 24.3%]	0.2009	0	14.6	[12.1 - 17.4%]	0.9259	0	1.27	14.3	[11.5 - 17.7%	0.9091	0	21.9	[19.6 - 24.3%	0.2005	0	14.6	[12.1 - 17.4%	0.9273	0	0.27
GCA_027924785.1	14.4	[11.5 - 17.8%]	0.9058	0	23.5	[21.2 - 26%]	0.1857	0	14.7	[12.2 - 17.5%]	0.9233	0	0.33	14.6	[11.7 - 18%]	0.8972	0	23.3	[21 - 25.8%]	0.1878	0	14.8	[12.4 - 17.7%	0.9165	0	1.33
GCA_028204155.1	13.6	[10.8 - 16.9%]	0.9426	0	23.8	[21.5 - 26.3%]	0.1836	0	13.9	[11.5 - 16.7%]	0.9531	0	4.74	13.7	[10.9 - 17%]	0.9381	0	23.4	[21.1 - 25.8%	0.187	0	14	[11.6 - 16.8%	0.9497	0	3.75
GCA_028858425.1	16.1	[13.2 - 19.6%]	0.8339	0	20.9	[18.7 - 23.3%]	0.21	0	16.1	[13.6 - 19%]	0.8688	0	2.77	16.5	[13.5 - 20%]	0.8214	0	21.2	[18.9 - 23.6%	0.2072	0	16.4	[13.9 - 19.4%	0.8584	0	1.77
GCA_028863985.1	14.1	[11.3 - 17.5%]	0.9177	0	20.9	[18.7 - 23.3%]	0.2101	0	14.4	[11.9 - 17.2%]	0.935	0	1.48	14.2	[11.4 - 17.6%	0.9128	0	21.4	[19.1 - 23.8%	0.2051	0	14.5	[12 - 17.3%]	0.9307	0	2.48
GCA_029541605.1	17.1	[14 - 20.6%]	0.8012	0	22.1	[19.9 - 24.6%]	0.1982	0	17	[14.4 - 19.9%]	0.8406	0	0.29	17.2	[14.2 - 20.8%	0.7948	0	22.4	[20.1 - 24.8%	0.1958	0	17.1	[14.5 - 20.1%	0.8349	0	1.29
GCA_029589055.1	14.1	[11.3 - 17.5%]	0.9182	0	23.5	[21.2 - 25.9%]	0.1862	0	14.4	[12 - 17.2%]	0.9334	0	1.76	14.2	[11.4 - 17.6%	0.9142	0	23.1	[20.8 - 25.6%	0.1895	0	14.5	[12 - 17.3%]	0.9305	0	0.77
GCA_029635775.1	14.3	[11.5 - 17.7%]	0.9093	0	21.6	[19.4 - 24%]	0.203	0	14.5	[12.1 - 17.4%]	0.9277	0	1.43	14.4	[11.6 - 17.8%	0.9045	0	22.1	[19.8 - 24.6%	0.1983	0	14.6	[12.2 - 17.5%	0.9234	0	2.43
GCA_030060435.1	65.4	[61.6 - 69.1%]	0.2083	61.15	35.6	[33.1 - 38.1%]	0.1157	0.79	58.1	[54.9 - 61.2%]	0.2999	17.82	0.59	52.7	[49.2 - 56.1%	0.2875	18.41	31.4	[29 - 33.9%]	0.1349	0.18	46.7	[43.7 - 49.8%	0.3836	1.15	0.41
GCA_030161215.1	13.7	[10.9 - 17%]	0.9384	0	22.3	[20 - 24.7%]	0.1969	0	14	[11.6 - 16.8%]	0.9506	0	4.84	13.8	[11.1 - 17.2%	0.9306	0	22.5	[20.2 - 24.9%	0.195	0	14.1	[11.7 - 17%]	0.9441	0	3.84
GCA_030161235.1	14.5	[11.6 - 17.9%]	0.9014	0	20.2	[18 - 22.6%]	0.2176	0	14.7	[12.2 - 17.5%]	0.9228	0	2.72	14.5	[11.6 - 17.9%	0.9023	0	19.9	[17.7 - 22.3%	0.2207	0	14.6	[12.2 - 17.5%	0.9239	0	1.72
GCA_030297155.1	16.5	[13.5 - 20%]	0.8215	0	21.9	[19.7 - 24.4%]	0.2	0	16.5	[13.9 - 19.4%]	0.8572	0	1.38	16.3	[13.3 - 19.8%	0.8295	0	22.5	[20.2 - 24.9%	0.1949	0	16.3	[13.7 - 19.2%	0.8627	0	0.38
GCA_030400295.1	15.1	[12.2 - 18.5%]	0.875	0	24	[21.7 - 26.5%]	0.182	0	15.3	[12.8 - 18.2%]	0.8977	0	0.13	15.4	[12.5 - 18.9%	0.8623	0	23.7	[21.4 - 26.1%	0.1847	0	15.6	[13.1 - 18.5%	0.8877	0	0.86
GCA_030409945.1	14.4	[11.6 - 17.8%]	0.9042	0	20.8	[18.5 - 23.2%]	0.2115	0	14.6	[12.2 - 17.5%]	0.9245	0	1.67	14.5	[11.6 - 17.9%	0.9024	0	21	[18.7 - 23.4%	0.2096	0	14.7	[12.2 - 17.5%	0.9229	0	0.67

Reference genome	30D07: Formula 1				30D07: Formula 2				30D07: Formula 3				30D07: G+C difference	31D03: Formula 1				31D03: Formula 2				31D03: Formula 3				31D03: G+C difference
GCA_030580635.1	15.2	[12.3 - 18.7%]	0.87	0	20.7	[18.5 - 23.1%]	0.212	0	15.3	[12.8 - 18.2%]	0.8975	0	1.35	15.2	[12.3 - 18.7%	0.8692	0	21.2	[19 - 23.6%]	0.207	0	15.4	[12.9 - 18.2%	0.8963	0	2.35
GCA_033542615.1	15.9	[13 - 19.4%]	0.8427	0	21.4	[19.2 - 23.8%]	0.205	0	15.9	[13.4 - 18.9%]	0.8749	0	1.71	15.9	[12.9 - 19.3%	0.8444	0	21.9	[19.6 - 24.3%	0.2007	0	15.9	[13.4 - 18.8%	0.8757	0	0.71
GCA_036864355.1	16.6	[13.6 - 20.1%]	0.8161	0	21.6	[19.4 - 24.1%]	0.2028	0	16.6	[14 - 19.5%]	0.8534	0	1.02	16.6	[13.6 - 20.1%	0.8169	0	21.8	[19.6 - 24.2%	0.2012	0	16.6	[14 - 19.5%]	0.8537	0	0.03
GCA_037672085.1	15	[12.1 - 18.5%]	0.878	0	20.8	[18.5 - 23.2%]	0.2115	0	15.2	[12.7 - 18%]	0.9038	0	1.66	14.9	[12 - 18.3%]	0.8824	0	21.2	[18.9 - 23.6%	0.2073	0	15.1	[12.6 - 17.9%	0.9068	0	2.66
GCA_038452265.1	15.8	[12.8 - 19.2%]	0.8486	0	21.2	[19 - 23.6%]	0.2069	0	15.8	[13.3 - 18.7%]	0.8799	0	2.02	15.7	[12.7 - 19.1%	0.8517	0	21.6	[19.4 - 24%]	0.2031	0	15.8	[13.2 - 18.7%	0.8819	0	1.02
GCA_040412085.2	15.5	[12.5 - 18.9%]	0.8602	0	22.5	[20.2 - 25%]	0.1946	0	15.6	[13.1 - 18.5%]	0.8874	0	0.55	15.4	[12.5 - 18.8%	0.8635	0	22.7	[20.4 - 25.1%	0.1931	0	15.5	[13 - 18.4%]	0.8898	0	1.55
GCA_040869025.1	16.2	[13.3 - 19.7%]	0.8305	0	22.8	[20.5 - 25.3%]	0.192	0	16.3	[13.7 - 19.2%]	0.863	0	0.81	16.5	[13.5 - 20%]	0.8212	0	23	[20.7 - 25.5%	0.1903	0	16.5	[14 - 19.5%]	0.8552	0	0.19
GCA_042428525.1	14.8	[11.9 - 18.2%]	0.888	0	20.4	[18.2 - 22.8%]	0.215	0	14.9	[12.5 - 17.8%]	0.9121	0	0.34	15	[12.1 - 18.4%	0.8789	0	20.3	[18.1 - 22.7%	0.2164	0	15.1	[12.6 - 18%]	0.9051	0	0.65
GCA_042430465.1	17.6	[14.5 - 21.1%]	0.7829	0	22.1	[19.8 - 24.5%]	0.1986	0	17.4	[14.8 - 20.4%]	0.826	0	0.12	17.6	[14.6 - 21.2%	0.7822	0	22.4	[20.1 - 24.9%	0.1955	0	17.5	[14.8 - 20.4%	0.8248	0	1.12
GCA_900089835.1	14.8	[11.9 - 18.2%]	0.8868	0	21.7	[19.4 - 24.1%]	0.2024	0	15	[12.5 - 17.9%]	0.9097	0	1.22	14.9	[12 - 18.3%]	0.8822	0	21.8	[19.5 - 24.2%	0.2013	0	15.1	[12.6 - 18%]	0.9059	0	0.22
GCA_900100015.1	14.9	[12.1 - 18.4%]	0.882	0	20.3	[18.1 - 22.7%]	0.2163	0	15.1	[12.6 - 17.9%]	0.9075	0	0.83	15	[12.1 - 18.4%	0.8807	0	20.5	[18.3 - 22.9%	0.2146	0	15.1	[12.6 - 18%]	0.9063	0	1.83
GCA_900107935.1	14.7	[11.9 - 18.1%]	0.8904	0	21.5	[19.2 - 23.9%]	0.2044	0	14.9	[12.4 - 17.8%]	0.9128	0	1.01	14.9	[12 - 18.3%]	0.8828	0	21.5	[19.2 - 23.9%	0.2042	0	15.1	[12.6 - 17.9%	0.9067	0	2
GCA_900460255.1	14.3	[11.5 - 17.7%]	0.9102	0	21.8	[19.5 - 24.2%]	0.2014	0	14.5	[12.1 - 17.4%]	0.9283	0	1.78	14.7	[11.9 - 18.1%	0.8913	0	21.3	[19.1 - 23.7%	0.2061	0	14.9	[12.4 - 17.8%	0.9137	0	0.79
GCA_900460295.1	14.2	[11.4 - 17.6%]	0.9118	0	21.6	[19.3 - 24%]	0.2032	0	14.5	[12 - 17.3%]	0.9297	0	1.41	14.4	[11.5 - 17.7%	0.9069	0	21.8	[19.6 - 24.2%	0.2011	0	14.6	[12.1 - 17.4%	0.9256	0	0.41
GCA_900460385.1	15.4	[12.5 - 18.9%]	0.8625	0	20.8	[18.6 - 23.2%]	0.211	0	15.5	[13 - 18.4%]	0.8915	0	0.77	15.5	[12.6 - 18.9%	0.8588	0	21.3	[19 - 23.7%]	0.2065	0	15.6	[13.1 - 18.5%	0.888	0	1.77
GCA_921292975.1	14.4	[11.6 - 17.8%]	0.9041	0	23.3	[21 - 25.8%]	0.1875	0	14.7	[12.2 - 17.5%]	0.9221	0	0.4	14.5	[11.6 - 17.9%	0.902	0	23.9	[21.6 - 26.4%	0.1828	0	14.7	[12.3 - 17.6%	0.92	0	0.59
GCA_921293875.1	13.3	[10.5 - 16.6%]	0.9589	0	25.2	[22.9 - 27.7%]	0.1728	0.01	13.6	[11.3 - 16.4%]	0.966	0	1.91	13.4	[10.6 - 16.7%	0.9526	0	25.3	[23 - 27.8%]	0.1717	0.01	13.8	[11.4 - 16.5%	0.9607	0	2.91
GCA_921294215.1	14.3	[11.4 - 17.6%]	0.9113	0	23	[20.7 - 25.5%]	0.1902	0	14.5	[12.1 - 17.4%]	0.9282	0	3.23	14.3	[11.5 - 17.7%	0.9092	0	22.9	[20.7 - 25.4%	0.1909	0	14.6	[12.1 - 17.4%	0.9265	0	2.23
GCA_965136275.1	24.3	[21 - 27.9%]	0.6117	0.01	21.8	[19.6 - 24.3%]	0.201	0	22.8	[20 - 25.9%]	0.6897	0	1.09	24.5	[21.2 - 28.2%	0.6072	0.01	21.8	[19.5 - 24.2%	0.2013	0	22.9	[20.1 - 26%]	0.6863	0	0.1

Supplementary Table S6. ANIb (6a) and ANIm (6b) identities across genomes of strain 30D07, 31D03 and the NCBI reference strains representing 21 *Vibrio* spp. based on (a) ANIb and (b) ANIm analysis results.

6a:

ANIb identity (%):	Vibrio parahaemolyticus RIMD 2210633.fna	Vibrio harvey SB1.fna	Vibrio owenssi XSBZ03.fna	Vibrio jasicida 090810c.fna	Vibrio vulnificus ATCC27562.fna	Vibrio rotiferianus B64D1.fna	Vibrio campbellii BoB53.fna	30D07.fna	31D03.fasta	Vibrio natriegens ATCC14048.fna	Vibrio fluvialis ATCC33809.fna	Vibrio furnissii FDAARGOS 777.fna	Vibrio diabolicus FA3.fna	Vibrio alginolyticus E110.fna	Vibrio coralliilyticus Rb102.fna	Vibrio ichthyoenteri ATCC700023.fna	Vibrio anguillarum PF4-E1-1.fna	Vibrio cyclitrophicus ECSMB14105.fna	Vibrio scophthalmi 20FBVib0200.fna	Vibrio mytili JXOK01.fna	Vibrio mediterranei Vic-OC-097.fna	Vibrio splendidus 2 C04b.fna	Vibrio crassostreae DSM17220.fna
**Vibrio parahaemolyticus RIMD 2210633.fna**	*	80.16	80.34	79.86	74.33	79.35	80.29	80.14	79.44	80.07	72.84	72.88	83.87	83.05	73.64	73.49	72.56	73.48	73.58	78.33	72.4	73.49	73.63
**Vibrio harvey SB1.fna**	80.06	*	88.36	86.27	74.46	85.77	87.82	87.87	84.92	78.57	73.07	72.9	79.88	80.48	73.72	73.76	72.7	73.82	73.8	78.2	72.59	73.81	74.05
**Vibrio owenssi XSBZ03.fna**	80.08	88.19	*	89.31	74.53	85.82	89.77	89.61	84.93	78.54	72.98	72.89	80.03	80.86	73.76	73.5	72.48	73.82	73.53	77.45	72.27	73.83	74.03
**Vibrio jasicida 090810c.fna**	79.63	86.12	89.25	*	74.23	84.65	87.07	86.97	84.08	78.28	72.69	72.74	79.59	80.2	73.46	73.42	72.47	73.63	73.48	77.3	72.1	73.56	73.77
**Vibrio vulnificus ATCC27562.fna**	74.56	74.4	74.68	74.46	*	74.42	74.67	74.6	74.42	74.07	73.08	73.12	74.22	74.21	72.87	73.06	73.05	72.71	73.06	73.82	71.84	72.6	72.82
**Vibrio rotiferianus B64D1.fna**	79.38	85.83	86.06	84.88	74.48	*	86.8	86.9	90.67	78.25	72.88	72.79	79.47	79.88	73.87	73.57	72.62	73.95	73.64	77.28	72.64	73.94	73.94
**Vibrio campbellii BoB53.fna**	80.41	87.92	89.99	87.35	74.71	86.84	*	96.89	85.54	78.84	73.18	73.08	80.19	80.57	73.97	73.61	72.63	74.03	73.73	77.62	72.64	73.9	74.16
**30D07.fna**	80.04	87.85	89.76	87.11	74.48	86.73	96.77	*	85.41	78.55	73.02	72.95	79.87	80.49	73.72	73.6	72.59	73.83	73.73	77.54	72.48	73.91	74.05
**31D03.fasta**	79.45	85.05	85.2	84.34	74.47	90.63	85.47	85.6	*	78.18	73.03	72.91	79.34	79.84	74.01	73.63	72.75	74.06	73.76	77.25	72.73	73.92	74.06

ANIb identity (%):	Vibrio parahaemolyticus RIMD 2210633.fna	Vibrio harvey SB1.fna	Vibrio owenssi XSBZ03.fna	Vibrio jasicida 090810c.fna	Vibrio vulnificus ATCC27562.fna	Vibrio rotiferianus B64D1.fna	Vibrio campbellii BoB53.fna	30D07.fna	31D03.fasta	Vibrio natriegens ATCC14048.fna	Vibrio fluvialis ATCC33809.fna	Vibrio furnissii FDAARGOS 777.fna	Vibrio diabolicus FA3.fna	Vibrio alginolyticus E110.fna	Vibrio coralliilyticus Rb102.fna	Vibrio ichthyoenteri ATCC700023.fna	Vibrio anguillarum PF4-E1-1.fna	Vibrio cyclitrophicus ECSMB14105.fna	Vibrio scophthalmi 20FBVib0200.fna	Vibrio mytili JXOK01.fna	Vibrio mediterranei Vic-OC-097.fna	Vibrio splendidus 2 C04b.fna	Vibrio crassostreae DSM17220.fna
Vibrio natriegens ATCC14048.fna	80.11	78.57	78.6	78.49	73.98	78.16	78.79	78.63	78.1	*	72.83	72.72	79.58	79.4	73.66	73.29	72.58	73.46	73.4	78.59	72.35	73.47	73.43
Vibrio fluvialis ATCC33809.fna	73.08	73.03	73.15	72.92	73.05	72.94	73.23	73.12	72.99	72.97	*	85.6	72.77	72.64	72.74	72.42	73.6	72.21	72.71	72.53	71.52	72.2	72.4
Vibrio furnissii FDAARGOS 777.fna	73.01	72.79	72.83	72.79	73.11	72.72	73.12	72.95	72.87	72.81	85.56	*	72.76	72.66	72.48	72.68	73.64	72.04	72.8	72.6	71.39	72.09	72.28
Vibrio diabolicus FA3.fna	83.91	80.01	80.33	79.79	74.2	79.45	80.16	79.95	79.37	79.67	72.79	72.86	*	91.55	73.49	73.38	72.65	73.44	73.49	78.61	72.36	73.39	73.52
Vibrio alginolyticus E110.fna	83.04	80.56	81.07	80.33	74.18	79.87	80.67	80.66	79.83	79.51	72.7	72.7	91.58	*	73.57	73.32	72.58	73.44	73.43	78.33	72.24	73.4	73.63
Vibrio coralliilyticus Rb102.fna	73.58	73.68	73.75	73.62	72.8	73.7	73.96	73.83	73.91	73.64	72.57	72.52	73.43	73.41	*	73.5	72.34	72.93	73.65	73.24	72.07	72.81	72.99
Vibrio ichthyoenteri ATCC700023.fna	73.45	73.48	73.39	73.35	72.77	73.39	73.45	73.41	73.51	73.22	72.33	72.44	73.18	73.1	73.33	*	72.61	72.83	84.37	73.14	71.79	72.8	72.87
Vibrio anguillarum PF4-E1-1.fna	72.3	72.32	72.33	72.3	72.58	72.41	72.33	72.34	72.53	72.25	73.27	73.38	72.28	72.21	72.08	72.52	*	72.2	72.57	72.28	71.47	72.19	72.19
Vibrio cyclitrophicus ECSMB14105.fna	73.65	74.03	73.99	73.96	72.74	74.02	74.03	74.01	73.93	73.58	72.13	72.14	73.54	73.5	73.11	73.12	72.67	*	73.4	72.98	72.52	87.79	87.07
Vibrio scophthalmi 20FBVib0200.fna	73.73	73.87	73.92	73.77	73.15	73.87	73.97	73.93	74.12	73.61	72.79	72.87	73.65	73.54	73.84	84.43	73.04	73.39	*	73.42	72.22	73.12	73.38
Vibrio mytili JXOK01.fna	78.51	78.09	77.48	77.43	73.62	77.22	77.57	77.56	77.22	78.65	72.32	72.46	78.53	78.21	73.07	72.95	72.33	72.74	73.12	*	71.93	72.78	72.93
Vibrio mediterranei Vic-OC-097.fna	71.89	72.05	71.93	71.9	71.34	72.08	72.11	72.17	72.06	71.89	71.12	70.89	71.87	71.75	71.72	71.66	71.37	71.93	71.71	71.75	*	71.83	72.06
Vibrio splendidus 2 C04b.fna	73.67	73.92	74.15	73.97	72.73	74.07	74.16	74.11	73.94	73.63	72.43	72.21	73.52	73.52	73.01	73.16	72.8	87.75	73.26	73.13	72.37	*	88.77
Vibrio crassostreae DSM17220.fna	73.66	74.07	74.27	74.09	72.7	73.79	74.21	74.12	73.85	73.53	72.25	72.2	73.54	73.54	73.02	73.04	72.74	86.98	73.11	73.14	72.45	88.65	*

6b:

ANIm identity (%):	Vibrio para-haemolyticus RIMD 2210633.fna	Vibrio harvey SB1.fna	Vibrio owenssi XSBZ03.fna	Vibrio jasicida 090810c.fna	Vibrio vulnificus ATCC27562.fna	Vibrio rotiferianus B64D1.fna	Vibrio campbellii BoB53.fna	30D07.fna	31D03.fasta	Vibrio natriegens ATCC14048.fna	Vibrio fluvialis ATCC33809.fna	Vibrio furnissii FDAARGOS 777.fna	Vibrio diabolicus FA3.fna	Vibrio alginolyticus E110.fna	Vibrio coralliilyticus Rb102.fna	Vibrio ichthyoenteri ATCC700023.fna	Vibrio anguillarum PF4-E1-1.fna	Vibrio cyclitrophicus ECSMB14105.fna	Vibrio scophthalmi 20FBVib0200.fna	Vibrio mytili JXOK01.fna	Vibrio medi-terranei Vic-OC-097.fna	Vibrio splendidus 2 C04b.fna	Vibrio crassostreae DSM17220.fna
Vibrio parahaemolyticus RIMD 2210633.fna	*	85.47	85.45	85.14	85.28	85.48	85.64	85.6	85.49	85.17	85	84.81	87.7	86.97	85.65	84.89	84.08	84.35	85.32	84.24	85.06	84.44	84.5
Vibrio harvey SB1.fna	85.47	*	90.13	88.61	85.42	89.47	89.77	89.8	88.63	84.84	84.81	84.96	85.8	86.47	86.02	85.05	83.86	84.82	85.96	85.28	85.61	84.54	84.63
Vibrio owenssi XSBZ03.fna	85.43	90.13	*	90.95	85.26	89.42	91.12	91.03	88.73	84.9	84.62	84.47	86.02	87.08	86.13	85.22	83.72	84.87	85.49	84.14	85.46	84.47	84.63
Vibrio jasicida 090810c.fna	85.15	88.61	90.94	*	85.23	88.44	89.22	89.26	88.04	84.64	84.63	84.79	85.49	86.21	85.8	85.25	83.8	84.69	86.16	83.96	85.35	84.54	84.6
Vibrio vulnificus ATCC27562.fna	85.34	85.42	85.27	85.23	*	84.93	85.25	85.31	85.23	84.95	84.75	84.96	85.11	85.08	85.16	84.42	84.57	84.39	84.93	84.33	85.27	84.14	84.19
Vibrio rotiferianus B64D1.fna	85.47	89.48	89.43	88.44	84.92	*	90.32	90.46	91.44	85.1	85.3	84.72	85.9	86.48	86.45	85.2	83.82	84.83	86.09	84.2	85.6	84.76	85.09
Vibrio campbellii BoB53.fna	85.67	89.77	91.12	89.23	85.25	90.32	*	97.2	89.07	84.92	84.82	84.9	85.79	86.46	85.89	85.13	83.83	84.74	85.69	84.11	85.33	84.57	84.89
30D07.fna	85.61	89.81	91.03	89.26	85.3	90.46	97.2	*	89.2	85.06	84.8	84.76	85.81	86.66	85.92	84.99	83.62	84.92	85.77	84.16	85.64	84.69	84.84
31D03.fasta	85.48	88.63	88.73	88.01	85.19	91.43	89.06	89.19	*	85.03	85.43	85.14	85.77	86.25	86.31	85.25	83.86	84.9	85.74	84.19	85.38	84.62	84.91
Vibrio natriegens ATCC14048.fna	85.18	84.86	84.9	84.66	84.93	85.1	84.93	85.03	85.03	*	84.94	84.87	85.09	85.02	85.79	84.89	84.11	84.57	85.51	84.45	85.22	84.6	84.69
Vibrio fluvialis ATCC33809.fna	85	84.81	84.6	84.63	84.75	85.21	84.9	84.82	85.31	85.04	*	87.84	85	84.83	84.91	84.14	83.15	84.14	84.78	84.18	84.52	83.58	84.05
Vibrio furnissii FDAARGOS	84.83	84.97	84.56	84.92	84.97	84.89	84.9	84.9	85.27	84.88	87.84	*	84.79	84.6	84.96	84.27	83.37	84.15	85.32	84.64	84.6	84	84.53
Vibrio diabolicus FA3.fna	87.7	85.8	86.02	85.49	85.11	85.9	85.78	85.79	85.77	85.08	84.83	85.05	*	92.3	85.84	84.87	84.07	84.42	85.39	84.47	85.28	84.37	84.58
Vibrio alginolyticus	86.96	86.47	87.08	86.22	84.99	86.47	86.46	86.64	86.25	85.02	84.84	84.66	92.3	*	85.84	84.86	83.85	84.35	85.35	84.37	85.35	84.13	84.44
Vibrio coralliilyticus Rb102.fna	85.65	86.03	86.13	85.79	85.07	86.45	85.98	85.93	86.33	85.77	84.92	84.92	85.94	85.85	*	84.52	83.83	84.82	85.15	84.8	85.14	84.8	84.7
Vibrio ichthyoenteri ATCC700023.fna	84.89	85.1	85.24	85.29	84.43	85.25	85.15	84.99	85.28	84.9	84.14	84.27	84.89	84.88	84.55	*	83.78	85.11	87.64	84.83	84.85	84.92	85.07
Vibrio anguillarum PF4-E1-1.fna	84.08	83.85	83.71	83.8	84.56	83.82	83.82	83.62	83.86	84.11	83.15	83.35	84.07	83.87	83.86	83.8	*	83.9	84	83.97	84.23	83.91	84.06
Vibrio cyclitrophicus ECSMB14105.fna	84.34	84.84	84.88	84.77	84.32	84.83	84.76	84.96	84.86	84.57	84.09	84.14	84.43	84.36	84.85	85.1	83.89	*	85.15	84.29	84.95	89.3	88.77
Vibrio scophthalmi 20FBVib0200.fna	85.28	85.97	85.49	86.16	84.96	86.08	85.79	85.83	85.84	85.47	84.63	85.42	85.44	85.38	85.11	87.64	83.99	85.14	*	84.77	85.27	85.44	85.73
Vibrio mytili JXOK01.fna	84.24	85.28	84.14	83.96	84.32	84.2	84.11	84.16	84.2	84.43	84.16	84.64	84.47	84.37	84.8	84.83	83.96	84.28	84.78	*	85.38	84.15	84.37
Vibrio mediterranei Vic-	85.13	85.65	85.38	85.36	85.06	85.64	85.37	85.68	85.4	85.23	84.54	84.61	85.28	85.37	85.16	84.86	84.22	84.98	85.28	85.38	*	84.95	85.31
Vibrio splendidus 2 C04b.fna	84.43	84.55	84.48	84.6	84.12	84.76	84.57	84.68	84.62	84.62	83.59	84	84.37	84.18	84.82	84.92	83.87	89.3	85.44	84.17	84.91	*	89.9
Vibrio crassostreae	84.53	84.64	84.62	84.66	84.18	85.05	84.88	84.84	84.85	84.71	83.97	84.5	84.55	84.44	84.69	85.09	84.03	88.77	85.76	84.41	85.27	89.92	*

Supplementary Figure S1. Swimming and swarming motility assays of *Vibrio* strain 30D07 (*V. campbellii*) and 31D03 (*V. rotiferanus*-like) after 24-h incubation. (a) Strain 30D07 in LB medium with 2% NaCl and 0.3% agar at room temperature. (b) Strain 31D03 in LB medium with 2% NaCl and 0.3% agar at room temperature. (c) Strain 30D07 in BHI with 2% NaCl and 0.6% agar at 37°C. (d) Strain 31D03 in BHI with 2% NaCl and 0.6% agar at 37°C.

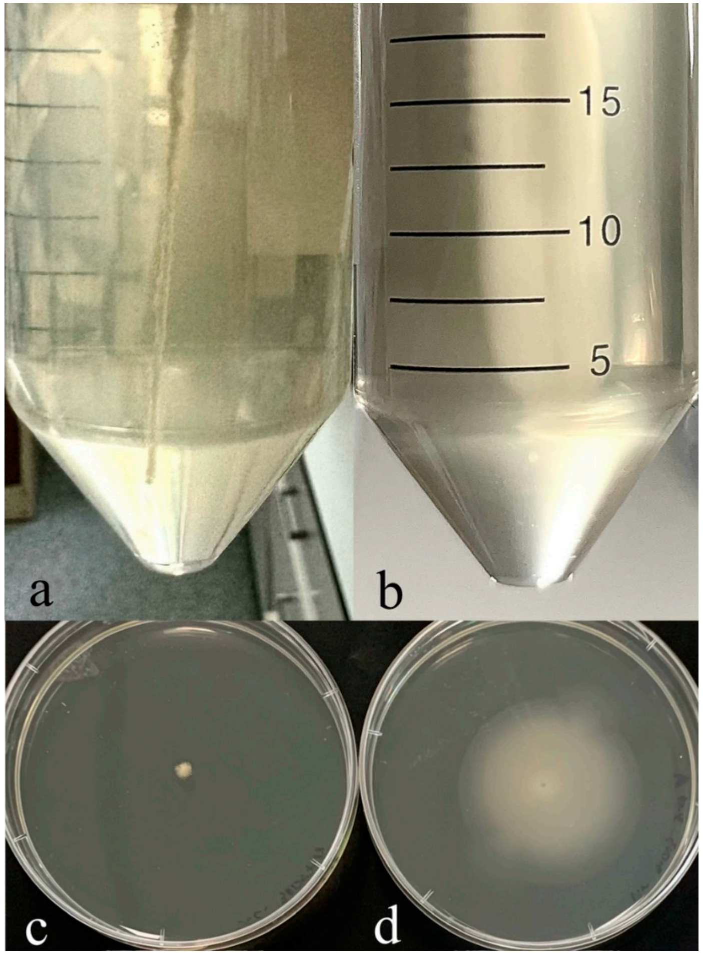

Supplementary Figure S2. Swimming and swarming motility assays of *Vibrio* strain 30D07 (*V. campbellii*) and 31D03 (*V. rotiferanus*-like) after 48-h incubation. (a) Strain 30D07 in LB medium with 2% NaCl and 0.3% agar at room temperature. (b) Strain 31D03 in LB medium with 2% NaCl and 0.3% agar at room temperature. (c) Strain 30D07 in BHI with 2% NaCl and 0.6% agar at 37°C. (d) Strain 31D03 in BHI with 2% NaCl and 0.6% agar at 37°C.

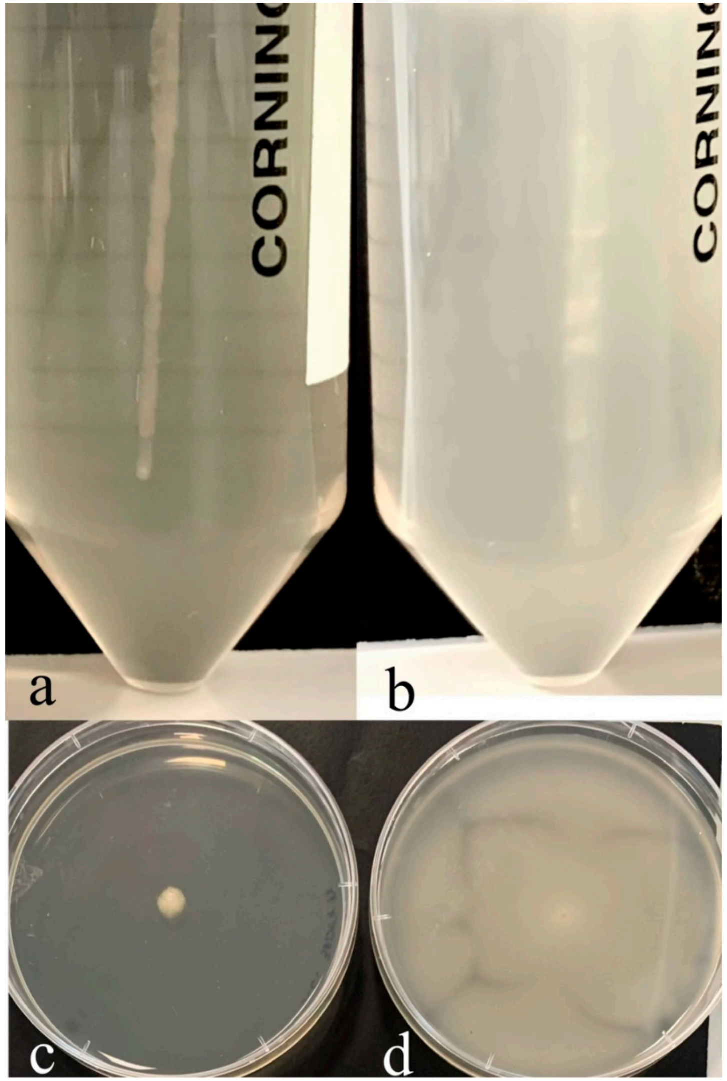

Supplementary Figure S3. The swimming zone developed by *V. campbellii* strain 30D07 in LB medium with 2% NaCl and 0.3% agar after the incubation at room temperature for 7 days.

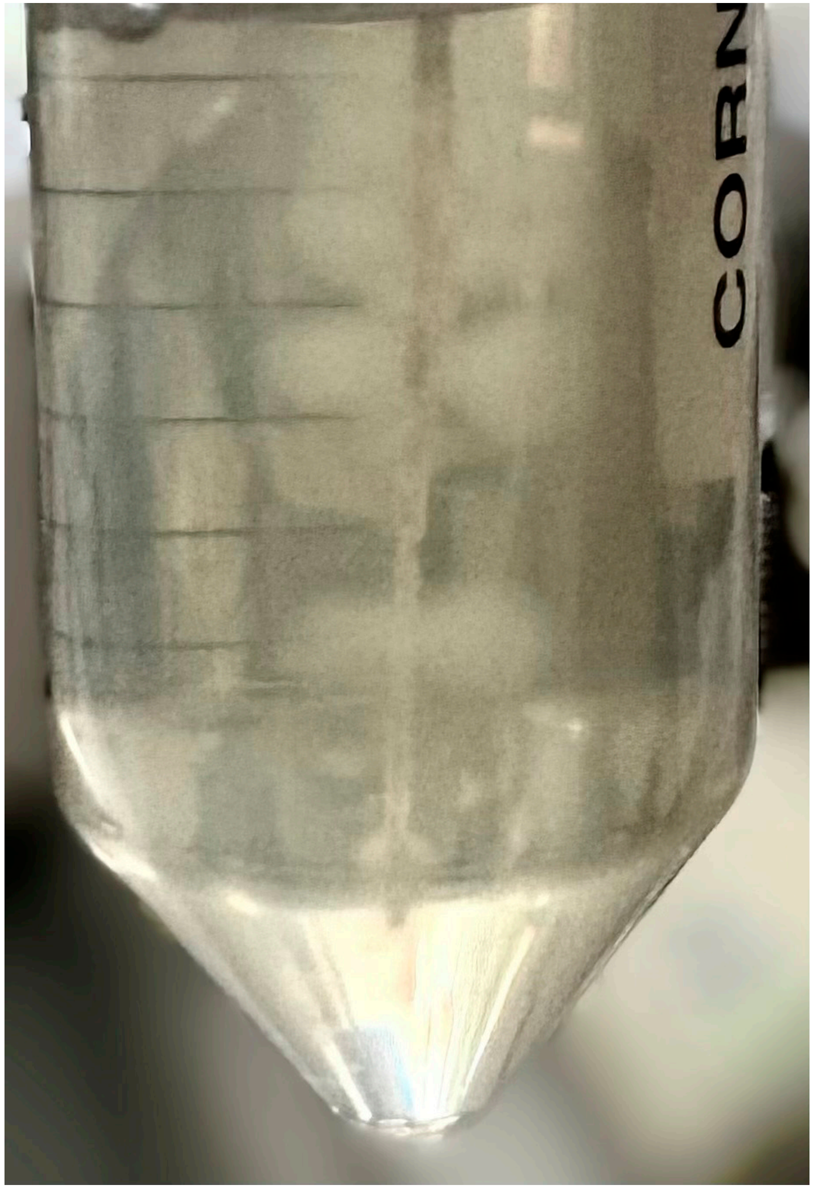

Supplementary Figure S4. Haemolysis of *Vibrio* strain 30D07 (*V. campbellii*) and 31D03 (*V. rotiferanus*-like) on Columbium Horse Blood (5%) agar incubated at 37°C for 24 h. Left: γ-haemolysis showed by strain 31D03; Right: β-haemolysis presented by strain 30D07.

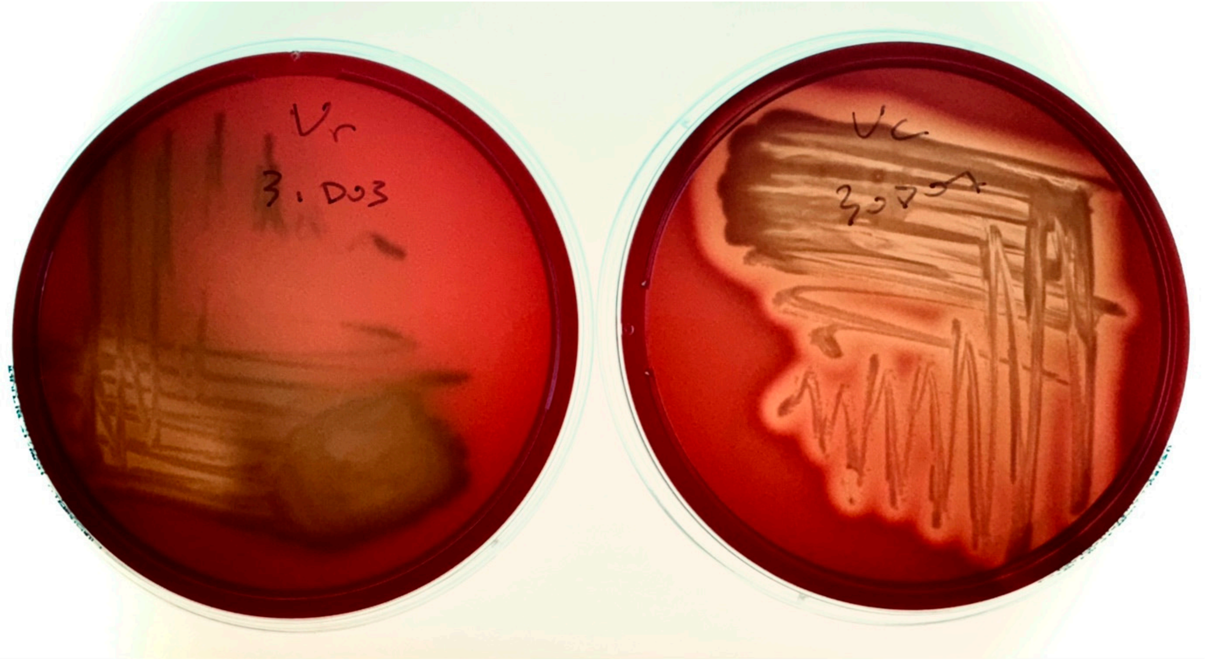

Supplementary Figure S5. The fluorescent effect displayed by *Vibrio campbellii* strain 30D07 on Columbia Horse Blood (5%) agar after 24-h incubation at 37°C.

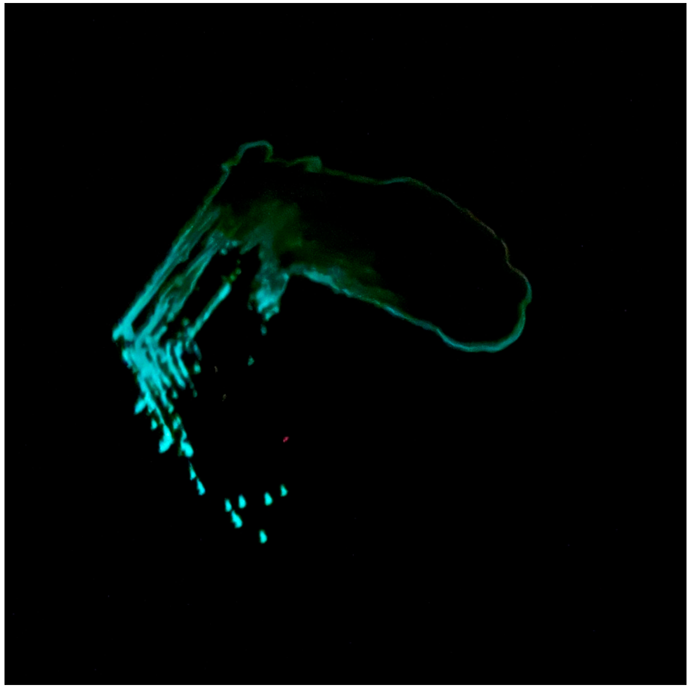

Supplement: Supplementary file 1 [file microorganisms-14-01658-s001.zip › microorganisms-4401154-supplementary.pdf]
